# Current Trends and Confounding Factors in Myoelectric Control: Limb Position and Contraction Intensity

**DOI:** 10.3390/s20061613

**Published:** 2020-03-13

**Authors:** Evan Campbell, Angkoon Phinyomark, Erik Scheme

**Affiliations:** 1 Department of Electrical and Computer Engineering, University of New Brunswick, Fredericton, NB E3B 5A3, Canada; Evan.Campbell1@unb.ca; 2 Institute of Biomedical Engineering, University of New Brunswick, Fredericton, NB E3B 5A3, Canada; aphinyom@unb.ca

**Keywords:** electromyography, EMG, feature extraction, feature selection, myoelectric control, classification, pattern recognition, prosthetics, wearables, amputee

## Abstract

This manuscript presents a hybrid study of a comprehensive review and a systematic (research) analysis. Myoelectric control is the cornerstone of many assistive technologies used in clinical practice, such as prosthetics and orthoses, and human-computer interaction, such as virtual reality control. Although the classification accuracy of such devices exceeds 90% in a controlled laboratory setting, myoelectric devices still face challenges in robustness to variability of daily living conditions. The intrinsic physiological mechanisms limiting practical implementations of myoelectric devices were explored: the limb position effect and the contraction intensity effect. The degradation of electromyography (EMG) pattern recognition in the presence of these factors was demonstrated on six datasets, where classification performance was 13% and 20% lower than the controlled setting for the limb position and contraction intensity effect, respectively. The experimental designs of limb position and contraction intensity literature were surveyed. Current state-of-the-art training strategies and robust algorithms for both effects were compiled and presented. Recommendations for future limb position effect studies include: the collection protocol providing exemplars of at least 6 positions (four limb positions and three forearm orientations), three-dimensional space experimental designs, transfer learning approaches, and multi-modal sensor configurations. Recommendations for future contraction intensity effect studies include: the collection of dynamic contractions, nonlinear complexity features, and proportional control.

## 1. Introduction

Myoelectric control refers to the decoding of motor intent from electrophysiological properties of muscles for use as a control input for some external interface. Despite being a complex and non-stationary signal, surface electromyography (EMG) is leveraged in myoelectric control as a rich source of information. With adequate spatial resolution and a proper EMG pattern recognition pipeline, motions can be deciphered with remarkably high accuracy (>90% accuracy). Myoelectric control has been used in a variety of human-computer interfaces such as upper-limb prostheses or orthoses [1,2], electric wheelchairs [3], muscle-derived speech decoding devices [4,5], virtual reality control devices [6] and other clinical and consumer device designs [7]. While myoelectric control has been touted for decades as an intuitive means of control for assistive-devices, performance of these devices in daily living conditions has been notably inferior to benchmarks achieved in controlled laboratory environments.

The current challenges commonly associated with this lack of reliability in practical conditions can be roughly categorized into four confounding factors.

(a) *Limb position factor*. The muscular activity that maintains limb positions against gravitational force is dependent on the position of the limb. To an even greater degree, during limb motions even larger amounts of supplemental muscle activity is necessary. Additionally, while in different positions the underlying topography of the muscle fibers may shift relative to the electrodes changing the EMG signal measured substantially. These position-dependent muscular activations manifest as artefacts within the EMG signal which lead to confusion between expected muscle activation patterns and observed patterns.
(b) *Contraction intensity factor*. The contraction intensity of motions is subconsciously regulated according to the effort expected to act on the target load. The EMG signal amplitude is directly influenced by this intensity variability, as demonstrated by linear and nonlinear relationships in different works. Additionally, frequency characteristics have been shown to vary according to the contraction intensity elicited. These natural variations in intensity produce largely different muscle activation patterns for the same motion for different loads that may lead to differences between anticipated and observed patterns within a given motion.
(c) *Electrode shift factor*. When the position of an electrode shifts, as is common in socket-based prosthetic devices or circumference-style electrode placement, the underlying musculature changes relative to those electrodes. Subsequently, even in the event the same fibers are underneath the electrode after a shift occurs, a tissue filter effect results in the measured signal being different between electrode locations. Additionally, the change in contact impedance between the electrodes and the skin after a shift occurs introduces further changes in signal properties. As a consequence of these changes, the boundaries defined by a classifier are ill-suited to the properties of the new electrode location.
(d) *Within/between day factor*. The EMG signal is sensitive to many time-varying physiological, biochemical, or anatomical mechanisms [8], for instance blood flow [9]. An additional component of within/between day factor can encompass the change in electrode position when donning and doffing between sessions of myoelectric device use, or a change in perceived contraction intensities between separate uses of the device. These changes greatly influence the applicability of a trained classifier’s decision boundary. This factor has been similarly referred to as the effect of time and is largely responsible for the usability degradation found after prolonged use of myoelectric devices.

Past reviews have provided excellent motivation to investigate and alleviate confounding factors [8,10,11,12,13]; however, a comprehensive overview of all state-of-the-art solutions for confounding factors has not yet been presented. The necessary depth in surveying each confounding factor was accommodated within this survey by being split into two papers. This first paper aims to provide a comprehensive review of the two intrinsic confounding factors: limb position and contraction intensity. The limb position factor introduces a non-conservative domain adaptation between positions that limits operation of myoelectric devices in an unconstrained environment. The contraction intensity factor likewise introduces practical limitations through amplitude variation and frequency shift that standard single intensity level models neglect. The second paper aims to provide a mirrored survey of the current state-of-the-art solutions for the two confounding factors unaddressed in this manuscript: electrode shift and within/between day. The effect on classification performance, experimental design considerations, training procedures, and robust methods of the limb position and contraction intensity factors is the primary focus of this manuscript.

Finally, as an overview, the objective of this article is to illustrate the profound performance degradation caused by signal variability during real world use of myoelectric devices. Within the second section, the traditional topography of EMG pattern recognition architectures is presented. The third and fourth sections provide comprehensive analysis on the performance degradation and current solutions for the limb position and contraction intensity factors. The fifth section provides a summary of critical findings within the analyses and surveyed literature, current limitations of the state-of-the-art, and directions motivating future work.

## 2. EMG Pattern Recognition

**Figure 1:**
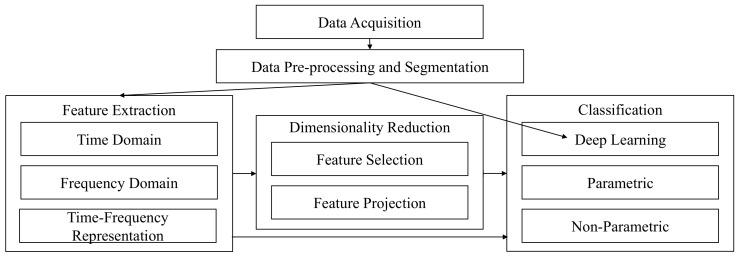
General structure of an EMG pattern recognition pipeline.

The various stages of an EMG pattern recognition system are consistent across the literature, and are comprised of: data acquisition, pre-processing and segmentation, feature extraction, dimensionality reduction, and classification [10,14], as shown in Figure 1. While each of these stages influences the performance of the control system, the quality of the data itself is paramount in order for true motor intent to be decoded from the EMG signal.

### 2.1. Data Acquisition

EMG data are typically acquired through a guided protocol that prompts the end-user to elicit a set of known contractions that will later be recognized for control. During those prompts, patterns of muscle signals are typically recorded from multiple bipolar electrodes placed on the surface of the skin. The SENIAM (Surface ElectroMyoGraphy for the Non-Invasive Assessment of Muscles) guidelines [15] suggest that optimal electrode size and separation are 10 mm and 20 mm, respectively, with electrode pairs oriented parallel to the muscle fiber [16]. In the case of prosthetic devices, electrodes are embedded into the socket of the prosthetic limb. The number and placement of the electrodes are dependent on the classes of motion to be recognized, however, most studies have generally employed either muscle specific placement (determined through palpation) or general placement (circumferential around the forearm [17], for example, or in a grid arrangement). The use of other types of electrode configurations, such as monopolar and Laplacian, have been less successfully employed in myoelectric devices to date. Although data acquisition conventionally occurs only once during the training of pattern recognition-based myoelectric systems, adaptive classifiers which incorporate data over time have also been proposed [18].

#### Datasets

Table 1 summarizes the characteristics of these datasets. Although all data were recorded from intact-limbed subjects, the trends in performance should generalize to clinical populations with some consideration and caution [19,20]. When validating model architectures with amputee populations that have been developed with intact-limbed subject data, a bias is expected (i.e., a relatively constant decrease in accuracy compared to intact-limbed subjects), however, improvements to state-of-the-art models are expected to translate to amputee populations as long as migratory features are not required. For all datasets, the representation of all motion classes was balanced. Preparation of these data sets consisted of removal of power-line interference with notch filters at 50 Hz or 60 Hz, motion-artefact removal with band-pass filters at 20 Hz to 450 Hz, down-sampling of the time-series to 1000 Hz (when relevant), and window segmentation using a window size of 150 ms and increment of 50 ms.

### 2.2. Data Pre-Processing

Pre-processing is used to improve the robustness of the data with respect to potential contaminants [8,25] by increasing the signal-to-noise ratio (SNR) to improve the distinguishing characteristics of the EMG signal. Prior to amplification, the EMG signal is typically between ±5 mV [8]; however, contaminants may corrupt the signal that are several times larger. Examples of contaminants include electrode shift, where the displacement of electrodes results in the surveyed muscle fibers being changed; power-line interference, where the fundamental frequency and harmonics of grid power are introduced into the recorded signal; motion artefact, where electrode-skin interface impedance changes or cable motion introduces low-frequency noise; and electronic noise, where noise is introduced by mismatched electrical components, heating of temperature sensitive components (Johnson–Nyquist noise), or inherent noise in semiconductors (Schottky noise). Among common contaminants, power-line interference is often removed using notch filters at key harmonic frequencies; motion artefacts are removed using a high-pass Butterworth filter with a typical cutoff frequency of 10 or 20 Hz [26]; ECG interference is sometimes removed using high-pass filters with cutoff of 100 Hz [27,28]; and electrical noise may be minimized by using high-performance components and/or a low-pass filter with cutoff-frequency of 450 or 500 Hz (the active energy of the EMG signal during contraction is considered negligible above 500 Hz). Other advanced signal processing techniques employed for EMG denoising include wavelet transform [29,30,31], wavelet packet transform [32,33], empirical mode decomposition [34,35,36], one-dimensional (1D) local binary pattern [37,38,39], and adaptive Wiener filtering [40].

### 2.3. Data Segmentation

Because EMG is a stochastic process, the signal varies naturally over time. This variability results in the signal exhibiting non-stationarity, violating a common assumption among feature extraction methods. Consequently, data segmentation is commonly used to organize the EMG signal into frames of information from which properties can be considered to be weakly or wide-sense stationary. Two main techniques are commonly applied for windowing: adjacent or overlapping window segmentation [41]. Adjacent window segmentation extracts contiguous windows along the time series by incrementing an index by an amount equal to the size of the window. More commonly, overlapping window segmentation is used to increase the density of the decision stream by incrementing neighboring windows by a duration shorter than the window length, resulting in neighboring windows sharing common elements. When smaller increments are used, post-processing techniques can more readily be leveraged to improve the control outputs [42,43,44,45,46,47].

Regardless of the approach, the windowing operation extracts constant duration frames of EMG data that are necessary for feature extraction. Windows are typically enframed into short segments (100–300 ms) [48]. An upper-limit on window length is enforced in real-time myoelectric control, where the time between successive windows and computation time (update rate) cannot exceed 300 ms to avoid perceived delay [14,49].

### 2.4. Feature Extraction

Feature extraction is used to increase the information density in the signal by using representative properties of the segmented windows for classification, rather than the raw samples themselves. Extracting high quality features that possess good class separability, minimal complexity, and are robust to confounding factors is the most important contributor to myoelectric control system performance [50,51]. Features may be categorized according to two criteria: (1) the domain the property is extracted from, or (2) the modality of information the property captures.

Feature extraction is typically performed in three domain representations of the EMG signal: the time domain (TD), the frequency domain (FD), and time-frequency representation (TFR). TD feature extraction refers to procedures that extract properties from the EMG time series in its original form. These are commonly employed features in myoelectric pattern recognition systems due to their high accuracy in low-noise environments and low computational complexity, and provide intuitive information about muscle motor unit recruitment [30,52,53,54,55,56]. The mean absolute value (MAV) feature or root mean square (RMS) feature, for example, is a TD feature that represents the average energy of the EMG signal within a window [57,58]. FD features are extracted from the Fourier transform of the EMG signal to capture information about motor unit recruitment rates and muscle fatigue. The median frequency (MDF) feature, for example, is an FD feature that represents the frequency that divides the total power of the signal into equal upper and lower halves [59,60]. TFR refers to any transformation applied to the EMG signal that incorporates both time and frequency information [33]. The most commonly employed TFR is the wavelet transformation, where the cross-correlation between the EMG signal and a wavelet function is taken across multiple scale/shift configurations. Further dimensionality reduction is commonly required to interpret TFR into meaningful properties, however, this complexity is often justified by demonstrated performance gains [61]. Table 2 contains examples of commonly used features in EMG pattern recognition, with particular emphasis on robustness to confounding factors (see Section 3 and Section 4).

Alternatively, features may be defined according to the properties targeted by their mathematical definitions [52]. The specific modalities of information of these *theoretical* properties include amplitude, variability, stationarity, entropy, linearity, similarity, and frequency. Regardless of designed motivation, characteristics of the EMG signal may result in some features being unable to capture these theoretical characteristics. Such is the case with variance (VAR) which was originally formulated to capture the variability of the signal; however, the zero-mean characteristic of the EMG signal results in VAR being virtually synonymous with other amplitude features. Instead, recent studies have characterized features according to a smaller set of domain-specific categories that have been defined through empirical observation. The specific modalities of information these *empirical* characteristics include are signal amplitude and power, nonlinear complexity and frequency information, time-series modeling, and unique [74].

The selection of appropriate features has a tremendous impact on the performance of any pattern recognition system, and the ideal feature set is heavily dependent on the classification task. Furthermore, the collection of features, and specifically the complementary information that they provide, has led the field to define and adopt known feature sets. For example, the Hudgins’s TD feature set is a widely adopted feature set used for myoelectric control studies with both able-bodied and amputee users. This feature set is comprised of four simple TD features: MAV, ZC, SSC, and WL [57]. Another example, the topologically selected TD feature set (TSTD) is comprised of twelve TD features: MAVFD, DASDV, WAMP, ZC, MFL, SampEn, and TDPSD (6 features) [74]. For a complete depiction of the feature sets contained within this survey, see Table 3.

### 2.5. Dimensionality Reduction

The creation of an appropriate feature set for myoelectric control requires three key considerations: (1) inclusion of features that have high-class discriminatory information, (2) exclusion of features that are heavily correlated with one another, and (3) minimization of the number of features included so as to combat the overfitting influence of the “curse of dimensionality”. Together, these aspects are addressed using dimensionality reduction techniques to improve the robustness of the classification algorithm by removing non-essential features. Although not strictly *required* for myoelectric control, dimensionality reduction can greatly improve classification accuracy and reduce computational cost.

Dimensionality reduction is primarily implemented using one of two methods: *feature selection*, which throws out redundant or less discriminative features, or *feature projection*, which transforms and reduces the dimensionality of the original feature space. Examples of feature selection techniques include sequential forward selection (SFS) and maximum-relevance minimum-redundancy (MRMR) [97]. Examples of feature projection include the unsupervised principle components analysis (PCA) and the supervised uncorrelated linear discriminant analysis (ULDA) [61,98]. Table 4 summarizes various dimensionality reduction techniques, organized by reduction method and whether class labels are required or not (i.e., supervised or unsupervised).

### 2.6. Classification

Classification is the process of inferring the class of an unknown observation using a predictive model trained with earlier observations. Classification algorithms can be divided into two categories: generative classifiers, and discriminative classifiers. Generative classifiers assume that the training data obey some general distribution, and thus can be represented using the parameters of the distribution. This assumption results in decision boundaries that are more robust to minor perturbations, have lower computational complexity, and require less data to reach an optimal performance level. Examples of generative classifiers used in myoelectric control include the naïve Bayes classifier (NB), linear discriminant analysis classifier (LDA), quadratic discriminant analysis classifier (QDA), and Gaussian mixture model (GMM). Discriminative classifiers make no such assumption, and instead directly use training observations to model their discriminant function. These classifiers can have higher specificity than generative classifiers, but typically incur higher computational complexity, require more data to form appropriate discriminant functions, and are therefore subject to over-fitting. Examples of discriminative classifiers used in myoelectric control include *k*-nearest neighbor (*k*NN), random forest (RF), support vector machine (SVM), and sparse representation classification (SRC). Deep learning classifiers are a subset of discriminative classifiers that automatically encode relevant distribution characteristics directly from the raw training data into the bias and weight terms of a neural network architecture by optimizing a loss function. Deep learning classifiers have the highest degree of specificity of all classifiers but require vast amounts of data to reach optimal performance and ensure good generalizability. Examples used in myoelectric control include the recurrent neural network (RNN), long-short term memory (LSTM), probabilistic neural network (PNN), convolutional neural network (CNN), adaptive domain adversarial neural network (ADANN) [108], and recurrent convolutional neural network (RCNN) [109].

### 2.7. Performance Evaluation

The evaluation of myoelectric pattern recognition systems may be performed at different stages of the architecture, namely: (1) at the feature level, (2) after the classification stage, and (3) during feed-forward use, in an online scenario. First, the evaluation of the feature extraction stage quantifies the available features based on the feature space representation of the training data. Considerations for high-quality features include (a) high separability, where clusters of features from each class occupy different regions of feature space; (b) high robustness, where perturbations to the input data result in negligible difference in the feature distributions; and (c) low complexity, where computational overhead of the extraction process does not exceed hardware or real-time requirements [51]. The Davies-Bouldin index (DBI) is often used to quantify these aspects of feature space prior to the actual classification problem. For each cluster, the multi-dimensional standard deviation, $S_{i}$, is computed to describe the magnitude of within-cluster similarity. Additionally, the Euclidean distance between each pair of clusters, $D_{i,j}$ is computed to describe the magnitude of between-cluster similarity (Equation (2)). Within- and between-cluster similarity measures are combined to form a singular measure of overlap, $R_{i,j}$. Finally, the highest magnitude of overlap for each cluster is averaged to quantify the worst-case separability of neighboring class clusters in feature space. Equations (1)–(4) demonstrate how to compute the DBI value. The parameters $x_{j}$, $\mu_{i}$, $N_{i}$, and *K* are the *i*th feature vector, the mean feature vector of the *i*th cluster, the number of feature vectors in the *i*th cluster, and the number of *N* choose 2 cluster combinations, respectively.

(1) $$S_{i}=\sqrt{\frac{1}{N_{i}}\sum_{j=1}^{N_{i}}{\left(x_{j}-\mu_{i}\right)}^{T}\left(x_{j}-\mu_{i}\right)},\;x_{j}\in C_{i}$$

(2) $$D_{ij}=\sqrt{{\left(\mu_{i}-\mu_{j}\right)}^{T}\left(\mu_{i}-\mu_{j}\right)}$$

(3) $$R_{ij}=\frac{S_{i}+S_{j}}{D_{ij}}$$

(4) $$DBI=\frac{1}{K}\sum_{k=1}^{K}\max_{i\neq j}\left(R_{ij}\right)$$

Classifier evaluation quantifies the performance of a myoelectric system according to three measures: (a) the total error rate (TER), (b) active error rate (AER), and (c) robustness. TER denotes the total prevalence of all misclassifications in a test set. In contrast to TER, which is a general descriptive measure, AER quantifies the prevalence of misclassifications that result in incorrect prediction of an active motion class (anything but no motion class) of the control system. This distinction, motivated by the higher cost incurred by moving erroneously than by not moving at all, leads to AER ignoring false negative predictions (incorrect predictions of no motion class). Equations (5) and (6) show the computation of TER and AER, respectively. Parameters *n*, *N*, $p_{n}$, and $l_{n}$ are the index of the test feature vector, the total number of test feature vectors, the class prediction of the classifier for feature vector *n*, and the true class label of feature vector *n*, respectively.

(5) $$\mathrm{TER}=100\%\times (1-\frac{\sum_{n=1}^{N}p_{n}==l_{n}}{N})$$

(6) $$\mathrm{AER}=100\%\times \frac{\sum_{n=1}^{N}p_{n}\neq l_{n}\cap p_{n}\neq \mathrm{NM}}{\sum_{n=1}^{N}p_{n}\neq 1}$$

**Figure 2:**
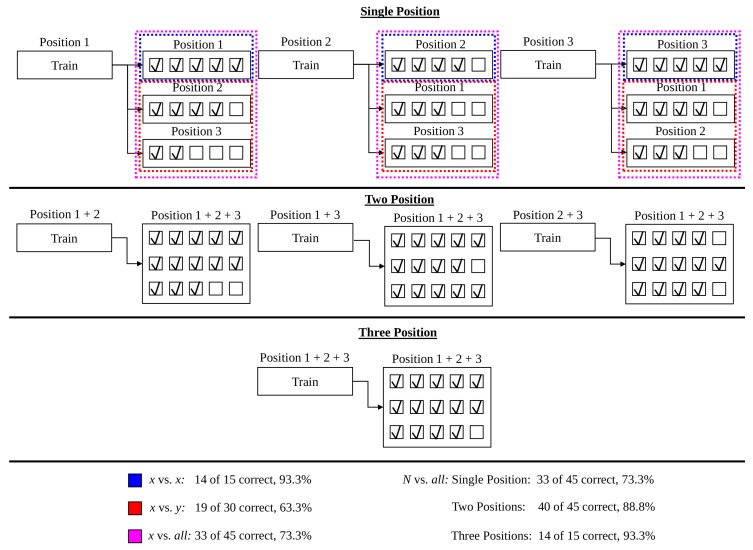
Cross-validation evaluation frameworks, using the limb position effect as an example factor, denoting performance differences for the *x* vs. *x*, *x* vs. *y*, *x* vs. all, and *N* vs. all validation frameworks.

The robustness of a classifier is evaluated using strategic cross-validation of the dataset to highlight sensitivity to factors that may degrade motion classification. Cross-validation frameworks can be categorized into one of four forms: *x* vs. *x*, *x* vs. *y*, *x* vs. *all*, and *N* vs. *all*. The *x* vs. *x* validation framework provides the upper-limit of performance that can be expected when the training conditions identically match the testing conditions, and reflects the ability of the system to recognize testing similar to the training data. The *x* vs. *y* validation framework provides the lower-limit of performance expected when the training and testing conditions are completely disjoint. The difference between the performance of *x* vs. *x* and *x* vs. *y* validations indicate the sensitivity of the myoelectric device to the factor being assessed. The *x* vs. *all* validation framework provides a realistic measure of performance expected when the device is trained in a single condition and tested in multiple other conditions. The *N* vs. *all* validation framework illustrates the trend in performance when increasing the variability in the training set to better match the variability of the testing set. All possible configurations of training and testing conditions are explored for *x* vs. *x*, *x* vs. *y*, *x* vs. *all*, and *N* vs. *all* forms of validation. A demonstration of *x* vs. *x*, *x* vs. *y*, *x* vs. *all*, and *N* vs. *all* forms of validation is included using in Figure 2, using the limb position effect as an example factor.

Online validation is considered to be the most direct measure of the usability of a myoelectric pattern recognition system (other than clinical trials of specific use cases, such as prostheses). In myoelectric control, usability is most often assessed using a Fitts’ law test, which quantifies the information conveyed through a human-computer interface [110]. During this test, trained motions are mapped to cursor movements in a virtual target acquisition environment. Individual target trials are given an index of difficulty (ID) based on the distance to be traveled to acquire the target, *D*, and the size/width of the target, *W*, allowing different configurations to be tested and compared. The range of IDs appropriate for myoelectric control assessment is typically beneath 4 bits, as trial completion rates (within some allowable time) drastically decrease beyond that level [110]. Over multiple repeated trials, the ID is varied and a range of performance metrics (throughput, path efficiency, average speed, overshoot, completion rate) are computed [111]. Throughput is the primary summary metric of Fitts’ law, combining the time taken to acquire the target and the ID to quantify the information transfer rate of the control scheme. Using such a 2 degree of freedom Fitts’ law test, Wurth et al. [112] concluded that both sequential and simultaneous pattern recognition control schemes outperform conventional myoelectric control. Similarly, Robertson et al. [47] used a Fitts’ law test to conclude that rejection of non-confident decisions (to a no motion state) significantly improves the throughput of myoelectric control. Although the Fitts’ law test provides a reasonable measure of usability, any interactions between usability and confounding factor variability have thus far been largely unexplored.

## 3. The Limb Position Effect

### 3.1. Background and Theory

While myoelectric control performance is well-understood in controlled settings, variability in the patterns of EMG can be introduced by a number of different factors. For one, electrodes placed over or adhered to the skin cannot necessarily record the activity of the same underlying musculature under all conditions. Muscle fibers may change positions relative to the sensors as muscle is displaced to provide the tension required during contraction. The shifting of the muscle and subcutaneous tissues then alters the tissue filter effect which modulates the recorded signal. Separately, muscles associated with limb stabilization or locomotion may contract, leading to EMG activity that is unrelated to the motion of interest. This is particularly deleterious when these muscles share common space with intended recording sites. One example of this is the brachioradialis muscle in the forearm which supports both forearm and elbow flexion. In some prosthesis applications, the contact between the electrode and skin may also vary with residual limb position, subject to gravity or device loading.

During motion classification, these factors add ambiguity to the known patterns of muscle co-activation [113,114]. Enforcing rigid constraints on limb position and forearm orientation during data collection manages but also ignores these important sources of signal variability which are present during natural use conditions. Despite improved offline performance, this has been widely reported to lead to a decrease in classification accuracy and robustness during online testing [21,23,113,115,116].

In controlled experiments, the limb position effect is minimized for intra-position classification, when classifiers are trained in the same position as tested. When inter-position classification is performed, however, the limb position effect introduces large amounts of variation that degrade both accuracy and precision [21,113]. When classification is performed across a range of daily conditions, accuracy and precision tend to lie between those of intra- and inter-position because of the presence of some testing positions during training [22]. As the training set grows and includes a greater coverage of testing positions, the accuracy and precision increases towards the upper, intra-position, performance limit. This, however, requires substantial and often not viable collection of vasts amounts of training data.

The degradation from variability in limb position has been demonstrated in both able-bodied and amputee populations. In amputee populations the position effect is thought to be less profound than able-bodied populations, possibly due to shorter muscle lengths and sometimes fixed attachment points on the bone [114]. When a prosthesis is donned, however, the weight of the device compresses muscle fibers, altering the patterns exhibited during muscle contraction. These patterns change as a function of limb position on account of gravitational forces on the device providing relief of pressure, shifting of pressure, or increased pressure on the musculature. Additionally, modern surgical interventions (i.e., agonist-antagonist myoneural interface) avoid anchoring individual muscles to enhance control of the residual muscles [117]. The development of solutions to the limb position effect are therefore necessary for robust operation of myoelectric control systems for both amputees and able-bodied individuals.

### 3.2. Investigating the Limb Position Effect

In this work, a comprehensive investigation was performed to evaluate the limb position effect and serve as a discussion point for comparison to the related literature. Three datasets: (D1) 5 Limb Position [21], (D2) 16 Limb Position [22], and (D3) 3 Forearm Orientation [23], were adopted to demonstrate the effect in different conditions. The high-level signal characteristics of the included datasets can be found in Table 1, with further details available in the original works. The TD [57], LSF4 [70], LSF9 [70], TDPSD [94], and TSTD [74] feature sets outlined in Table 3 were prepared using window lengths of 150 ms and increments of 50 ms.

**Figure 3:**
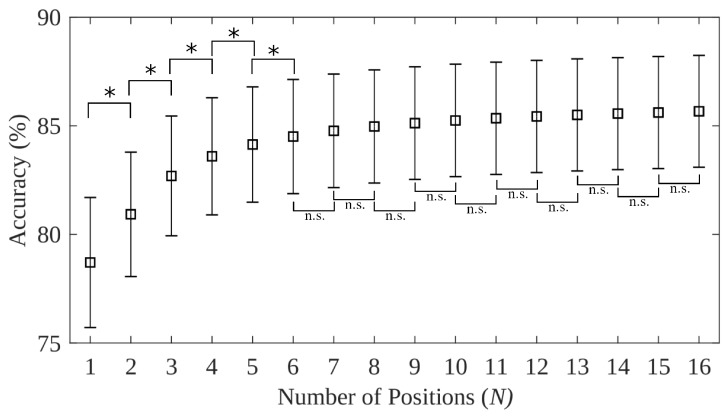
Mean classification accuracies (%) when training with *N* positions in the *N* vs. *all* testing framework using the 16 limb position dataset, the TD feature set and the LDA classifier. Error bars indicate standard error measurements across subjects. Significant differences were denoted by * when the *p*-value was less than 0.05, whereas n.s. indicates no significant difference.

Results were compared for various frameworks found within the limb position literature; (a) *x* vs. *x* (intra-position): training and testing in the same position; (b) *x* vs. *y* (inter-position): training and testing in different positions; (c) *x* vs. *all* (single-position): training in a single position and testing in all positions; and (d) *N* vs. *all* (multi-position): training in multiple positions and testing in all positions. Each testing framework was assessed using leave-one-trial-out cross-validation, within-subject, using *k*NN, LDA, QDA, RF, and linear SVM classifiers. Hyperparameters (SVM: regularization factor, RF: number of trees, and *k*NN: number of neighbors) were selected in preliminary analysis by a grid search algorithm on a subject of each dataset that was excluded from subsequent analysis. This naive selection greatly reduced computation time for analysis. For the sake of clarity, only results from the TD and TSTD feature sets using LDA and SVM classifiers are shown within this section. For the complete set of results, refer to Supplementary Table S1. The results of these testing frameworks are summarized in Table 5 and Figure 3.

*x vs. x (intra-position)*: The results of the *x* vs. *x* testing framework illustrated the conditions under which the majority of myoelectric control experiments have been conducted; those that avoid the limb position effect. In this testing framework, any variability in limb position between training and testing data is minimized, resulting in classification accuracies that are substantially greater than those encountered in clinical practice. As shown in Table 5, this trend of highest accuracy was consistent across feature sets, classifiers, and datasets, often with mean accuracies exceeding 95%. Additionally, the performance was consistently high across different positions, as demonstrated by average standard deviations of 0.6–2.1%. This signifies that repeatability and separability of feature space is consistent across isolated positions, regardless of which position is assessed, as long as single positions are used. This assessment highlights that performance degradation due to the limb position effect is not due to differences in feature separation across different positions. Instead, degradation is more likely influenced by a non-conservative domain adaptation when classification is performed inter-position.

**Figure 4:**
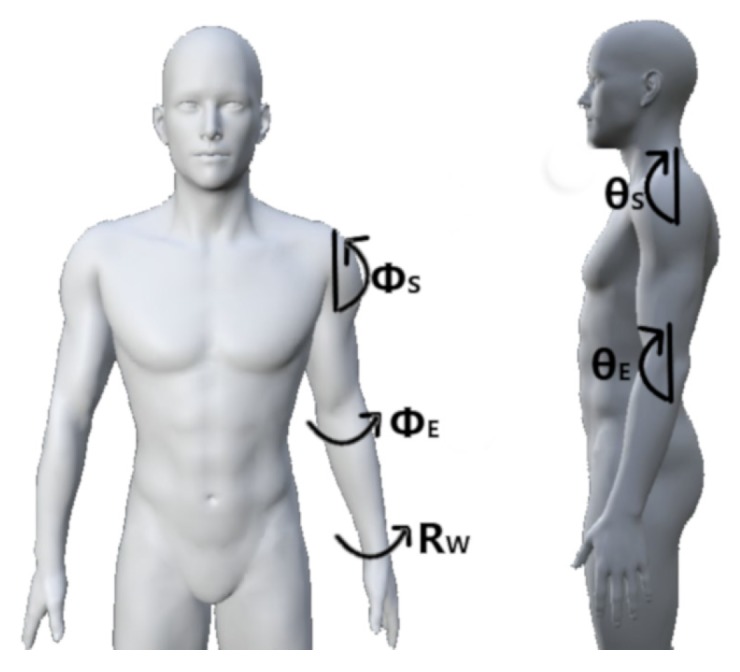
Stratification of limb positions across the literature. P# indicates the label assigned to the position within the survey. $\theta_{S}$, $\Phi_{S}$, $\theta_{E}$, $\Phi_{E}$, and $R_{W}$ indicate angles of shoulder flexion, shoulder abduction, elbow flexion, elbow abduction, and wrist rotation, respectively.

*x vs. y (inter-position)*: The results of the *x* vs. *y* testing framework illustrated the worst-case performance of all. This framework was used in the original demonstration of the limb position effect [113] because the differences due to limb position are maximized between training and test data. As shown in Table 5, the mean accuracy was substantially lower when training and testing positions were different, as demonstrated by a 12.0%, 11.4%, and 54.9% decrease in accuracy compared to intra-position classification on the 5 limb position, 16 limb position, and 3 forearm orientation datasets, respectively. Moreover, the inter-position classification performance on the 3 forearm orientation dataset was substantially lower than that of the 5 and 16 limb position datasets, signifying that the effect of forearm orientation is more profound than other changes in limb position (such as the elbow and shoulder joints). Performance was better when positions were in close proximity to each other, such as P9 with P14, and P2 with P3 position pairs (Figure 4) which averaged 91.8% and 91.6%, respectively, on the 5 limb position dataset. The similarity of feature space between proximal limb positions demonstrates that the domain adaptation between positions manifests gradually as a function of distance or joint space between limb positions. To train and inform a classifier with maximum position variability information with minimal training time, dissimilar positions (those that exhibit a large degree of domain adaptation) are therefore favoured. An example of such a configuration would be a total of six position conditions: P3, P4, P9, and P14 limb positions, while including supination, neutral, and pronation forearm orientations from a single limb position (Figure 4).

*x vs. all (single-position)*: The results of the *x* vs. *all* testing framework illustrate the performance that might be expected during regular operating conditions when trained using a single position, as is commonly seen in clinical and laboratory settings. As expected, the *x* vs. *all* testing framework experienced a large decrease in accuracy and precision compared to *x* vs. *x*, but significantly outperformed the *x* vs. *y* framework. A degradation in performance of 9.5%, 10.5%, and 18.4% was found compared to the intra-position case for the 5 limb position, 16 limb position, and 3 forearm orientations datasets, respectively.

Statistical analysis was conducted using a 4-way ANOVA to identify statistical differences between the various factors of the investigation. A subject-average measure of accuracy was computed for each subject that was the mean accuracy across-cross-validations and testing conditions. This procedure ensures that the dependent variable of interest (accuracy) is independently and identically distributed, an assumption of an ANOVA. A statistical difference was found between testing frameworks (p<0.05), datasets (p<0.05), and classifiers (p<0.05); however, no significant difference was found between feature sets (p=0.18). Of note, all testing frameworks were significantly different, with average accuracies of 88.8%, 76.0%, and 62.8% for the *x* vs. *x*, *x* vs. *all*, and *x* vs. *y* cases, respectively. The 12.8% difference between the *x* vs. *x* and *x* vs. *all* cases highlights the difference between constrained study results and those of more realistic conditions during active use of a device. Post-hoc analysis with Bonferroni correction of the datasets revealed significant differences between all pairs of datasets, with average accuracies of 85.8%, 75.1%, and 66.6% for the 5 limb position, 16 limb position, and 3 forearm orientation datasets, respectively.

The performance of these datasets indicates the amount of variability introduced across the positions surveyed. While the degradation in performance of the 16 limb position dataset was greater than the 5 limb position dataset, it should be noted that it also contained fewer channels of EMG (Table 1). Also, given that it included more positions, the proximity of neighboring positions to one another was closer than those of the 5 limb position dataset. Post-hoc analysis with Bonferroni correction of the classifier factor revealed significant differences between the *k*NN classifier and all other classifiers, with an average accuracy of 79.4%, 77.9%, 77.1%, 76.8%, and 68.0% for the LDA, RF, QDA, SVM, and *k*NN classifiers, respectively. Lorrain et al. [118] also determined that an LDA classifier was the best candidate using the TDAR feature set for motion recognition in the presence of confounding factors with more than 6% improvement when factor-specific variability was introduced to the training set. This is likely because the use of a common (pooled) covariance promotes more general decision boundaries than other discriminative classifiers [42]. Conversely, the SVM classifier is known to perform well with the wavelet feature set [41,118], likely because it explicitly integrates structural risk minimization. Of note, post-hoc analysis revealed no significant differences between feature sets. This is not to say that features do not affect performance, but that the chosen feature sets have all been validated in myoelectric control and possess meaningful and robust discriminatory information.

*N vs. all (multi-position)*: The first three frameworks evaluated accuracy using a single training position; whereas this framework uses multiple training positions. Accuracy was consistently saturated before all limb positions were included. Including more training positions improved accuracy (Figure 3 and Supplementary Figure S2), with no significant improvement found after adding more than 4 positions for the 5 limb position dataset, and more than 6 positions for the 16 limb position datasets. Conversely, no saturation point was reached for the forearm orientation dataset, indicating that all orientations are necessary for an optimal training strategy. Despite these improvements, even when all positions were included in the training set, classification performance of the *N* vs. *all* remained significantly worse than the *x* vs. *x* testing framework (Table 5 and Figure 3). The *N* vs. *all* testing framework accuracy when all positions were included remained 2.3%, 1.5%, and 7.9% worse than the *x* vs. *x* framework across the three datasets.The larger discrepancy between these cases for the 3 forearm orientation dataset suggests again that the perturbation of feature space across forearm orientations is more meaningful than those of limb positions. Inclusion of varied training exemplars can effectively overcome the variability of limb position; however, forearm orientation variability remains a current challenge.

### 3.3. Experimental Protocols

**Figure 5:**
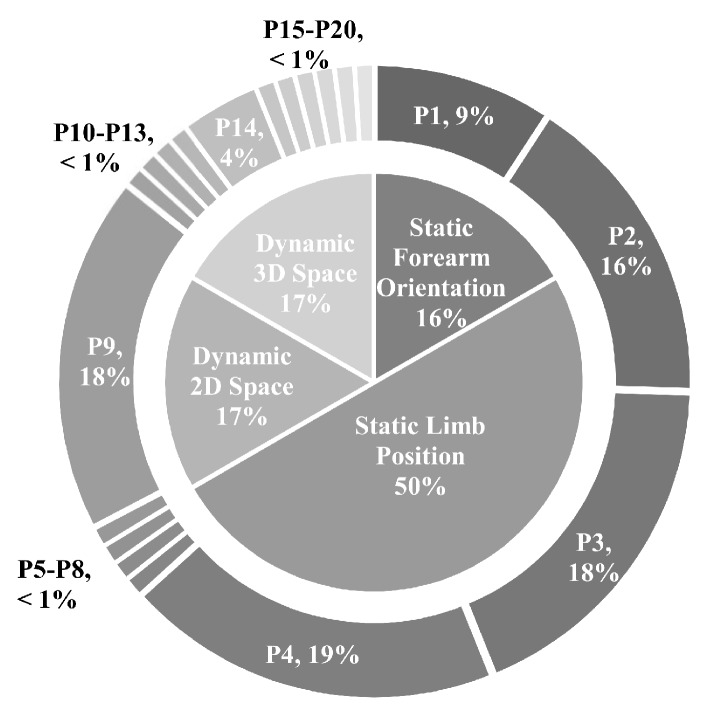
Infographic of the prevalence positions in the literature across 44 articles. Outer ring: limb position and forearm orientation by article count (%). Inner ring: experimental protocol by article count (%).

Researchers have predominantly adopted limb position experimental protocols in one of four forms: (1) the static forearm orientation protocol, (2) the static limb position protocol, (3) the dynamic two-dimensional (2D) space protocol, and (4) the dynamic three-dimensional (3D) space protocol. Within these, researchers have also proposed set positions in which to elicit hand and wrist motions. Compiled from the surveyed literature, Figure 4 contains a list of positions and unifies them under a single frame of reference. Figure 5 summarizes the frequency with which these various positions and experimental design protocols have been used in the literature.

#### 3.3.1. Static Forearm Orientation

Static forearm orientation experiments have been conducted by many researchers [23,119,120,121,122,123,124,125]. Within their protocols, shoulder and elbow joint angles are typically held constant throughout all collections. Variability of position is introduced via articulation of the wrist joint using the supinator and pronator muscles. Positions within these experimental protocols are generally classified as wrist/forearm supination, wrist/forearm neutral, and wrist/forearm pronation. Note that the term forearm rotation is more appropriate, however the term wrist rotation is often used interchangeably.

The primary focus of static forearm orientation experiments is to determine the muscular co-activation patterns that remain consistent across changes in forearm orientation. Myoelectric control often uses electrodes placed within the housing of a prosthetic, or around the forearm in the case of intact-limbed individuals. As muscles involved in supination and pronation of the forearm are located underneath common collection sites, deviations in forearm orientation greatly influence the observed patterns of muscular activation, and hamper classification performance during online and offline classification [122]. Transradial amputees may or may not exhibit forearm orientation degradation depending on the length of the residual limb, where large residual lengths allow forearm pronation/supination and thus invite forearm orientation degradation. Importantly, hand and finger contractions use both intrinsic muscles within the hand and extrinsic muscles within the forearm that are in close spatial proximity to the pronator and supinator muscles that control forearm orientation, making them sensitive to variations in forearm orientation.

#### 3.3.2. Static Limb Position

The most commonly reported limb position experimental protocol in the literature is one that uses static limb positions [21,22,94,113,114,115,116,119,121,122,123,126,127,128,129,130,131,132,133,134,135,136,137,138]. Among the articles surveyed, these make up 50% of all experiments. This protocol prompts subjects to conform to specified wrist, elbow, and shoulder joint angles to reach a characteristic position, commonly those purported to occur during daily activities. While remaining in the desired position, subjects perform repetitions of hand and wrist motion classes. Across the static limb position literature, the average number of positions collected is 8, with a maximum of 91. The most commonly employed positions were P4, P3, P9, P2, P1, and P14 (Figure 4), in descending order of frequency.

The primary focus of static limb position experiments is to determine muscular activation patterns that remain constant across changes in limb positions, or to highlight those that suffer the most. While finger activation patterns are sensitive to variations in forearm orientations due to the proximity of involved muscles, wrist motions are similarly sensitive to changes in limb position due to the use of forearm and shoulder muscles in limb stabilization.

#### 3.3.3. Dynamic 2D Space

**Figure 6:**
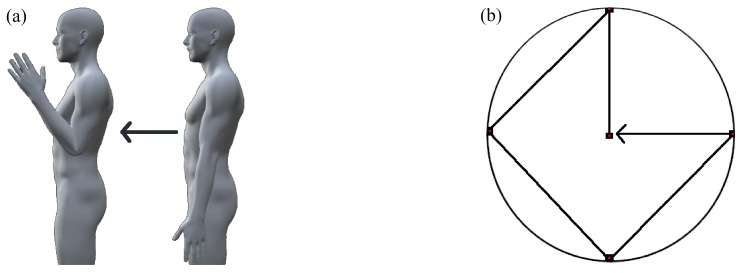
Typical dynamic 2D space experimental protocols: (**a**) connected limb positions experimental protocol; and (**b**) guided path experimental protocol.

Contrary to static forearm orientation and static limb position experimental protocols, dynamic 2D space experimental protocols involve transitions between elbow and wrist joint angles within the collection window [22,116,122,123,130,132,139,140,141,142]. Conventionally, these experiments require subjects to sustain wrist or finger motions while transitioning between two or three limb positions. Figure 6a illustrates an example of dynamic 2D space limb position variation while transitioning between two positions. Figure 6a illustrates an alternative protocol where the subject is given visual guidance through many targets along a trajectory.

The primary focus of dynamic 2D space experiments is to overcome the unreliable performance of myoelectric control systems during online-use. By introducing dynamic changes between limb positions, not only are limb stabilization synergies collected, but muscle contractions involved in wrist and elbow joint articulation are also collected. The measurement of these dynamic factors aids in differentiation between motion classes of interest and limb position changes [116].

#### 3.3.4. Dynamic 3D Space

Dynamic 3D space experiments involve the transition between shoulder, elbow, and wrist joint angles while eliciting a target motion class [22,143,144,145,146,147,148]. Generally, these protocols can be grouped into two subsets: reach-to-grasp, and activities of daily living (ADLs). Within the reach-to-grasp protocol, subjects sequentially transition between a series of phases: a rest phase, arm-extension to object, contact with object, grasp of object, unhand object, return to initial position, rest. Variability can be introduced by requiring different object-related grips (for instance, palmar, pinch, or power grip) or by modifying the elevation or lateral position of the object to introduce shoulder flexion and abduction variability [143,144,145,146]. Within the activities of daily living protocol, progressions between limb positions are designed to model functional tasks: for instance, moving a glass from a tabletop to drinking position or moving from a relaxed position to reaching in a cupboard [22].

The primary focus of dynamic 3D space experiments is to model compound muscle contractions across the entire limb as in real-world functional tasks. The muscle activation patterns of these compound motions have been found to be repeatable under the same conditions; however, when object position or grip style changes, the activation pattern is significantly different [145]. While limited information about shoulder muscle activations can be obtained using forearm electrodes, electrodes situated at the clavicular head of the pectoralis, medial deltoid, infraspinatus, teres major, and superior trapezius muscles have proven successful at distinguishing between shoulder articulations [146]. An alternative application of the reach-to-grasp protocol is the decoding of motor planning using a combination of EMG and electroencephalography (EEG) signals. Neural correlates are defined between the observed EEG signal and key landmarks during the reach-to-grasp motion. Current practices achieve 93.5% accuracy when using EEG to detect the onset of the motion; however, the recognition of the type of grasp using EEG alone has been shown to be a much more difficult task (65.9% for three classes) [149]. This alternative application is greatly motivated by the desire for intuitive neuroprosthetics to restore function to patients with high levels of motor lesion.

### 3.4. State-of-the-Art Approaches

Over the past decade, numerous studies have proposed methods to minimize the degradation caused by variation of limb position. These methods can be roughly categorized into four main approaches: (1) developing feature extraction, dimensionality reduction, and classification algorithms that are less susceptible to the variations of signal patterns between positions; (2) including prior information about the variability of EMG signals by collecting motions in numerous pre-defined static and dynamic positions; (3) improving generalization of features across positions through transfer learning; and (4) incorporating limb position measurements taken from extra sensor modalities.

#### 3.4.1. Robust Algorithms

The first method of minimizing the effect of limb position is the exploration of feature extraction, dimensionality reduction, and classification algorithms that are inherently less susceptible to this effect [23,94,116,130,135,140,150,151,152]. In an exploratory study conducted by Liu et al. [116], repeatability metrics between positions were computed for various commonly used feature sets. The results showed that current feature sets involving Hudgins’ TD features, auto regressivee coefficients, and cepstrum coefficients underwent significant changes when limb position is changed. Khushaba et al. [94,152] expanded upon the Hjorth parameter feature set to extract further time-domain power spectral descriptors (TDPSD) from the time domain using more stable normalization processes. The TDPSD feature set proved to be less variable than other feature sets under the perturbations of limb position [23], and performed similarly in response to force levels [73]. The TDPSD feature set achieved 85%, 91% and 87% accuracy on low, medium, and high intensity contractions between three forearm orientations; whereas, the Hudgins’ set of TD features achieved 78%, 83%, and 85%, respectively. An alternative feature set was proposed by Gu et al. [140], where six band-limited frequency bands were defined using DFT, DWT, and WPT prior to extracting TD features. Between seven static limb positions, SVM classification accuracies of TD features in isolation improved on average by 30% accuracy when extracted from DFT, DWT, and WPT representations instead of from the raw data.

The use of novel classification algorithms has achieved strong performance in the presence of limb position variability. Yu et al. [136] validated the use of a mixed-LDA classifier that used stable representations of motions defined by taking the common-mode of class-specific covariance clusters across all positions. Across five static limb positions, the mixed-LDA classifier achieved 93.6% accuracy opposed to 82.5% accuracy using a standard LDA classifier. Liu et al. [130] found that conditional Gaussian mixture models (cGMM) consistently outperformed conventional LDA during inter-position classification. Across four static limb positions and three dynamic 2D space positions, cGMM had 2% higher accuracy than LDA across all six subjects. Mukhopadhyay et al. [153] achieved 98.9%, 98.7%, 90.6%, 91.8%, and 88.4% accuracy across 5 positions using a DNN, SVM, *k*NN, RF and DT with the TDPSD feature set. Power et al. [154] determined dynamic time waping (DTW) of the RMS value of the signal yielded higher accuracy and lower computational cost than the TDPSD feature set. Liu et al. [155] used a linear-nonlinear cascade regression to simultaneously estimate shoulder, elbow, and wrist joint angles accounting for 93%, 90%, and 84% of the variance in able-bodied subjects, and 85%, 91% and 85% of the variance in stroke subjects, respectively. Betthauser et al. [135] validated the use of a sparse representation classification (SRC), which had found prior success in image detection in cases of heavily occluded objects or missing pixels. The application of SRC yielded significantly better performance across all combinations of training and testing positions (p<0.001). Additionally, the use of extreme learning machines (ELM) in adaptive sparse representation classification (EASRC) greatly reduced computational burden of SRC while maintaining stability under untrained limb positions during an online experiment [150].

These algorithmic approaches to reducing the impact of the limb position effect have indeed enhanced myoelectric control system usability. Nevertheless, the adoption of these more robust methods incurs a tradeoff between the otherwise highest achievable classification accuracy and reliability. In order for existing state-of-the-art configurations to approach the usability of these newer, more robust algorithms, variability between positions may be introduced through strategic training strategies.

#### 3.4.2. Training Strategies

The simplest method to improve predictive power when extrapolating outside of the measured conditions is to collect exemplars of motions classes in the unknown condition [21,23,113,115]. Leveraging this technique, Scheme et al. [113] trained two LDA classifiers: the first was trained using a single limb position, while the second was trained with all eight limb positions. The single-position classifier had an average inter-position accuracy of 65%, whereas the eight position classifier performed substantially better with accuracies between 86–95% among all positions. The effect of forearm orientation was likewise examined by Khushaba et al. [23], where an SVM classifier was trained to detect six motions collected during wrist supination, wrist neutral, and wrist pronation. Although intra-orientation motion recognition had acceptable accuracy (94.2%), no single orientation achieved robustness across positions (67.3%, 60.6%, and 67.6% accuracy across forearm orientation testing frameworks trained in P2, P2s, and P2p, respectively). Further, the magnitude of variability introduced by limb position and by forearm orientation was investigated by Yang et al. [122], with findings that hand and finger gestures were more influenced by forearm orientation than limb position.

Although the benefit of the inclusion of exemplars from multiple positions is evident from these studies, a practical limitation of this approach is the requirement for longer training procedures to inform position tolerant decision boundaries. However, by instead allowing dynamic motions that span static positions, or free motion of the arm in the 3D space, a greater breadth of limb positions and forearm orientations can be measured in less time [22,156,157]. A second consideration of this method involves the possibility that inter-position variability, if in excess, may ambiguate decision boundaries, increasing inter-class error in a single stage classifier [150].

As demonstrated in Section 3.2, Table 5, motion recognition rates are optimal when all possible testing positions are recorded within the training period. While this solution is the conceptually simplest solution to mitigating the limb position effect, a collection period that requires repetition of the training protocol in multiple positions is extremely cumbersome and could result in low user adoption of the system, regardless of the usability once trained. To overcome the limitation of exhaustive training protocols, transfer learning has been proposed as a viable alternative.

#### 3.4.3. Transfer Learning

A third approach to minimizing the limb position effect, while avoiding exhaustive training procedures, is the use of transfer learning. The fundamental aim with transfer learning is to leverage information learned in one domain to improve performance in another. Although now commonly associated with deep learning transfer learning can also be achieved using dimensionality reduction techniques. For example, cannonical correlation analysis (CCA) has been applied to reduce the impact of variability between limb positions. The intention of transformations like CCA is to learn a linear projection between EMG feature space from each position to a reference position. This projection can be applied during device operation when the position is known to minimize the degradation caused by the limb position effect. Also using CCA, Cheng et al. [151] attained similar inter-position and intra-position accuracies and demonstrated that further benefits were gained through using CCA together with uncorrelated linear discriminant analysis (ULDA). The potential of CCA has been demonstrated for various sources of variability, particularly for minimizing the amount of training necessary for novel users by adapting the domain according to subject specific information [129].

In addition to CCA, other feature projection techniques have been explored for myoelectric control. For example, Ishii et al. [121] used a two-stage cascaded bilinear transform, building on the work of Matsubara et al. [158], in an effort to remove the subject and position effect from EMG signals. An emerging avenue of transfer learning for myoelectric control is the strategic retraining of existing neural networks with confounding factor specific data to specialize model weights against the factor’s variability. Although weight-adjustment transfer learning has not been explored as a solution for the limb position effect, supporting work exists for such methodologies applied to reduce inter-subject variability [159,160], and has been validated for use within-subject and between-sessions [161].

#### 3.4.4. Extra Sensor Modalities

A final approach to minimizing the effect of limb position is the use of specialized sensors Fougner et al. [21] defined two frameworks for sensor fusion between EMG and accelerometer/inertial meassurement unit information: (1) a cascaded classifier that, first, classifies position using accelerometer information, then performs motion recognition on the appropriate position-specific classifier; and (2) a single-stage classifier trained using features from accelerometers and EMG signals. The first framework describes multiple position-specific classifiers, each of which require adequate data collection in their respective discrete positions. This ramework is usually preceeded by position classification. The second framework describes a single position-inclusive classifier, where features from all positions are combined to form a single-stage classification scheme. This framework requires features that distinguish position and motion specific information for optimal separability of classes; and thus increases the amount of training data to avoid the curse of dimensionality [132]. Masters et al. [131] explored such frameworks using only EMG to distinguish between positions and found significant improvement over training in the neutral position; however, no significant difference was found between the position-specific and position inclusive frameworks. Further, Shazad et al. [162] determined single-stage classifiers trained with continuous position data outperformed discrete position classifiers with 98.7% and 97.6% accuracies, respectively.

The use of sensors other than accelerometers has been explored to provide positional awareness. Geng et al. [127] added mechanomyography (MMG) signals to augment EMG, yielding improved position specificity. As a result, the MMG-informed classifiers outperformed EMG alone in both the single-stage and two-stage classifier frameworks. Interestingly, Khushaba et al [91] explored graph laplacian (GL) based feature extraction for accelerometer and MMG modalities, and found that GL based feature extraction yielded 93.8% and 94.1% accuracy, respectively, across 40 motion classes. This is in stark contrast to Hudgins’ TD features based EMG classification, which obtained only 66% accuracy. This work suggests that further investigation is required to better understand the discriminative power of these alternative sensors. These results should be interpreted with caution, however, as the dataset used exploits positional variance to enhance separability of motion classes. This is a common trait among human-computer interaction studies, however, biomedical studies motivated by prosthesis control cannot exploit positional variance as motions are required to be elicited regardless of limb position [163].

## 4. Variation in Contraction Intensity

### 4.1. Background and Theory

As with limb position, neglecting variability introduced by muscle contraction intensity can also lead to differences in observed performance between controlled lab results and clinical usability. When repeated measurements of the same motion and contraction intensity are taken, the integrated EMG signal is highly reproducible, attaining correlation coefficients on the order of 0.98 [164,165,166,167]. Signal variability between contraction intensities, however, remains a current challenge for myoelectric control systems in clinical applications. This is particularly important given the widespread use of proportional myoelectric control, which necessitates variations in contraction intensity to control the speed of actuation of a terminal device or cursor [24,168,169,170].

Variability in muscle contraction intensity is modulated physiologically by two factors: motor unit (MU) recruitment threshold, and firing frequency. First, as the intended intensity of the motion increases, so does the neuromuscular signal potential. As the signal increases, greater number of MUs are fired as their recruitment threshold is surpassed, resulting in stronger muscle contractions. Generally, innervation of low-threshold type I (aerobic, fatigue-resistant) MUs persists across all contraction intensities, while higher-threshold type II (anaerobic, fatigue-prone) MUs are additionally recruited when the neural drive is high. Second, the inter-pulse interval of all MUs decreases with contraction intensity, with refractory periods between contractions range from 0.1 s during moderate contraction intensity to 0.025 s during maximum intensity contractions. These two mechanisms introduce signal variability across contraction intensities within the same class of motion.

While the innervation and activation of individual MUs remains largely consistent across intensities, when excluding signal-dependent motor noise [171,172], force production is modulated macroscopically using combinations of these two factors. Additionally, as a consequence of larger signal amplitude, the multiplicative signal-dependent motor noise manifests with larger variance Consequently, the generated EMG signal can vary widely even within the same motion class. This variation is often leveraged to facilitate proportional control in myoelectric classification, or as part of more continuous regression schemes. Notably, the commonly used MAV feature is used, paradoxically, for both classification and proportional control. Nevertheless, without due consideration, the introduced variability can greatly decreased classification performance and overall usability.

### 4.2. Investigating the Force Effect

As before, a comprehensive investigation was performed to evaluate the effect of contraction intensity and to serve as a discussion point for comparison to the related literature. Three datasets: (D4) 7 different % MVC levels [24], (D5) 3 different subjective levels (A) [24], and (D6) 3 different subjective levels (B) [23], were adopted to demonstrate the effect in different collection conditions. The details and pre-processing of the included datasets can be found in Table 1, with further information found in the original works of each dataset. Feature extraction was performed to create TD [57], LSF4 [70], LSF9 [70], TDPSD [94], and TSTD [74] feature sets using a window length and increment of 150 ms, and 50 ms, respectively.

**Figure 7:**
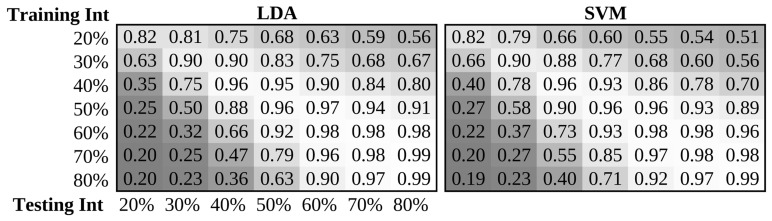
Classification accuracies (%) of the *x* vs. *y* testing framework using the 7% MVC level dataset and the TD feature set.

**Figure 8:**
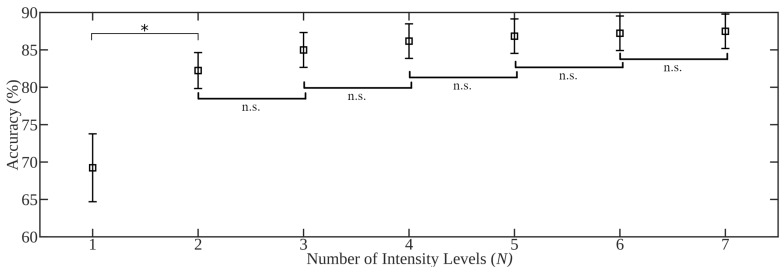
Classification accuracies (%) of the *N* vs. *all* testing framework using the 7 intensity dataset, the TD feature set and the LDA classifier. Error bars indicate standard error measurements for each number of position across subject. Significant differences were denoted by * when the *p*-value was less than 0.05., whereas n.s. indicates no significant difference.

Testing frameworks were defined identically to those in the limb position investigation; however, groups were instead stratified using contraction intensity level. Consequently, motion recognition was performed in the following conditions: (a) *x* vs. *x* (intra-level): training and testing data in the same intensity level; (b) *x* vs. *y* (inter-level): training and testing data in different intensity levels; (c) *x* vs. *all* (single-level): training in a single level and testing in all levels; and (d) *N* vs. *all* (multi-level): training in an increasing number of levels and testing in all levels. As before, within-subject, leave-one-trial-out cross-validation was used to determine performance metrics across five classifiers: *k*NN, LDA, QDA, RF, and SVM. For the complete set of results, refer to Supplementary Table S2. The results of the testing frameworks are summarized in Table 6, Figure 7 and Figure 8.

*x vs. x (intra-level)*: The results of the *x* vs. *x* framework demonstrated the most controlled case (as often done in myoelectric control experiments), using only within-intensity classification. As with the limb position investigation, the accuracies obtained in this frameworks were greater than clinical benchmarks obtained in more practical conditions. As shown in Table 6, the mean intra-level classification accuracy across all subjects was often found to exceed 94%. In contrast to the intra-position frameworks, the intra-level frameworks spanned a higher range of standard deviations in classification accuracy ranging from 1.1–6.3%. These larger standard deviations result from challenges in the precise repeatability of contraction intensity and differences in the separability of feature space across the set of intra-level tests. Unlike the limb position effect, a secondary factor of contraction intensity variability (and thus induced performance degradation) is decreased performance when classifying lower intensity contractions. This difficulty is further magnified for motion classes such as forearm pronation and supination which are not typically associated with high force muscle activations.

*x vs. y (inter-level)*: The results of the *x* vs. *y* framework illustrated the worst-case scenario, with maximum contraction intensity variability between training and testing data. As expected, the *x* vs. *y* framework experienced the worst performance of all testing frameworks. As in Table 6, the mean accuracy attained during motion recognition was substantially lower when training and testing intensity levels were different, demonstrated by a 25.6%, 26.8%, and 29.3% decrease in mean accuracy when performing inter-level classification as opposed to intra-level classification on the 7 intensity, 3 intensity (A), and 3 intensity (B) datasets, respectively. The classification accuracy of the inter-level framework varied widely, with standard deviations in the range of 11.1–26.0%. This variability supports that performance across levels is highly dependent on the selection of the training intensity, as outlined in [24]. The 7 intensity dataset, for instance, had a mean accuracy of 66.3% when using the TSTD feature set with the SVM classifier; however, one train-test pair of intensity levels achieved 94.1% accuracy while another achieved only 28.4%. Additionally, performance was consistently worse when testing on low intensities and training on high intensities when compared with the opposite arrangement of intensities (Figure 7). While the classifier trained on the median intensity level achieved the highest mean accuracy, a classifier based on any single intensity level resulted in likely unusable performance across all feature sets. The collection of multiple intensity levels is therefore required to avoid degrading usability due to muscle contraction intensity variability. Moreover, as the contraction intensity of the training set increases, the performance degradation when testing against even neighboring intensity levels decreased.

*x vs. all (single-level)*: The results of the *x* vs. *all* framework illustrated the impact of contraction intensity variability across a range of contraction intensities day when trained with a single intensity level. As expected, the *x* vs. *all* testing framework experienced a large decrease in accuracy and precision compared with the *x* vs. *x* framework. Despite this decrease, the *x* vs. *all* testing framework still significantly outperformed the *x* vs. *y* testing framework. A degradation in performance of 21.9%, 17.9%, and 19.5% was observed when compared to the *x* vs. *x* testing framework for the 7 intensity, 3 intensity (A), and 3 intensity (B) datasets, respectively.

Statistical analysis of the accuracy of the three single training level testing frameworks was conducted using a 4-way ANOVA. A statistical difference was found for the testing framework (p<0.05), classifier factor (p<0.05), and feature set factor (p<0.05); however, no significant difference was found between datasets (p=0.138). All pairs of testing frameworks were significantly different during post-hoc analyses, where the mean accuracy of *x* vs. *x*, *x* vs. *y*, and *x* vs. *all* was 92.0%, 63.2%, and 71.1%, respectively. As initially hypothesized, the *x* vs. *x* framework attained the best performance among testing frameworks, the *x* vs. *y* framework attained the worst-case performance, and the *x* vs. *all* framework attained better performance than the worst-case scenario when using the same dataset and pattern recognition pipeline. The choice of classifier was found to have an impact on the robustness to contraction intensities, with LDA, QDA, SVM, RF, and *k*NN classifiers attaining mean classification accuracies of 80.5%, 77.2%, 75.2%, 72.2%, and 72.2%, respectively. Notably, LDA achieved the highest accuracy across all classifiers, significantly outperforming all other tested classifiers across all testing frameworks. In contrast to the limb position investigation, the feature set factor was found to be significant. The TD and LSF9 feature sets significantly outperformed the TDPSD feature set with mean classification accuracies of 76.1% and 76.0% compared to 73.8%, respectively. Also unlike the limb position investigation, results across datasets were not found to be significantly different. During this investigation, the amount of variability introduced across the range of intensities of each dataset was relatively consistent, as this variation is more readily modelled experimentally. Therefore, when designing experiments to accommodate the contraction intensity effect, while there is a requirement to survey a range of intensities, the number of levels elicited is of less importance than with limb position.

Of note, this outcome only corresponds to differences in intensity levels in able-bodied subjects. Due to diminished proprioception, muscular atrophy, or surgical outcomes, amputees often report difficulty producing low- and high-intensity contractions [173]. When purposeful regulation is required, the use of visual feedback (such as the overall mean MAV) as an accompanying modality during the training procedure may have a meaningful impact for these users [173]. Likewise, similar difficulties have been reported for other user groups, such as spinal cord injury (SCI) populations, in regulating a targeted intensity level, and in particular, high levels. For these groups, the use of biofeedback with operant conditioning has been used for rehabilitation to increase maximum contractions, and during collection to augment the precision of the recorded intensities [174].

*N vs. all (multi-level)*: In contrast to the first three testing frameworks which used a single training intensity level, the effect of multiple training intensity levels was determined using the *N* vs. *all* testing framework. As shown in Figure 8, accuracy and precision improved on the 7% MVC level dataset as the number of training intensity levels increased (similar relationships on other datasets shown in Supplementary Figure S2). Across all datasets, the minimum number of levels required to obtain near-best performance was two (*p* < 0.05); 20% MVC (low) and 70% MVC (high). This finding corroborates that classification performance can be optimized with few training repetitions as long as the main variability of contraction intensity is included. As expected, there was a positive relationship between the number of training levels included and classification accuracy; however, the mean accuracy when all positions were included remained significantly lower (*p*<0.05) than the *x* vs. *x* framework. This suggests that, despite including the contraction intensity variability from all levels, the classifiers have difficulty in combining this variability completely. The use of these common classifiers to create adequate decision boundaries appears to be limited by the increased entropy introduced by multiple intensities. As in the limb position effect, one solution to this problem is the use of cascaded classifiers, where a preliminary classifier identifies the % MVC and selects the appropriate single level classifier for motion classification.

### 4.3. Experimental Protocols

**Figure 9:**
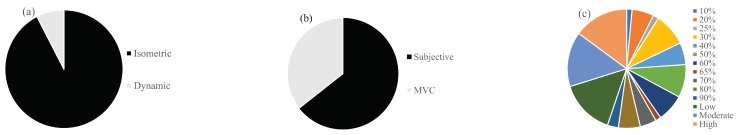
Stratification of contraction intensity experiment characteristics found across the literature: (**a**) muscle contraction types; (**b**) intensity level regulation techniques; and (**c**) intensity levels by article count (18 articles in total).

Across the literature, the experimental designs of contraction intensity works can be divided according to three properties: (1) types of muscle contractions, (2) normalization techniques, and (3) intensity levels selected within the experiment. Figure 9 illustrates the distribution of these three traits across the 18 applicable contraction intensity articles surveyed.

#### 4.3.1. Types of Muscle Contractions

First, the type of contraction elicited during the experiment can have large implications on the interpretation of research outcomes. Isometric, or static, contractions are performed when muscles are under tension but no movement is generated. Isometric contractions are evident when a position is maintained outside of its relaxed state. During these contractions, MUs are sequentially fired at a sufficient rate to maintain tension within the same motion. These contractions model the steady-state phase of the motion, which is the fundamental target of real-time myoelectric methodologies. While experiments with this type of contraction provide a direct measure of the control scheme’s effectiveness in steady-state, natural motions have temporal characteristics and transitions that this format does not capture. Dynamic, isotonic, or ramp contractions, on the other hand, involve changing muscle lengths (shortening or lengthening) and thus movement of a body part is generated. In contrast to isometric contractions, dynamic ramp contractions capture the continuous temporal evolution of natural motions from rest to the desired intensity and sometimes back to no-motion. As such, these contractions are better suited as models for the usability of the device outside of laboratory settings. Training myoelectric devices with such contractions has demonstrated meaningful usability improvements over devices trained with static contractions, where training with dynamic ramps yielded 16.6% higher accuracy than their isometric counterparts [24]. From Figure 9a isometric contractions dominate experimental designs of contraction intensity literature, with only 3 of 18 applicable articles using a dynamic contraction profile.

#### 4.3.2. Intensity Level Normalization Techniques

As in Figure 9b, subjective regulation of contraction intensity is the most ubiquitous regulation technique for contraction intensity studies. This subjective method is achieved by attempting intended levels with no additional feedback provided to the user. This subjective estimation, however, lacks specificity to define precise intensity levels; therefore, subjects are typically told to produce “low” contractions as opposed to, say, 20% MVC. An advantage of subjective estimation, however, is that it is appropriate for gauging the usability of the myoelectric device when trained unaided by external intensity feedback, as would commonly be the case clinically and during everyday use.

In contrast to subjective regulation techniques, objective regulation techniques specify levels of intensity as a normalized percentage of some signal amplitude. The predominant normalization techniques employed include: (1) muscle-specific maximum voluntary isometric contraction (MSMVIC), (2) task-specific maximum voluntary isometric contraction (TSMVIC), and (3) dynamic-range maximum voluntary contraction (DRMVC) [175]. MSMVIC primarily uses a manual muscle test to achieve maximal activation of the target muscle. Manual muscle tests are motions that produce maximal neural activations of the chosen muscle; for an extensive survey of manual muscle tests for various muscles, see Halaki et al [175]. When subjects have appropriate coaching and familiarity with the desired motion, repeatability of contractions is high; however, contractions are often reported exceeding 100% of the maximum activation indicating that selection of manual test may not be appropriate for all subjects [176,177,178]. Studies that assess physiological properties of muscles gravitate towards MSMVC normalization; however, myoelectric control scarcely employs this technique. Conversely, TSMVIC relies on the measurement of maximal muscle contractions during a target task. Myoelectric control predominantly employs TSMVIC to collect training data for intensity robust motion recognition systems, where the training procedure prompts users the elicit maximal contractions of each motion to establish objective reference intensity levels for further repetitions of each respective motion. In contrast to MSMVIC, TSMVIC observes fewer isometric contractions exceeding 100%; however, 100% is still exceeded under dynamic contractions and the relative activation of muscles is variable across-subjects. Finally, DRMVC is suggested as the only appropriate normalization technique for contractions accompanied by movement. In response to changing activation levels during stages of eccentric and concentric contractions [179], reference intensity levels are established using maximal isokinetic contractions. During future myoelectric control experiments where dynamic/ramp contractions are emphasized, it is recommended that DRMVC predominantly be used.

When determining the reference scale using any normalization technique, it is suggested to use at least 3 repetitions, each separated by 2 min, to improve robustness of measurements while limiting fatigue [175,180]. As an alternative to maximal contractions, the use of submaximal contractions (roughly 80% MVC) has been proposed when subjects experience difficulty in total motor recruitment [181]. As shown in Figure 9b subjective regulation dominates the experimental designs in the literature, with only 7 of 18 contraction intensity effect related articles using a motion specific MVC normalization regulation technique.

#### 4.3.3. Intensity Levels

Although signal amplitude is commonly used as a proxy for contraction intensity, other characteristics are also influenced by the intensity of contraction, including the observed innervation zone, the low frequency peak, and the probability distribution function of the signal. As mentioned in Section 4.1, an increase of contraction intensity is manifested through higher MU recruitment and an increase in firing frequency. The increase in recruitment results in more frequent activation of type II MUs. As a consequence of the recruitment of other muscle fibers, the observed innervation zone of the muscles may shift; for example the biceps brachii has been observed to shift up to 2.4 cm over the range of 10%-70% MVC [182]. The increase in firing rate is directly observable via a low frequency peak in the power spectrum of the signal. As the intensity of contraction increases, the frequency of this peak can change from 10 Hz to 40 Hz. The consensus on the overall change in frequency, however, is still under debate. Mean and median frequency have been observed to increase [167,183,184], decrease [59,185,186,187], or remain consistent as intensity increases. The probability distribution function of the EMG signal is typically described as a combination of Gaussian and Laplacian, with their weighting being dependent on the contraction intensity [58]. The distribution has been reported to tend towards towards Laplacian as intensity increases [188], Gaussian as intensity increases [189,190], or towards Laplacian as intensity deviates from 50% [191].

The distribution of intensity levels adopted across studies in the literature is consolidated in Figure 9c. Across all surveyed studies, the mean number of contraction intensity levels was 3.7 ± 2.0, ranging from 2 to 11 levels. The most commonly used number of intensities explored was 3, where those three intensities were low, moderate, and high intensities. Combining the statistical analysis of Section 4.2 with previous outcomes presented in the literature, an appropriate set of intensity levels for an experimental design can be chosen to include 20% MVC (low intensity) and 70% MVC (high intensity) for able-bodied subjects. Additionally, a small sample of current studies successfully incorporate additional contraction variability using ramp contractions. These ramps dynamically range between roughly 20% MVC and 80% MVC and incorporate much of the information without the need for individual repetitions or feedback. Nevertheless, the robustness of myoelectric control systems to dynamic contractions and intentional proportional control remains an active challenge.

### 4.4. State-of-the-Art Approaches

A variety of solutions have been proposed to minimize the impact of contraction intensity variability. Two main methodological approaches have been employed to minimize the variability observed across intensities: (1) training strategies, including prior information about the variability of EMG signals by collecting motions in numerous isometric or dynamic intensity levels; and (2) robust algorithms, including feature extraction, dimensionality reduction, and classification algorithms that are less susceptible to these variations. A third category focuses instead on using the intensity variability itself as an additional control scheme, so as to facilitate proportional control.

#### 4.4.1. Training Strategies

Many experiments have been conducted to determine the optimal training intensity levels for myoelectric control, with little consensus or convergence to a single solution. Khushaba et al. [120] examined the effectiveness of seven feature sets (TDPSD, DFT, DWT, TD, TDAR, MFMF, TDAR with RMS) when trained on low, medium, or high contraction intensities. Classifiers trained with low intensity contractions performed significantly worse than classifiers trained with moderate and high intensity contractions. Across all feature sets, a difference of 10.0% and 4.4% was observed between low intensity trained classifiers and moderate and high intensity counterparts, respectively. Li et al. [192] investigated the robustness of classifiers trained exclusively on low, medium, or high intensity levels and achieved 69%, 81%, and 74% accuracies, respectively, on test sets containing all intensity levels not included in the training set. Similarly, He et al. [96] suggested using the median intensity level along with the range of anticipated test intensities. While the specific intensity level for an optimal single-intensity training procedure differs across the literature, there is agreement that the simplest method of improving robustness to intensity variability is training with multiple intensity levels. There is less research reported, however, on the best combination of intensities with which to train. Scheme et al. [168] proposed training with exemplars of low intensity (20% MVC) and high intensity (80% MVC) to ensure robustness under all intensity levels. Finally, Scheme et al. [10,24] also recommended including ramp contractions within the training set to both capture the natural variability of contraction intensity during regular use, and to expedite the collection process. This approach has since been adopted as best practice by many groups across the field [193,194].

Despite the robustness gained by collecting multiple intensity levels, or these dynamic training regimes, there is motivation to further improve robustness through algorithmic solutions. Consequently, the development of pattern recognition methodologies that are invariant to contraction intensity have been explored.

#### 4.4.2. Robust Algorithms

In contrast to the above training strategies, the aim of robust algorithms is to be inherently less sensitive to variability introduced by different contraction intensities. These methods have typically fallen into three categories: (1) feature extraction, (2) feature projection, and (3) classification algorithms.

Robust feature extraction methods aim to extract features that are resilient to changes in contraction intensity while retaining discriminative motion specific information to facilitate classification. Tkach et al. [95] determined that the selection of appropriate time domain features can improve classification performance in the presence of intensity variability by 16%, with a particular emphasis on the robustness of time-series modeling features, such as autoregressive and cepstral coefficients. Similarly, nonlinear features have been found to outperform linear features (only AR features performed well inter-level), with power spectral entropy, detrended fluctuation analysis (DFA) [77], and maximum fractal length achieving the highest performances in isolation [195]. Nonlinear features were further explored by Iqbal et al. [196], with entropy features derived from the wavelet packet transform outperforming those from DFA.

In addition to evaluation of isolated features, sets of features have also been evaluated. A comparison between TD with KURT and AR with RMS feature sets determined that the former yielded the best performance for two amputee subjects [173]. The use of time domain (TD, TDPSD, TDAR, TDAR with RMS), frequency domain (DFT, MFMF), and time-frequency representation (wavelet) feature sets were compared across 3 intensity levels [23,120]. TDPSD significantly outperformed all other feature sets (*p* < 0.01), while DFT appeared consistently as the second best. In addition, Li et al. [192] explored the use of common spatial patterns extracted from the EMG signal, and found that they performed 5.3% better than the TD feature set. Through the use of an improved DFT, Lv et al. [197] demonstrated greater accuracy using FD features than TD features across intensities. Feature sets that harness nonlinear complexity information, such as fractal features and power spectral entropy with AR, demonstrated the possibility of motion recognition in the absence of reliance on amplitude features; however, this system under-performed compared to TDPSD [195]. Finally, Asogbon et al. [71] examined the combination of contraction intensity variability and subject mobility on myoelectric control. Their proposed feature set, invTDF, outperformed TD by 12% and reported considerably lower across-subject variability of performance.

Alternatively, feature projection techniques selectively retain information from a broader set of features. Motivated by the modularity of motor control for myoelectric control, Atoufi et al. [198] employed NMF to attempt to extract neural primitives from the EMG signal across a range of intensities. In cases of both known and unknown testing intensities, however, MAV outperformed the neural input based feature set. Feature normalization methods demonstrated a meaningful improvement of 5% and 11% for TD and DFTR feature sets, respectively, during the classification of unseen intensity levels [96]. Likewise, the TDPSD feature set has been employed in conjunction with SR [199] to improve robustness to contraction variability within both congenital and traumatic amputee populations. An improvement of 6–8% accuracy was obtained across intensities using TDPSD when compared against VDMOM, AR+RMS, TD, and wavelet feature sets that persisted across subjects and classifiers (LDA, RF, NB, *k*NN) [73].

Finally, classification algorithms have been validated for their robustness to contraction intensity. Al-Timemy et al. [73] determined that the LDA classifier was optimal compared to the RF, naive Bayes, and *k*NN classifier when all intensity levels were included in the training set. Moreover, the application of temporal classifiers like DTW was justified by Powar et al. [200], where DTW performed on RMS representations of amputee and able-bodied EMG signals yielded higher accuracy than conventional classifiers using the TDPSD feature set. Cascaded classification architectures have also been employed to alleviate the impact of changing contraction intensities, with a first stage selecting the appropriate force-specific second-stage motion classifier. Li et al. [201] demonstrated the cascaded classification architecture yielded a 18.1% increase in accuracy when using three second-stage classifiers.

#### 4.4.3. Proportional Control

The variability introduced through muscle contraction intensities may degrade classification performance; however, this phenomenon has also been harnessed to improve control. These proportional control schemes modulate the amount of class-specific output (such as the velocity of a device or cursor) based on the contraction intensity. Conventional myoelectric control systems utilize this within-class variability by mapping an estimate of the EMG amplitude (traditionally MAV or RMS [58]) to the output velocity of the recognized class. Hoozemans et al. [202] predicted the intensity of prehensile motion ranging between 0 N and 300 N using a low-pass representation of the EMG signal from six forearm electrodes obtaining mean differences between predictions and true intensities of 24 N. Three amplitude reliant proportional control schemes were validated using Fitts’ Law tests by Scheme et al [168]: (1) class-specific proportional control (CSPC), (2) auto-calibrated class-specific proportional control (ACSPC), and (3) bounded proportional control between 10% MVC and 70% MVC (BPC). BPC yielded the best performance among the paradigms used, where improvements over CSPC of 40%, 22%, and 72% were found for able-bodied subjects and 21%, 10%, and 44% for amputee subjects in throughput, efficiency, and overshoot, respectively.

While amplitude estimates are widely used within proportional control schemes, other metrics have also been explored. Atoufi et al. [203] explored the use of neural weights produced through NMF to predict contraction intensities of 8 hand and wrist motions using an ANN classifier. Neural weights significantly outperformed MAV between-subjects on metrics of RMSE (0.90 ± 0.43 vs. 1.17 ± 0.64) and R^2^ (0.76 ± 0.22 vs. 0.52 ± 0.49) during intensity estimates. The use of frequency features was explored by Bilodeau et al. [204] where MNF and MDF significantly increased (p<0.05) with an increase in contraction intensity for three quadriceps muscles. In contrast, Throngpanja et al. [59] determined that MNF, MDF, and time-dependent MNF and MDF significantly decrease as contraction intensity increases during isotonic contractions of the biceps brachii. Additionally, Karlsson and Gerdle [205] explored the use of time-frequency representation metrics on the same quadriceps muscles, obtaining R^2^ values of 0.30–0.43, and 0.91–0.93 for time-frequency measures and RMS, respectively.

## 5. Concluding Remarks and Future Recommendations

The primary focus of myoelectric control has historically been the development of human-computer interfaces that improve quality of life for motor-impaired populations. The use of a controlled environment in myoelectric laboratory experiments has led to reports of high performing systems; however, these systems have been found to be less robust in daily life. Accordingly, the field has sought to improve the robustness of myoelectric control under all daily-living scenarios. The primary confounding sources of variance that have been identified including the limb position effect, the contraction intensity effect, the electrode shift effect, and the within/between day effect. This hybrid survey and analysis highlighted the challenges and current solutions for the limb position and contraction intensity effects. Tables of collection characteristics and EMG pipeline components of limb position and contraction intensity studies have been included in Table 7 and Table 8, respectively. The summary findings of this work are outlined as follows:

### 5.1. Limb Position Effect

- An expected 12.8% decrease on average in accuracy is expected when systems trained in a single position are tested with naturally varying limb position. The relative performance between various testing frameworks (found using the 16 static limb position data set) suggests that at most 6 positions are needed to capture the variability of limb positions across the humeral and transverse plane. Four positions are sufficient to achieve best performance in the 5 static limb position data set, which may have more closely modelled the range of positions used in everyday tasks. The impact of forearm orientation variation, however, can not be minimized using a subset of the training positions. The variance across forearm orientations is sufficiently high that the neutral, pronated, and supinated orientations all must be included in the training procedure to achieve best performance. The combination of these findings are unified to suggest that future myoelectric studies include repetitions in 4 limb positions and all orientations, for example using P3, P4, P9, P9s, P9p, and P14.
- The static limb position protocol serves as a strong environment to assess the robustness of novel algorithmic solutions. The usability challenge in the field, however, is the development of systems that are robust to dynamic motions that transpire while moving between positions. For this reason, a heavy focus on 2D and 3D space experimental protocols is recommended in future works.
- The review of state-of-the-art methods reveals an abundance of feature extraction techniques that minimize the positional variance and segment classes effectively, such as the TDPSD feature set. While the LDA classifier does not attain the highest accuracy in highly controlled settings, the classifier outperforms all other classifiers in the presence of the limb position effect. More meaningful improvements are demonstrated by novel classification architectures that yield significantly better performance than conventional architectures, such as SRC or cascade classification strategies. The design of novel feature extraction methods and classification strategies, however, remains a current research challenge to minimize the limb position effect.
- Transfer learning has the potential to reduce the variance across positions through algorithmic approaches such as CCA and the bilinear transform. Given the phenomenal impact transfer learning has had in other fields (e.g., object detection, deep learning), applications of unsupervised transfer learning may lead to drastic improvements in the state-of-the-art in the future.
- The incorporation of accelerometer and MMG signals into traditionally EMG only systems has lead to improvements in segmenting feature space locally according to position. Current practices mainly include single-stage position informed classifiers, or cascade classifiers that perform position and motion recognition independently. The treatment of the accompanying (non-EMG) modality, however, has been limited in most cases. Recent works suggested that the graph laplacian IMU feature provides better accuracy than an EMG feature set alone, revealing a potentially new avenue for feature extraction methods to incorporate into EMG pattern recognition pipelines.

### 5.2. Contraction Intensity Effect

- An expected 20.4% decrease on average in accuracy is expected when systems trained with a single intensity are tested in the context of naturally occurring contraction intensity variation or proportional control. Best performance was achieved when two intensities were included in the training set: 20% MVC and 70% MVC, or low and high subjective intensities. The best single training intensity level determined in the investigation, and across literature, is the median level across the expected range of intensities, however performance was maximized when including examples from across the expected range (statically or dynamically).
- Traditionally, isometric contractions have been used in myoelectric studies. The intensities of these contractions are commonly regulated using task-specific maximum voluntary isometric contraction normalized levels. A focus of future works should be the extension to dynamic contractions, either isokinetic or isotonic, which more accurately model natural motions. Accordingly, the dynamic-range maximum voluntary contraction normalization technique will be key in appropriately scaling intensity levels.
- The selection of an appropriate pattern recognition pipeline can drastically improve robustness to intensity variation. Nonlinear complexity features demonstrate greater robustness to contraction intensity than the traditional use of amplitude features. Feature projection techniques such as NMF and SR are used to improve resilience of features to intensity variability. Again, the LDA classifier outperformed other traditional classifiers in intensity robustness. Futher improvements are found when using cascade classifiers.
- Auto-calibrated class-specific proportional control has been used in clinical applications; however, bounded proportional control appears to outperform this scheme by ensuring that users can leverage the full range of control.

The state of EMG pattern recognition robustness to confounding sources of variability has advanced drastically over the past decade. Challenges remain in striking a balance between the benefit to classification accuracy and decrease in user-acceptance caused by longer, repetitive, unengaging multi-condition training protocols. Algorithmic solutions provide opportunity for improved robustness without additional user-effort, however, novel approaches designed for one confounding source of variability could have unforeseen sensitivities to other confounding factors. The use of offline evaluation in most investigations surveyed here neglect the influence of sensory feedback, which shapes user behaviours during the use of myoelectric control. An online evaluation framework for EMG pattern recognition in the presence of confounding factors is therefore recommended in future works. The usability of myoelectric devices over long durations during activities of daily living (e.g., driving, office-work, exercise, and cooking) remains largely unaddressed and offers an exciting avenue for exploration. Despite the vast advancements summarized here, confounding sources of variability remain a current challenge for EMG pattern recognition usability in such daily living scenarios. Readers with interest in myoelectric control usability in such daily living scenarios are encouraged to consult a similarly structured future work for an extensive overview of effects of electrode shift and between/within day variations.

## Figures and Tables

**Table:** 

**P#**	**Description**	**(** $\theta_{S}$ **,** $\Phi_{S}$ **,** $\theta_{E}$ **,** $\Phi_{E}$ **,** $R_{W}$ **)**
P1	Shoulder relaxed, elbow flexed 45, neutral wrist	(0, 0, 45, 0, 0)
P2	Shoulder relaxed, elbow flexed 90, neutral wrist	(0, 0, 90, 0, 0)
P2s	Shoulder relaxed, elbow flexed 90, supinated wrist	(0, 0, 90, 0, 90)
P2p	Shoulder relaxed, elbow flexed 90, pronated wrist	(0, 0, 90, 0, −90)
P3	Shoulder relaxed, elbow flexed 135, neutral wrist	(0, 0, 135, 0, 0)
P3s	Shoulder relaxed, elbow flexed 135, supinated wrist	(0, 0, 135, 0, 90)
P3p	Shoulder relaxed, elbow flexed 135, pronated wrist	(0, 0, 135, 0, −90)
P4	Shoulder relaxed, elbow relaxed, neutral wrist	(0, 0, 0, 0, 0)
P5	Shoulder hyperextension 30, elbow relaxed, neutral wrist	(−30, 0, 0, 0, 0)
P6	Shoulder hyperextension 30, elbow flexed 90, neutral wrist	(−30, 0, 90, 0, 0)
P7	Shoulder flexion 45, elbow relaxed, neutral wrist	(45, 0, 0, 0, 0)
P8	Shoulder flexion/abduction 45, elbow relaxed, neutral wrist	(45, 45, 0, 0, 0)
P9	Shoulder flexion 90, elbow relaxed, neutral wrist	(90, 0, 0, 0, 0)
P9s	Shoulder flexion 90, elbow relaxed, supinated wrist	(90, 0, 0, 0, 90)
P9p	Shoulder flexion 90, elbow relaxed, pronated wrist	(90, 0, 0, 0, −90)
P10	Shoulder flexion 90, elbow flexion 90, neutral wrist	(90, 0, 90, 0, 0)
P11	Shoulder flexion 90, elbow adduction 90, neutral wrist	(90, 0, 0, −90, 0)
P12	Shoulder flexion/abduction 90, elbow relaxed, neutral wrist	(90, 90, 0, 0, 0)
P13	Shoulder flexion 110, elbow relaxed, neutral wrist	(110, 0, 0, 0, 0)
P14	Shoulder flexion 135, elbow relaxed, neutral wrist	(135, 0, 0, 0, 0)
P15	Shoulder abduction 30, elbow flexion 90, neutral wrist	(0, 30, 90, 0, 0)
P16	Shoulder abduction 45, elbow relaxed, neutral wrist	(0, 45, 0, 0, 0)
P17	Shoulder abduction 75, elbow flexion 90, neutral wrist	(0, 75, 90, 0, 0)
P18	Shoulder abduction 90, elbow relaxed, neutral wrist	(0, 90, 90, 0, 0)
P19	Shoulder abduction 90, elbow flexed 90, wrist neutral	(0, 90, 90, 0, 0)
P20	Torso horizontal, shoulder relaxed, elbow relaxed, neutral wrist	(0, 0, 0, 0, 0)

**Table 1:** Datasets adopted in this work. N, C, M, R, F_s_, and A/D indicate the number of subjects, channels, motions, repetitions, sampling frequency, and resolution of the analog-to-digital converter in bits, respectively. MVC refers to maximal voluntary contraction. Position abbreviations are outlined in Figure 4.

Ref	Dataset	N	C	M	R	F_s_	A/D	Confounding Factors
[21]	D1: 5 Limb Position	17	8	8	10	1000	16	P2, P3, P4, P9, P14
[22]	D2: 16 Limb Position	10	6	8	4	1000	n/a	16 Saggital and Humeral
[23]	D3: 3 Forearm Orientation	10	6	6	3	4000	12	P2s, P2, P2p
[24]	D4: 7% MVC Level	10	8	7	4	1000	16	20:10:80% MVC
[24]	D5: 3 Subjective Level (A)	10	8	7	4	1000	16	Low, Medium, High
[23]	D6: 3 Subjective Level (B)	10	6	6	3	4000	12	Low, Medium, High

**Table 2:** Examples of feature extraction methods employed in myoelectric control.

Ref	Feature Extraction Method	Name	Ref	Feature Extraction Method	Name
[52]	Amplitude of the First Burst	AFB	[53,62]	Sample Entropy	SampEn
[63,64,65]	Difference Absolute Mean Value	DAMV	[53,62]	Approximate Entropy	ApEn
[63,65]	Difference Absolute Standard Deviation Value	DASDV	[51,52]	Willison’s Amplitude	WAMP
[51,52,63]	Difference Log Detector	DLD	[52,53]	Myopulse Percentage Rate	MYOP
[52,63]	Difference Temporal Moment	DTM	[53,66]	Box-Counting Fractal Dimension	BC
[51,52,63]	Difference Variance Value	DVARV	[67,68]	Higuchi Fractal Dimension	HG
[51,52,63]	Difference v-Order	DV	[53,69]	Katz Fractal Dimension	KATZ
[70]	Ł-Score	LS	[71]	Integral Square Descriptor	ISD
[71]	Coefficient of Regularization	COR	[71]	Modified Absolute Square Sum	MDIFF
[71]	Mean Difference Derivative	MDIFF	[72]	Activity	ACT
[72]	Mobility	MOB	[72]	Complexity	COMP
[51,52,64]	Integral of Electromyogram	IEMG	[53,68]	Maximum Fractal Length	MFL
[51,52,63]	Log Detector	LD	[51,63,64]	Autoregressive Coefficients	AR
[63,73]	Second-Order Moment	M2	[63,64]	Cepstral Coefficients	CC
[74]	Mean Absolute Value First Difference	MAVFS	[70]	Mean Squared Ratio	MSR
[52,75]	Modified Mean Absolute Value 1	MMAV1	[51,63,64]	Difference Autoregressive Coefficient	DAR
[52,75]	Modified Mean Absolute Value 2	MMAV2	[63,64]	Difference Cepstral Coeffients	DCC
[51,58,76]	Mean Absolute Value	MAV	[67,77]	Detrend Fluctuation Analysis	DFA
[52,53]	Maximum	MAX	[52,78]	Power Spectrum Ratio	PSR
[52,79]	Multiple Hamming Windows	MHW	[80,81]	Signal to Noise Ratio	SNR
[52,53,79]	Mean Power	MNP	[82,83]	Critical Exponent	CE
[52,79]	Multiple Trapezoidal Windows	MTW	[80,81]	Drop in Power Density Ratio	DPR
[52,76]	Root Mean Square	RMS	[51,52]	Histogram	HIST
[52,79]	Spectral Moment	SM	[84,85]	Kurtosis	KURT
[52,79]	Sum of Squared Integral	SSI	[52]	Mean Absolute Value Slope	MAVS
[52,63]	Temporal Moment	TM	[80,81]	Power Spectrum Deformation	OHM
[52,53,79]	Total Power	TTP	[52,53]	Peak Frequency	PKF
[51,52,63]	Variance	VAR	[53,86]	Power Spectrum Density Fractal Dimension	PSDFD
[51,52,63]	v-Order	V	[84,85]	Skewness	SKEW
[52]	Waveform Length	WL	[80,81]	Signal to Motion Artefact Ratio	SMR
[52,75,87]	Frequency Ratio	FR	[73]	Time Domain Power Spectral Descriptors	TDPSD
[59,60]	Median Frequency	MDF	[52]	Variance of Central Frequency	VCF
[59,60]	Mean Frequency	MNF	[53,88]	Variance Fractal Dimension	VFD
[52]	Slope Sign Change	SSC	[89]	Fused Time Domain Descriptors	FTDD
[51,52]	Zero Crossings	ZC	[29,30]	Discrete Wavelet Transform	DWT
[90]	Discrete Fourier Transform	DFT	[33]	Wavelet Packet Transform	WPT
[91]	Graph Laplacian	GL	[92]	Relative Wavelet Packet Energy	RWPE
[71]	Mean Logrithmic Kernel	MLK			

**Table 3:** Datasets adopted or referenced in this work to investigate confounding factors in myoelectric control. AR4, and AR9 refer to autoregressive coefficients of the fourth and ninth order, respectively.

Ref	Abbrev.	Feature Set	Features
[57]	TD	Hudgin’s Time Domain	MAV, ZC, SSC, WL
[93]	TDAR	Time Domain Autoregressive	MAV, ZC, SSC, WL, AR4
[74]	TSTD	Topologically Selected Time Domain	MAVFD, DASDV, WAMP, ZC, MFL, SampEn,
			TDPSD
[70]	LSF4	Low Sampling Frequency 4	LS, MFL, MSR, WAMP
[70]	LSF9	Low Sampling Frequency 9	LS, MFL, MSR, WAMP, ZC. RMS,
			IEMG, DASDV, VAR
[94]	TDPSD	Time Domain Power Spectral Descriptors	TDPSD
[95]	COMB	Combined	WL, SSC, LD, AR9
[96]	DFTR	Discrete Fourier Transform Representation	DFTR
[71]	invTDF	Inverse Time Domain Features	ISD, COR, MASS, MDIFF, MLK
[72]	Hjorth	Hjorth Parameters	ACT, MOB, COMP

**Table 4:** Organization of dimensionality reduction techniques by reduction type, supervision. SR, NMF, CCA, t-SNE, and MDS refer to spectral regression, non-negative matrix factorization, canonical correlation analysis, t-stochastic neighborhood embedding, and multi-dimensional scaling.

Reference	Reduction	Supervision	Linearity	Algorithm Name	Objective
[99]	Selection	Supervised	–	SFS (Wrapper)	Maximum Classification Accuracy
[99]		Either	–	SFS (Filter)	Optimal Statistical Measure
[97]			–	MRMR	Class Separability - Feature Correlation
[61]			Linear	ULDA	Maximum Separability
[100]		Supervised	Linear	SR	Maximum Separability
[101]	Projection		Linear	NMF	Non-negative Factors
[102]			Linear	CCA	Maximize Domain Correlation
[103]			Linear	PCA	Preserve Variability
[104]		Unsupervised	Nonlinear	Isomap	Preserve Neighborhood Relationships
[105]			Nonlinear	t-SNE	Preserve Neighborhood Relationships
[106]			Nonlinear	Autoencoder	Reduce Reconstruction Loss
[107]			Nonlinear	MDS	Preserve Pairwise Distance

**Table 5:** Classification accuracies (%) of the different testing frameworks for the limb position effect.

Feature Set	Classifier	Test Framework	5 Limb Position	16 Limb Position	3 Forearm Orientation
			Mean±SD (Min, Max)	Mean±SD (Min, Max)	Mean±SD (Min, Max)
TD	LDA	Intra-Position	94.5 ± 1.2 (93.1, 96.0)	86.9 ± 1.9 (83.7, 90.2)	96.1 ± 1.4 (94.6, 97.3)
		Inter-Position	80.2 ± 7.1 (67.7, 92.0)	75.4 ± 6.6 (58.2, 89.4)	36.8 ± 8.6 (27.8, 47.4)
		Single-Position	83.1 ± 2.2 (80.6, 86.6)	76.1 ± 2.9 (70.9, 80.1)	56.6 ± 1.1 (55.5, 57.6)
	SVM	Intra-Position	95.2 ± 0.9 (93.7, 96.1)	85.9 ± 1.8 (83.0, 88.8)	92.3 ± 1.7 (90.7, 94.1)
		Inter-Position	86.5 ± 4.7 (77.5, 93.8)	74.2 ± 6.4 (57.2, 86.4)	36.1 ± 8.8 (25.7, 47.6)
		Single-Position	88.3 ± 0.8 (86.9, 89.1)	74.9 ± 2.6 (70.3, 78.4)	54.8 ± 2.2 (53.1, 57.3)
TSTD	LDA	Intra-Position	96.9 ± 0.6 (96.1, 97.7)	91.2 ± 1.8 (88.7, 94.6)	97.0 ± 1.1 (95.9, 97.9)
		Inter-Position	86.3 ± 5.9 (75.9, 95.6)	79.9 ± 7.0 (57.1, 92.5)	38.2 ± 7.7 (31.1, 50.4)
		Single-Position	88.4 ± 0.9 (87.3, 89.7)	80.6 ± 2.6 (76.1, 83.6)	57.8 ± 1.9 (55.7, 59.4)
	SVM	Intra-Position	94.9 ± 0.6 (93.9, 95.3)	86.7 ± 2.1 (83.5, 90.1)	94.4 ± 1.4 (93.2, 95.9)
		Inter-Position	85.6 ± 5.1 (76.6, 92.7)	75.1 ± 6.5 (59.4, 88.6)	45.8 ± 7.4 (35.6, 54.8)
		Single-Position	87.4 ± 1.0 (86.0, 88.7)	75.9 ± 2.6 (71.6, 79.3)	62.0 ± 1.2 (60.7, 63.1)

**Table 6:** Classification accuracies (%) of the different testing frameworks for the contraction intensity effect.

Feature Set	Classifier	Test Framework	7% MVC Level	3 Subjective Level (A)	3 Subjective Level (B)
			Mean±SD (Min, Max)	Mean±SD (Min, Max)	Mean±SD (Min, Max)
TD	LDA	Intra-Level	94.0 ± 5.8 (83.0, 99.2)	92.7 ± 2.9 (89.5, 95.2)	96.1 ± 2.0 (93.8, 97.8)
		Inter-Level	67.9 ± 26.0 (19.7, 99.0)	67.2 ± 19.2 (36.7, 90.2)	71.4 ± 11.9 (55.5, 81.5)
		Single-Level	71.7 ± 6.6 (61.2, 79.3)	75.7 ± 6.4 (68.4, 79.9)	79.6 ± 5.7 (76.0, 86.2)
	SVM	Intra-Level	94.1 ± 6.3 (81.6, 99.2)	93.4± 3.2 (90.3, 96.7)	92.8 ± 1.8 (91.7, 94.9)
		Inter-Level	67.2 ± 25.1 (19.3, 98.4)	65.7 ± 17.6 (36.7, 85.8)	59.4 ± 12.0 (40.5, 71.2)
		Single-Level	71.0 ± 6.1 (63.0, 78.5)	75.0 ± 4.3 (70.2, 78.7)	70.6 ± 6.0 (64.1, 76.0)
TSTD	LDA	Intra-Level	95.0 ± 5.0 (85.1, 99.2)	94.1 ± 3.4 (91.1, 97.7)	96.9 ± 1.1 (95.7, 97.7)
		Inter-Level	71.2 ± 23.1 (25.7, 98.9)	68.4 ± 15.8 (49.6, 87.8)	69.0 ± 17.4 (40.4, 84.7)
		Single-Level	74.6 ± 7.9 (59.3, 82.5)	77.0 ± 6.0 (70.9, 82.9)	78.3 ± 8.6 (69.3, 86.6)
	SVM	Intra-Level	91.8 ± 5.3 (81.6, 96.1)	91.0 ± 2.9 (88.2, 94.0)	93.8 ± 2.4 (91.3, 96.1)
		Inter-Level	66.3 ± 20.4 (28.4, 94.1)	62.7 ± 13.7 (46.8, 77.8)	62.7 ± 11.1 (45.1, 72.9)
		Single-Level	69.9 ± 8.1 (54.2, 77.8)	72.1 ± 5.2 (66.7, 77.0)	73.1 ± 4.5 (68.8, 77.8)

**Table 7:** Survey of experimental parameters across position effect myoelectric control experiments. Subject Details (A: Amputee, N: Non-amputee, M: Male, F:Female), Motion Details (H: Hand, NM: No motion, F: Forearm, R2G: Reach to Grasp, R: Rotational, Iso: Isometric, Dyn: Dynamic), Electrode Details (ACC: Accelerometer, DP: Dominant Pectoral, DD: Dominant Deltoid, DT: Dominant Trapezius, DF: Dominant Forearm, DW: Dominant Wrist, F: Forearm, DB: Dominant Biceps, AF: Amputated Forarm, nAF: Non-Amputated Forearm, nDF: Non-Dominant Forearm, LF: Left Forearm, A: Amputation Site, Sparse: Flexible Bipolar Configuration, Circ.: Circumference), n/a: Not Applicable or Specified.

Ref.	Subject Details			Motion Details			Electrode Details			Instrumentation Details			Pattern Recognition Architecture		
	No.	Age	Sex	No.	Type	Position	No.	Location	Layout	Fs	Res.	Gain	Win Size/Inc	Features	Classifier
[144]	4N	23.75 ± 2.06	M	3H	Dyn	3D	8	DP, DD, DT	Sparse	2000	n/a	n/a	n/a	MN PK	kMeans
[143]	4N	23.75 ± 2.06	M	3H	Dyn	3D	8	DP, DD, DT	Sparse	2000	n/a	n/a	n/a	MN PK	kMeans
[113]	8N	n/a	7M 1F	7H + NM	Iso	P2 P3 P4 P9 P10 P14 P19 P20	8EMG 2ACC	DF	Circ.	1000	16	n/a	250/50	TD ACC	LDA
[119]	2N	n/a	n/a	8F + NM	Dyn	P2 P2s P2p P3 P3s P3p P9 P9s P9p	4	DW	Circ.	512	n/a	n/a	n/a	ShannonEn	MLF
[206]	3N	23	M	3H	Dyn	3D	5EMG 4ACC	DP DD DT	Sparse	2000	n/a	n/a	100 200 300	MN SB PK	ANN
[115]	5A	20–43	4M 1F	6H + NM	Iso	P1 P2 P3 P4 P9	8	F	Sparse	4000	n/a	n/a	300/200	TD	LDA
[206]	3N	23	M	3H	Dyn	3D	5EMG 4ACC	DP DD DT	Sparse	400	n/a	n/a	100 200 300	MN SB PK	ANN
[21]	17N	18–34	10M 7F	7H + NM	Iso	P2 P3 P4 P9 P14	8EMG 2ACC	DF	Circ.	1000	16	n/a	250/50	TD ACC	LDA
[127]	5A	22–43	4M 1F	6H + NM	Iso	P1 P2 P3 P4 P9	8	F	6 Circ. 2 Sparse	4000 296.3	n/a	n/a	150/100	TD MMG	LDA
[116]	4N	n/a	n/a	4H + NM	Iso	P3 P4	4	DF	Circ.	2000	n/a	n/a	200/50	TD AR	LDA
[145]	5N	21–40	n/a	3 R2G	Dyn	3D	16	DD DT DB DF	Sparse	1000	16	n/a	n/a	Boxplot	LDA QDA kNN ANN SVM RF
[146]	5N	21–40	n/a	3 R2G	Dyn	3D	16	DD DT DB DF	Sparse	1000	16	n/a	n/a	Boxplot	LDA QDA kNN ANN SVM RF
[127]	10N	22–33	3M 7F	6H + NM	Iso	P1 P2 P3 P4 P9	4EMG 4ACC	DF	Sparse	1000	n/a	n/a	150/50	TD	LDA
[94]	8N	20–37	7M 1F	7H + NM	Iso	P2 P3 P4 P9 P14	7	DF	Circ.	4000	12	1000	150/50	TDPSD	SVM
[127]	5A	22–43	4M 1F	6H + NM	Iso	P1 P2 P3 P4 P9	8EMG 8ACC	AF nAF	Sparse	4000 296.3	n/a	n/a	150/100	TD MMG	LDA
[114]	3A 5N	31–42 24–40	2M 1F 2M 3F	6H	Dyn	P9 P15 P18	7EMG	AF, nAF DF, nDF	Circ.	2048	12	2000	100/40	TD AR	MLP
[142]	4N	27–35	3M 1F	R	Dyn	2D	2	DT DB	Sparse	1000	n/a	500	50	TD MPSDMNF MDF RMS TTP	SVM
[132]	10N	19–32	9M 1F	n/a	Dyn	16	6	DF	Circ.	1000	n/a	n/a	200/100	TD	LDA
[207]	12N	19–35	4M 8F	1H	Dyn	3D	5EMG 32EEG	DF	Sparse	500 5000	n/a	n/a	n/a	n/a	n/a
[22]	10N	19–32	9M 1F	n/a	Dyn	16 HP/ SP	6	DF	Circ.	1000	n/a	n/a	200/100	TD	LDA
[130]	6N	n/a	5M 1F	6H + NM	Iso	P3 P4 P9 P18	6	DF	Circ.	2000	n/a	n/a	200/50	AR	LDA CGMM
[157]	8N	25–40	M	14H	Dyn	P15	112	LF	Grid	2048	12	500	n/a	Motor Modules	n/a
[131]	2N	25–25	1M 1F	4H+NM	Iso	2D	8	DF	Circ.	1000	n/a	n/a	200/25	TD	LDA
[152]	11N	20–37	9M 2F	7H + NM	Iso	P2 P3 P4 P9 P14	7	DF	Circ.	4000	12	1000	100/25	TDPSD	SVM LDA KNN ELM

**Table 8:** Survey of experimental parameters across contraction intensity effect myoelectric control experiments. Subject Details (A: Amputee, N: Non-amputee, M: Male, F:Female), Motion Details (H: Hand, NM: No motion, F: Forearm, R2G: Reach to Grasp, R: Rotational, Iso: Isometric, Dyn: Dynamic), Electrode Details (ACC: Accelerometer, DP: Dominant Pectoral, DD: Dominant Deltoid, DT: Dominant Trapezius, DF: Dominant Forearm, DW: Dominant Wrist, F: Forearm, DB: Dominant Biceps, AF: Amputated Forarm, nAF: Non-Amputated Forearm, nDF: Non-Dominant Forearm, LF: Left Forearm, A: Amputation Site, Sparse: Flexible Bipolar Configuration, Circ.: Circumference), n/a: Not Applicable or Specified.

Ref.	Subject Details			Motion Details			Electrode Details			Instrumentation Details			Pattern Recognition Architecture		
	No.	Age	Sex	No.	Type	Intensity	No.	Location	Layout	Fs	Res.	Gain	Win Size/Inc	Features	Classifier
[173]	2A	32,29	2M	4H	n/a	Low Moderate High	12	A	n/a	2000	n/a	1000	160/40	TD + KURT AR(4) + RMS	LDA
[208]	5N	25.8 ± 0.8	3M 2F	7H + NM	n/a	30 60 90	8	DF	n/a	1000	10	0-4.5 V	128/50	TD	LDA
[24]	10N	25–50	7M 3F	6H + NM	n/a	20 30 40 50 60 70 80 ramp	8	DF	n/a	1000	16	n/a	160/16	TD	LDA MV9
[203]	8N	23-53	7M 1F	8H	Dyn	25 50	12	DF	n/a	1000	12	5000	200/50	NMF	ANN
[198]	9N	n/a	n/a	6H + NM	Dyn	20 30 40 50 60 70 80	n/a	n/a	n/a	n/a	n/a	n/a	200/200	NMF	LDA
[120]	10N	20–30	M	6H	n/a	Low Moderate High	6;8	DF	n/a	4000/2000	12;16	1000	150/75	TDPSD DFT WT TD TDAR TDAR2	SVM
[96]	9N	20–30	M	8H + NM	n/a	20 50 80	8	DF	Sparse	2000	n/a	n/a	200/150	cnDFTR gnDFTR TD COMB	LDA
[201]	3N	23–26	n/a	4H + NM	n/a	20 50 80	4	DF	Sparse	1000	n/a	n/a	150/100	TD	Parallel LDA
[23]	12N 1A	20–33	12M 1F	6H	n/a	Low Medium High	6	DF	Sparse	4000/2000	12;16	1000	150/75	TDPSD DFT WT TD TDAR	SVM
[71]	5N	25–32	n/a	n/a	n/a	n/a	6	DF	4 Circ. 2 Sparse	1024	n/a	n/a	150/100	invTDF TD4 AR4 NOV	LDA
[95]	8N	35 ± 15	4M 4F	4F + NM	Iso	25 65	2	DB DT	4x4 Grid	2500	n/a	60 dB	150/75	MAV ZC SSL WL WAMP V-Order LD VAR HIST(9) AR(9) CepC	LDA
[122]	6N	26.5 ± 3.1	M	n/a	n/a	n/a	8	DF	Circ.	2000	n/a	n/a	128/64	TD FD TDF DFT_TD WT_TD WPT_TD	PNN LDA KNN SVM
[197]	8N	20–30	n/a	n/a	n/a	n/a	4EMG 4IMU	DW	Circ.	2000/148	n/a	n/a	200/150	TD iDFTm ACC TD+ACC iDFT+ACC	LDA
[195]	9A	31.8 ± 10.6	7M 2F	6H	Iso	Low Medium High	8	A	Circ.	2000	16	1000	250/125	FFS PSEAR COMB DFA MFL TDPSD PSE AR HG TD	LDA SVM PNN QDA
[196]	9A	31.8 ± 10.6	7M 2F	6H	Iso	Low Medium High	8	A	Circ.	2000	16	1000	250/125	SEN PEN ShannonWPE, LogEnergyWPE NormWPE SureWPE ThresholdWPE	LDA SVM PNN QDA
[200]	9A 10N	31.8 ± 10.6 20–30	7M 2F n/a	6H	Iso	Low Medium High	8 6	A DF	Circ.	2000 4000	16 12	1000	5000 250	RMS TDPSD	DTW

## Supplementary Materials

The following are available at https://www.mdpi.com/1424-8220/20/6/1613/s1, Figure S1: Multi-position accuracy for datasets D1-D3, Figure S2: Multi-level accuracy for datasets D4-D6, Table S1: Intra-, inter-, and single-position accuracy for datasets D1-D3, Table S2: Intra-, inter-, and single-level accuracy for datasets D4-D6.

## References

[1] Stein J., Narendran K., McBean J., Krebs K., Hughes R. (2007). Electromyography-Controlled Exoskeletal Upper-Limb–Powered Orthosis for Exercise Training After Stroke. Am. J. Phys. Med. Rehabil..

[2] Fardipour S., Bahramizadeh M., Arazpour M., Jafarpisheh A.S., Azimian M. (2018). First prototype of EMG-controlled power hand orthosis for restoring hand extension in stroke patients. Proc. Inst. Mech. Eng. Part H J. Eng. Med..

[3] Phinyomark A., Phukpattaranont P., Limsakul C. (2011). A Review of Control Methods for Electric Power Wheelchairs Based on Electromyography Signals with Special Emphasis on Pattern Recognition. IETE Tech. Rev..

[4] Scheme E.J., Hudgins B., Parker P.A. (2007). Myoelectric Signal Classification for Phoneme-Based Speech Recognition. IEEE Trans. Biomed. Eng..

[5] Scheme E., Castillo-Guerra E., Englehart K., Kizhanatham A., Martínez-Trinidad J.F., Carrasco Ochoa J.A., Kittler J. (2006). Practical Considerations for Real-Time Implementation of Speech-Based Gender Detection. Progress in Pattern Recognition, Image Analysis and Applications.

[6] Summa S., Gori R., Freda L., Castelli E., Petrarca M. (2019). Development of a Dynamic Oriented Rehabilitative Integrated System (DORIS) and Preliminary Tests. Sensors.

[7] Gomez-Gil J., San-Jose-Gonzalez I., Nicolas-Alonso L.F., Alonso-Garcia S. (2011). Steering a Tractor by Means of an EMG-Based Human-Machine Interface. Sensors.

[8] Reaz M.B.I., Hussain M.S., Mohd-Yasin F. (2006). Techniques of EMG signal analysis: Detection, processing, classification and applications. Biol. Proced. Online.

[9] Murphy S., Durand M., Negro F., Farina D., Hunter S., Schmit B., Gutterman D., Hyngstrom A. (2019). The Relationship Between Blood Flow and Motor Unit Firing Rates in Response to Fatiguing Exercise Post-stroke. Front. Physiol..

[10] Scheme E., Englehart K. (2011). Electromyogram pattern recognition for control of powered upper-limb prostheses: State of the art and challenges for clinical use. J. Rehabil. Res. Dev..

[11] Samuel O.W., Asogbon M.G., Geng Y., Al-Timemy A.H., Pirbhulal S., Ji N., Chen S., Fang P., Li G. (2019). Intelligent EMG Pattern Recognition Control Method for Upper-Limb Multifunctional Prostheses: Advances, Current Challenges, and Future Prospects. IEEE Access.

[12] Kyranou I., Vijayakumar S., Erden M.S. (2018). Causes of Performance Degradation in Non-invasive Electromyographic Pattern Recognition in Upper Limb Prostheses. Front. Neurorobotics.

[13] Phinyomark A., Campbell E., Scheme E., Naik G. (2020). Surface Electromyography (EMG) Signal Processing, Classification, and Practical Considerations. Biomedical Signal Processing: Advances in Theory, Algorithms and Applications.

[14] Oskoei M.A., Hu H. (2007). Myoelectric control systems—A survey. Biomed. Signal Process. Control.

[15] Hermens H.J., Freriks B., Disselhorst-Klug C., Rau G. (2000). Development of recommendations for SEMG sensors and sensor placement procedures. J. Electromyogr. Kinesiol..

[16] Young A.J., Hargrove L.J., Kuiken T.A. (2011). The Effects of Electrode Size and Orientation on the Sensitivity of Myoelectric Pattern Recognition Systems to Electrode Shift. IEEE Trans. Biomed. Eng..

[17] Phinyomark A., Quaine F., Laurillau Y. (2014). The Relationship between Anthropometric Variables and Features of Electromyography Signal for Human—Computer Interface. Applications, Challenges, and Advancements in Electromyography Signal Processing.

[18] Sensinger J.W., Lock B.A., Kuiken T.A. (2009). Adaptive pattern recognition of myoelectric signals: Exploration of conceptual framework and practical algorithms. IEEE Trans. Neural Syst. Rehabil. Eng..

[19] Campbell E., Phinyomark A., Al-Timemy A.H., Khushaba R.N., Petri G., Scheme E. Differences in EMG Feature Space between Able-Bodied and Amputee Subjects for Myoelectric Control. Proceedings of the 9th International IEEE/EMBS Conference on Neural Engineering (NER).

[20] Campbell E., Phinyomark A., Scheme E. (2003). A Comparison of Amputee and Able-Bodied Inter-Subject Variability In Myoelectric Control. arXiv.

[21] Fougner A., Scheme E., Chan A.D.C., Englehart K., Stavdahl Ø. (2011). Resolving the Limb Position Effect in Myoelectric Pattern Recognition. IEEE Trans. Neural Syst. Rehabil. Eng..

[22] Radmand A., Scheme E., Englehart K. A characterization of the effect of limb position on EMG features to guide the development of effective prosthetic control schemes. Proceedings of the 36th Annual International Conference of the IEEE Engineering in Medicine and Biology Society.

[23] Khushaba R.N., Al-Timemy A., Kodagoda S., Nazarpour K. (2016). Combined influence of forearm orientation and muscular contraction on EMG pattern recognition. Expert Syst. Appl..

[24] Scheme E., Englehart K. (2013). Training Strategies for Mitigating the Effect of Proportional Control on Classification in Pattern Recognition Based Myoelectric Control. J. Prosthet. Orthot..

[25] Chowdhury R.H., Reaz M.B.I., Ali M.A.B.M., Bakar A.A.A., Chellappan K., Chang T.G. (2013). Surface Electromyography Signal Processing and Classification Techniques. Sensors.

[26] Luca C.J.D., Gilmore L.D., Kuznetsov M., Roy S.H. (2010). Filtering the surface EMG signal: Movement artifact and baseline noise contamination. J. Biomech..

[27] Hargrove L., Zhou P., Englehart K., Kuiken T.A. (2009). The Effect of ECG Interference on Pattern-Recognition- Based Myoelectric Control for Targeted Muscle Reinnervated Patients. IEEE Trans. Biomed. Eng..

[28] Zhou P., Lock B., Kuiken T.A. (2007). Real time ECG artifact removal for myoelectric prosthesis control. Physiol. Meas..

[29] Phinyomark A., Limsakul C., Phukpattaranont P. (2011). Application of Wavelet Analysis in EMG Feature Extraction for Pattern Classification. Meas. Sci. Rev..

[30] Phinyomark A., Nuidod A., Phukpattaranont P., Limsakul C. (2012). Feature extraction and reduction of wavelet transform coefficients for EMG pattern classification. Elektron. Elektrotech..

[31] Phinyomark A., Phukpattaranont P., Limsakul C. (2011). Wavelet-based denoising algorithm for robust EMG pattern recognition. Fluct. Noise Lett..

[32] Wang G., Wang Z., Chen W., Zhuang J. (2006). Classification of surface EMG signals using optimal wavelet packet method based on Davies-Bouldin criterion. Med. Biol. Eng. Comput..

[33] Englehart K., Hudgins B., Parker P., Stevenson M. (1999). Improving myoelectric signal classification using wavelet packets and principal components analysis. Proc. IEEE.

[34] Andrade A.O., Nasuto S., Kyberd P., Sweeney-Reed C.M., Van Kanijn F. (2006). EMG signal filtering based on empirical mode decomposition. Biomed. Signal Process. Control.

[35] Zhang X., Zhou P. (2013). Filtering of surface EMG using ensemble empirical mode decomposition. Med. Eng. Phys..

[36] Naik G.R., Selvan S.E., Nguyen H.T. (2015). Single-channel EMG classification with ensemble-empirical-mode- decomposition-based ICA for diagnosing neuromuscular disorders. IEEE Trans. Neural Syst. Rehabil. Eng..

[37] Tabatabaei S.M., Chalechale A. (2019). Local binary patterns for noise-tolerant sEMG classification. Signal Image Video Process..

[38] Ertuğrul Ö.F., Kaya Y., Tekin R. (2016). A novel approach for SEMG signal classification with adaptive local binary patterns. Med. Biol. Eng. Comput..

[39] Tabatabaei S.M., Chalechale A. One Dimensional Second Order Derivative Local Binary Pattern for Hand Gestures Classification Using sEMG Signals. Proceedings of the 8th International Conference on Computer and Knowledge Engineering (ICCKE).

[40] Liu L., Liu P., Clancy E.A., Scheme E., Englehart K.B. Whitening of the electromyogram for improved classification accuracy in prosthesis control. Proceedings of the Annual International Conference of the IEEE Engineering in Medicine and Biology Society.

[41] Englehart K., Hudgins B. (2003). A robust, real-time control scheme for multifunction myoelectric control. IEEE Trans. Biomed. Eng..

[42] Campbell E.D., Phinyomark A., Scheme E. Linear Discriminant Analysis with Bayesian Risk Parameters for Myoelectric Control. Proceedings of the IEEE Global Conference on Signal and Information Processing (GlobalSIP) (GlobalSIP 2019).

[43] Khushaba R.N., Kodagoda S., Takruri M., Dissanayake G. (2012). Toward improved control of prosthetic fingers using surface electromyogram (EMG) signals. Expert Syst. Appl..

[44] Amsüss S., Goebel P.M., Jiang N., Graimann B., Paredes L., Farina D. (2014). Self-Correcting Pattern Recognition System of Surface EMG Signals for Upper Limb Prosthesis Control. IEEE Trans. Biomed. Eng..

[45] Al-Timemy A.H., Bugmann G., Escudero J. (2018). Adaptive Windowing Framework for Surface Electromyogram- Based Pattern Recognition System for Transradial Amputees. Sensors.

[46] Robertson J.W., Englehart K.B., Scheme E.J. Rejection of Systemic and Operator Errors in a Real-Time Myoelectric Control Task. Proceedings of the 40th Annual International Conference of the IEEE Engineering in Medicine and Biology Society (EMBC).

[47] Robertson J., Scheme E., Englehart K. (2018). Effects of Confidence-Based Rejection on Usability and Error in Pattern Recognition-Based Myoelectric Control. IEEE J. Biomed. Health Inform..

[48] Farfán F.D., Politti J.C., Felice C.J. (2010). Evaluation of EMG processing techniques using information theory. Biomed. Eng. Online.

[49] Farrell T.R., Weir R.F. (2007). The optimal controller delay for myoelectric prostheses. IEEE Trans. Neural Syst. Rehabil. Eng..

[50] Boostani R., Moradi M.H. (2003). Evaluation of the forearm EMG signal features for the control of a prosthetic hand. Physiol. Meas..

[51] Zardoshti-Kermani M., Wheeler B.C., Badie K., Hashemi R.M. (1995). EMG feature evaluation for movement control of upper extremity prostheses. IEEE Trans. Rehabil. Eng..

[52] Phinyomark A., Phukpattaranont P., Limsakul C. (2012). Feature Reduction and Selection for EMG Signal Classification. Expert Syst. Appl..

[53] Phinyomark A., Quaine F., Charbonnier S., Serviere C., Tarpin-Bernard F., Laurillau Y. (2013). EMG feature evaluation for improving myoelectric pattern recognition robustness. Expert Syst. Appl..

[54] Zhang X., Wang Y., Han R.P.S. Wavelet transform theory and its application in EMG signal processing. Proceedings of the Seventh International Conference on Fuzzy Systems and Knowledge Discovery.

[55] Duan F., Dai L., Chang W., Chen Z., Zhu C., Li W. (2016). sEMG-Based Identification of Hand Motion Commands Using Wavelet Neural Network Combined With Discrete Wavelet Transform. IEEE Trans. Ind. Electron..

[56] Ren X., Hu X., Wang Z., Yan Z. (2006). MUAP extraction and classification based on wavelet transform and ICA for EMG decomposition. Med. Biol. Eng. Comput..

[57] Hudgins B., Parker P., Scott R.N. (1993). A new strategy for multifunction myoelectric control. IEEE Trans. Biomed. Eng..

[58] Phinyomark A., Quaine F., Laurillau Y., Thongpanja S., Limsakul C., Phukpattaranont P. (2013). EMG amplitude estimators based on probability distribution for muscle–computer interface. Fluct. Noise Lett..

[59] Thongpanja S., Phinyomark A., Phukpattaranont P., Limsakul C. (2013). Mean and Median Frequency of EMG Signal to Determine Muscle Force Based on Time-Dependent Power Spectrum. Elektron. Elektrotech..

[60] Thongpanja S., Phinyomark A., Hu H., Limsakul C., Phukpattaranont P. (2015). The effects of the force of contraction and elbow joint angle on mean and median frequency analysis for muscle fatigue evaluation. ScienceAsia.

[61] Phinyomark A., Hu H., Phukpattaranont P., Limsakul C. (2012). Application of Linear Discriminant Analysis in Dimensionality Reduction for Hand Motion Classification. Meas. Sci. Rev..

[62] Richman J.S., Moorman J.R. (2000). Physiological time-series analysis using approximate entropy and sample entropy. Am. J. Physiol.-Heart Circ. Physiol..

[63] Phinyomark A., Quaine F., Charbonnier S., Serviere C., Tarpin-Bernard F., Laurillau Y. (2014). Feature extraction of the first difference of EMG time series for EMG pattern recognition. Comput. Methods Programs Biomed..

[64] Park S.H., Lee S.P. (1998). EMG pattern recognition based on artificial intelligence techniques. IEEE Trans. Rehabil. Eng..

[65] Kim K.S., Choi H.H., Moon C.S., Mun C.W. (2011). Comparison of k-nearest neighbor, quadratic discriminant and linear discriminant analysis in classification of electromyogram signals based on the wrist-motion directions. Curr. Appl. Phys..

[66] Gitter J.A., Czerniecki M.J. (1995). Fractal analysis of the electromyographic interference pattern. J. Neurosci. Methods.

[67] Phinyomark A., Phukpattaranont P., Limsakul C., Phothisonothai M. (2011). Electromyography (EMG) signal classification based on detrended fluctuation analysis. Fluct. Noise Lett..

[68] Arjunan S.P., Kumar D.K. (2010). Decoding subtle forearm flexions using fractal features of surface electromyogram from single and multiple sensors. J. Neuroeng. Rehabil..

[69] Gupta V., Suryanarayanan S., Reddy N.P. (1997). Fractal analysis of surface EMG signals from the biceps. Int. J. Med. Inform..

[70] Phinyomark A.N., Khushaba R., Scheme E. (2018). Feature Extraction and Selection for Myoelectric Control Based on Wearable EMG Sensors. Sensors.

[71] Asogbon M.G., Samuel O.W., Geng Y., Idowu P.O., Chen S., R N.G., Feng P., Li G. Enhancing the Robustness of EMG-PR Based System against the Combined Influence of Force Variation and Subject Mobility. Proceedings of the 2018 3rd Asia-Pacific Conference on Intelligent Robot Systems (ACIRS).

[72] Hjorth B. (1970). EEG analysis based on time domain properties. Electroencephalogr. Clin. Neurophysiol..

[73] Al-Timemy A.H., Khushaba R.N., Bugmann G., Escudero J. (2015). Improving the performance against force variation of EMG controlled multifunctional upper-limb prostheses for transradial amputees. IEEE Trans. Neural Syst. Rehabil. Eng..

[74] Phinyomark A., Khushaba R.N., Ibáñez-Marcelo E., Patania A., Scheme E., Petri G. (2017). Navigating features: A topologically informed chart of electromyographic features space. J. R. Soc. Interface.

[75] Oskoei M.A., Hu H. (2008). Support vector machine-based classification scheme for myoelectric control applied to upper limb. IEEE Trans. Biomed. Eng..

[76] Saponas T.S., Tan D.S., Morris D., Balakrishnan R. (2008). Demonstrating the feasibility of using forearm electromyography for muscle-computer interfaces. Proceedings of the SIGCHI Conference on Human Factors in Computing Systems.

[77] Phinyomark A., Phukpattaranont P., Limsakul C. (2012). Fractal analysis features for weak and single-channel upper-limb EMG signals. Expert Syst. Appl..

[78] Qingju Z., Zhizeng L. Wavelet de-noising of electromyography. Proceedings of the IEEE International Conference on Mechatronics and Automation.

[79] Du S., Vuskovic M. Temporal vs. spectral approach to feature extraction from prehensile EMG signals. Proceedings of the 2004 IEEE International Conference on Information Reuse and Integration.

[80] Sinderby C., Lindstrom L., Grassino A. (1995). Automatic assessment of electromyogram quality. J. Appl. Physiol..

[81] McCool P., Fraser G.D., Chan A.D., Petropoulakis L., Soraghan J.J. (2014). Identification of contaminant type in surface electromyography (EMG) signals. IEEE Trans. Neural Syst. Rehabil. Eng..

[82] Phinyomark A., Phothisonothai M., Phukpattaranont P., Limsakul C. (2011). Critical exponent analysis applied to surface EMG signals for gesture recognition. Metrol. Meas. Syst..

[83] Phinyomark A., Phothisonothai M., Phukpattaranont P., Limsakul C. (2011). Evaluation of movement types and electrode positions for EMG pattern classification based on linear and non-linear features. Eur. J. Sci. Res.

[84] Thongpanja S., Phinyomark A., Quaine F., Laurillau Y., Limsakul C., Phukpattaranont P. (2016). Probability density functions of stationary surface EMG signals in noisy environments. IEEE Trans. Instrum. Meas..

[85] Van Den Broek E.L., Schut M.H., Westerink J.H., van Herk J., Tuinenbreijer K. (2006). Computing emotion awareness through facial electromyography. Proceedings of the European Conference on Computer Vision.

[86] Talebinejad M., Chan A.D., Miri A., Dansereau R.M. (2009). Fractal analysis of surface electromyography signals: A novel power spectrum-based method. J. Electromyogr. Kinesiol..

[87] Oskoei M.A., Hu H. GA-based feature subset selection for myoelectric classification. Proceedings of the IEEE International Conference on Robotics and Biomimetics.

[88] Phinyomark A., Phukpattaranont P., Limsakul C. (2014). Applications of variance fractal dimension: A survey. Fractals.

[89] Khushaba R.N., Al-Ani A., Al-Timemy A., Al-Jumaily A. A fusion of time-domain descriptors for improved myoelectric hand control. Proceedings of the IEEE Symposium Series on Computational Intelligence (SSCI).

[90] He J., Zhang D., Sheng X., Meng J., Zhu X. Improved discrete fourier transform based spectral feature for surface electromyogram signal classification. Proceedings of the 35th Annual International Conference of the IEEE Engineering in Medicine and Biology Society (EMBC).

[91] Khushaba R.N., Krasoulis A., Al-Jumaily A., Nazarpour K. Spatio-Temporal Inertial Measurements Feature Extraction Improves Hand Movement Pattern Recognition without Electromyography. Proceedings of the 40th Annual International Conference of the IEEE Engineering in Medicine and Biology Society (EMBC).

[92] Hu X., Wang Z., Ren X. (2005). Classification of surface EMG signal using relative wavelet packet energy. Comput. Methods Programs Biomed..

[93] Chang G.C., Kang W.J., Luh J.J., Cheng C.K., Lai J.S., Chen J.J.J., Kuo T.S. (1996). Real-time implementation of electromyogram pattern recognition as a control command of man-machine interface. Med. Eng. Phys..

[94] Khushaba R.N., Shi L., Kodagoda S. Time-dependent spectral features for limb position invariant myoelectric pattern recognition. Proceedings of the 2012 International Symposium on Communications and Information Technologies (ISCIT).

[95] Tkach D., Huang H., Kuiken T.A. (2010). Study of stability of time-domain features for electromyographic pattern recognition. J. Neuroeng. Rehabil..

[96] He J., Zhang D., Sheng X., Li S., Zhu X. (2015). Invariant Surface EMG Feature Against Varying Contraction Level for Myoelectric Control Based on Muscle Coordination. IEEE J. Biomed. Health Inform..

[97] Liu J., Li X., Li G., Zhou P. (2014). EMG feature assessment for myoelectric pattern recognition and channel selection: A study with incomplete spinal cord injury. Med. Eng. Phys..

[98] Negi S., Kumar Y., Mishra V.M. Feature extraction and classification for EMG signals using linear discriminant analysis. Proceedings of the 2nd International Conference on Advances in Computing, Communication, Automation (ICACCA) (Fall).

[99] Ververidis D., Kotropoulos C. Sequential forward feature selection with low computational cost. Proceedings of the 13th European Signal Processing Conference.

[100] Gao Q., Wang Q., Huang Y., Gao X., Hong X., Zhang H. (2015). Dimensionality reduction by integrating sparse representation and Fisher criterion and its applications. IEEE Trans. Image Process..

[101] Lee D.D., Seung H.S. (2001). Algorithms for non-negative matrix factorization. Advances in Neural Information Processing Systems.

[102] Thompson B. (2005). Canonical correlation analysis. Encyclopedia of Statistics in Behavioral Science.

[103] Güler N.F., Koçer S. (2005). Classification of EMG signals using PCA and FFT. J. Med. Syst..

[104] Balasubramanian M., Schwartz E.L. (2002). The isomap algorithm and topological stability. Science.

[105] Maaten L.v.d., Hinton G. (2008). Visualizing data using t-SNE. J. Mach. Learn. Res..

[106] Hinton G.E., Zemel R.S. (1994). Autoencoders, minimum description length and Helmholtz free energy. Advances in Neural Information Processing Systems.

[107] Kruskal J.B. (1964). Nonmetric multidimensional scaling: A numerical method. Psychometrika.

[108] Côté-Allard U., Campbell E., Phinyomark A., Laviolette F., Gosselin B., Scheme E. (2019). Interpreting deep learning features for myoelectric control: A comparison with handcrafted features. arXiv.

[109] Xia P., Hu J., Peng Y. (2018). EMG-based estimation of limb movement using deep learning with recurrent convolutional neural networks. Artif. Organs.

[110] Scheme E.J., Englehart K.B. (2012). Validation of a selective ensemble-based classification scheme for myoelectric control using a three-dimensional Fitts’ law test. IEEE Trans. Neural Syst. Rehabil. Eng..

[111] Kamavuako E.N., Scheme E.J., Englehart K.B. (2014). On the usability of intramuscular EMG for prosthetic control: A Fitts’ law approach. J. Electromyogr. Kinesiol..

[112] Wurth S.M., Hargrove L.J. (2014). A real-time comparison between direct control, sequential pattern recognition control and simultaneous pattern recognition control using a Fitts’ law style assessment procedure. J. Neuroeng. Rehabil..

[113] Scheme E., Fougner A., Stavdahl Ø., Chan A.D.C., Englehart K. Examining the adverse effects of limb position on pattern recognition based myoelectric control. Proceedings of the 2010 Annual International Conference of the IEEE Engineering in Medicine and Biology.

[114] Jiang N., Muceli S., Graimann B., Farina D. (2013). Effect of arm position on the prediction of kinematics from EMG in amputees. Med. Biol. Eng. Comput..

[115] Chen L., Geng Y., Li G. Effect of upper-limb positions on motion pattern recognition using electromyography. Proceedings of the 4th International Congress on Image and Signal Processing.

[116] Liu J., Zhang D., He J., Zhu X. Effect of dynamic change of arm position on myoelectric pattern recognition. Proceedings of the IEEE International Conference on Robotics and Biomimetics (ROBIO).

[117] Clites T., Herr H., Srinivasan S., Zorzos A., Carty M. (2018). The Ewing Amputation: The First Human Implementation of the Agonist-Antagonist Myoneural Interface. Plast. Reconstr. Surg..

[118] Lorrain T., Jiang N., Farina D. (2011). Influence of the training set on the accuracy of surface EMG classification in dynamic contractions for the control of multifunction prostheses. J. Neuroeng. Rehabil..

[119] You K.J., Rhee K.W., Shin H.C. (2010). Finger Motion Decoding Using EMG Signals Corresponding Various Arm Postures. Exp Neurobiol.

[120] Khushaba R.N., Al-Timemy A., Kodagoda S. Influence of multiple dynamic factors on the performance of myoelectric pattern recognition. Proceedings of the 37th Annual International Conference of the IEEE Engineering in Medicine and Biology Society (EMBC).

[121] Ishii A., Kondo T., Yano S., Chen W., Hosoda K., Menegatti E., Shimizu M., Wang H. (2017). Improvement of EMG Pattern Recognition by Eliminating Posture-Dependent Components. Intelligent Autonomous Systems 14.

[122] Yang D., Yang W., Huang Q., Liu H. (2017). Classification of Multiple Finger Motions During Dynamic Upper Limb Movements. IEEE J. Biomed. Health Inform..

[123] Yang D., Gu Y., Jiang L., Osborn L., Liu H. (2017). Dynamic training protocol improves the robustness of PR-based myoelectric control. Biomed. Signal Process. Control.

[124] Adewuyi A.A., Hargrove L.J., Kuiken T.A. (2017). Resolving the effect of wrist position on myoelectric pattern recognition control. J. Neuroeng. Rehabil..

[125] Gupta T., Yadav J., Chaudhary S., Agarwal U., Thampi S.M., Mitra S., Mukhopadhyay J., Li K.C., James A.P., Berretti S. (2018). EMG Pattern Classification Using Neural Networks. Intelligent Systems Technologies and Applications.

[126] Geng Y., Chen L., Tian L., Li G. Comparison of electromyography and mechanomyogram in control of prosthetic system in multiple limb positions. Proceedings of the IEEE-EMBS International Conference on Biomedical and Health Informatics.

[127] Geng Y., Zhou P., Li G. (2012). Toward attenuating the impact of arm positions on electromyography pattern-recognition based motion classification in transradial amputees. J. NeuroEng. Rehabil..

[128] Geng Y., Zhang F., Yang L., Zhang Y., Li G. Reduction of the effect of arm position variation on real-time performance of motion classification. Proceedings of the Annual International Conference of the IEEE Engineering in Medicine and Biology Society.

[129] Khushaba R.N. (2014). Correlation Analysis of Electromyogram Signals for Multiuser Myoelectric Interfaces. IEEE Trans. Neural Syst. Rehabil. Eng..

[130] Liu J., Zhang D., Sheng X., Zhu X. (2014). Quantification and solutions of arm movements effect on sEMG pattern recognition. Biomed. Signal Process. Control.

[131] Masters M.R., Smith R.J., Soares A.B., Thakor N.V. Towards better understanding and reducing the effect of limb position on myoelectric upper-limb prostheses. Proceedings of the 36th Annual International Conference of the IEEE Engineering in Medicine and Biology Society.

[132] Radmand A., Scheme E., Englehart K. (2014). On the Suitability of Integrating Accelerometry Data with Electromyography Signals for Resolving the Effect of Changes in Limb Position during Dynamic Limb Movement. J. Prosthet. Orthot..

[133] Boschmann A., Platzner M. Towards robust HD EMG pattern recognition: Reducing electrode displacement effect using structural similarity. Proceedings of the 36th Annual International Conference of the IEEE Engineering in Medicine and Biology Society, EMBC.

[134] Al-Angari H.M., Kanitz G., Tarantino S., Cipriani C. (2016). Distance and mutual information methods for EMG feature and channel subset selection for classification of hand movements. Biomed. Signal Process. Control.

[135] Betthauser J.L., Hunt C.L., Osborn L.E., Kaliki R.R., Thakor N.V. Limb-position robust classification of myoelectric signals for prosthesis control using sparse representations. Proceedings of the 38th Annual International Conference of the IEEE Engineering in Medicine and Biology Society (EMBC).

[136] Yu Y., Sheng X., Guo W., Zhu X. Attenuating the impact of limb position on surface EMG pattern recognition using a mixed-LDA classifier. Proceedings of the 2017 IEEE International Conference on Robotics and Biomimetics (ROBIO).

[137] Yanjuan G., Oluwarotimi S.W., Yue W., Guanglin L. (2017). Improving the Robustness of Real-Time Myoelectric Pattern Recognition against Arm Position Changes in Transradial Amputees. BioMed Res. Int..

[138] Kanitz G., Cipriani C., Edin B.B. (2018). Classification of Transient Myoelectric Signals for the Control of Multi-Grasp Hand Prostheses. IEEE Trans. Neural Syst. Rehabil. Eng..

[139] Beaulieu R.J., Masters M.R., Betthauser J., Smith R.J., Kaliki R., Thakor N.V., Soares A.B. (2019). Multi-position training improves robustness of pattern recognition and reduces limb-position effect in prosthetic control. J. Prosthet. Orthot..

[140] Gu Y., Yang D., Huang Q., Yang W., Liu H. (2018). Robust EMG pattern recognition in the presence of confounding factors: Features, classifiers and adaptive learning. Expert Syst. Appl..

[141] Krasoulis A., Kyranou I., Erden M.S., Nazarpour K., Vijayakumar S. (2017). Improved prosthetic hand control with concurrent use of myoelectric and inertial measurements. J. Neuroeng. Rehabil..

[142] Urra O., Casals A., Jané R., Roa Romero L.M. (2014). Evaluating Spatial Characteristics of Upper-Limb Movements from EMG Signals. Proceedings of the XIII Mediterranean Conference on Medical and Biological Engineering and Computing.

[143] González J., Horiuchi Y., Yu W. (2010). Classification of Upper Limb Motions from Around-Shoulder Muscle Activities: Hand Biofeedback. Open Med. Informatics J..

[144] Horiuchi Y., Kishi T., Gonzalez J., Yu W. A study on classification of upper limb motions from around-shoulder muscle activities. Proceedings of the IEEE International Conference on Rehabilitation Robotics.

[145] Liarokapis M.V., Artemiadis P.K., Katsiaris P.T., Kyriakopoulos K.J., Manolakos E.S. Learning human reach-to-grasp strategies: Towards EMG-based control of robotic arm-hand systems. Proceedings of the IEEE International Conference on Robotics and Automation.

[146] Liarokapis M., Artemiadis P., Katsiaris P., Kyriakopoulos K. Learning Task-Specific Models for Reach to Grasp Movements: Towards EMG-based Teleoperation of Robotic Arm-Hand Systems. Proceedings of the 4th IEEE RAS & EMBS International Conference on Biomedical Robotics and Biomechatronics (BioRob).

[147] Rivela D., Scannella A., Pavan E.E., Frigo C.A., Belluco P., Gini G. Processing of surface EMG through pattern recognition techniques aimed at classifying shoulder joint movements. Proceedings of the 37th Annual International Conference of the IEEE Engineering in Medicine and Biology Society (EMBC).

[148] Batzianoulis I., Krausz N.E., Simon A.M., Hargrove L., Billard A. (2018). Decoding the grasping intention from electromyography during reaching motions. J. Neuroeng. Rehabil..

[149] Schwarz A., Ofner P., Pereira J., Sburlea A.I., Mueller-Putz G.R. (2017). Decoding natural reach-and-grasp actions from human EEG. J. Neural Eng..

[150] Betthauser J.L., Hunt C.L., Osborn L.E., Masters M.R., Lévay G., Kaliki R.R., Thakor N.V. (2018). Limb Position Tolerant Pattern Recognition for Myoelectric Prosthesis Control with Adaptive Sparse Representations From Extreme Learning. IEEE Trans. Biomed. Eng..

[151] Cheng J., Wei F., Li C., Liu Y., Liu A., Chen X. (2018). Position-independent gesture recognition using sEMG signals via canonical correlation analysis. Comput. Biol. Med..

[152] Khushaba R.N., Takruri M., Miro J.V., Kodagoda S. (2014). Towards limb position invariant myoelectric pattern recognition using time-dependent spectral features. Neural Networks.

[153] Mukhopadhyay A.K., Samui S. (2020). An experimental study on upper limb position invariant EMG signal classification based on deep neural network. Biomed. Signal Process. Control.

[154] Powar O.S., Chemmangat K. (2020). Reducing the effect of wrist variation on pattern recognition of Myoelectric Hand Prostheses Control through Dynamic Time Warping. Biomed. Signal Process. Control.

[155] Liu J., Ren Y., Xu D., Kang S.H., Zhang L. (2019). EMG-Based Real-Time Linear-Nonlinear Cascade Regression Decoding of Shoulder, Elbow and Wrist Movements in Able-Bodied Persons and Stroke Survivors. IEEE Trans. Biomed. Eng..

[156] Scheme E., Biron K., Englehart K. Improving myoelectric pattern recognition positional robustness using advanced training protocols. Proceedings of the Annual International Conference of the IEEE Engineering in Medicine and Biology Society.

[157] Gazzoni M., Celadon N., Mastrapasqua D., Paleari M., Margaria V., Ariano P. (2014). Quantifying Forearm Muscle Activity during Wrist and Finger Movements by Means of Multi-Channel Electromyography. PLoS ONE.

[158] Matsubara T., Morimoto J. (2013). Bilinear Modeling of EMG Signals to Extract User-Independent Features for Multiuser Myoelectric Interface. IEEE Trans. Biomed. Eng..

[159] Côté-Allard U., Fall C.L., Campeau-Lecours A., Gosselin C., Laviolette F., Gosselin B. Transfer learning for sEMG hand gestures recognition using convolutional neural networks. Proceedings of the IEEE International Conference on Systems, Man, and Cybernetics (SMC).

[160] Côé-Allard U., Latyr Fall C., Drouin A., Campeau-Lecours A., Gosselin C., Glette K., Laviolette F., Gosselin B. (2019). Deep Learning for Electromyographic Hand GestureSignal Classification Using Transfer Learning. arXiv.

[161] Du Y., Jin W., Wei W., Hu Y., Geng W. (2017). Surface EMG-Based Inter-Session Gesture Recognition Enhanced by Deep Domain Adaptation. Sensors.

[162] Shahzad W., Ayaz Y., Khan M.J., Naseer N., Khan M. (2019). Enhanced Performance for Multi-Forearm Movement Decoding Using Hybrid IMU–sEMG Interface. Front. Neurorobotics.

[163] Campbell E., Phinyomark A., Scheme E. (2020). Differences in Perspective on Inertial Measurement Unit Sensor Integration in Myoelectric Control. arXiv.

[164] Buskirk E., Komi P. (1970). Reproducibility of electromyographic measurements with inserted wire electrodes and surface electrodes. Acta Physiol. Scand..

[165] Moritani T., Devries H.A. (1979). Neural factors versus hypertrophy in the time course of muscle strength gain. Am. J. Phys. Med..

[166] Moritani T., Devries H.A. (1978). Reexamination of the relationship between the surface integrated electromyogram (IEMG) and force of isometric contraction. Am. J. Phys. Med..

[167] Moritani T., Muro M. (1987). Motor unit activity and surface electromyogram power spectrum during increasing force of contraction. Eur. J. Appl. Physiol. Occup. Physiol..

[168] Scheme E., Lock B., Hargrove L., Hill W., Kuruganti U., Englehart K. (2014). Motion Normalized Proportional Control for Improved Pattern Recognition-Based Myoelectric Control. IEEE Trans. Neural Syst. Rehabil. Eng..

[169] Yatsenko D., McDonnall D., Guillory K.S. Simultaneous, proportional, multi-axis prosthesis control using multichannel surface EMG. Proceedings of the 29th Annual International Conference of the IEEE Engineering in Medicine and Biology Society.

[170] Rehbaum H., Jiang N., Paredes L., Amsuess S., Graimann B., Farina D. Real time simultaneous and proportional control of multiple degrees of freedom from surface EMG: Preliminary results on subjects with limb deficiency. Proceedings of the IEEE Annual International Conference of the IEEE Engineering in Medicine and Biology Society.

[171] Harris C.M., Wolpert D.M. (1998). Signal-dependent noise determines motor planning. Nature.

[172] Jones K.E., Hamilton A.F.d.C., Wolpert D.M. (2002). Sources of signal-dependent noise during isometric force production. J. Neurophysiol..

[173] Al-Timemy A.H., Bugmann G., Escudero J., Outram N. A preliminary investigation of the effect of force variation for myoelectric control of hand prosthesis. Proceedings of the 35th Annual International Conference of the IEEE Engineering in Medicine and Biology Society (EMBC).

[174] Brucker B.S., Buylaeva N.V. (1996). Biofeedback effect on electromyography responses in patients with spinal cord injury. Arch. Phys. Med. Rehabil..

[175] Halaki M., Ginn K. (2012). Normalization of EMG signals: To normalize or not to normalize and what to normalize to?. Computational Intelligence in Electromyography Analysis—A Perspective on Current Applications and Future Challenges.

[176] Perry J., Davids J.R., Burnfield J.M. (1992). Gait analysis: Normal and pathological function. J. Pediatr. Orthop..

[177] Winter D. (2017). EMG interpretation. Electromyography in Ergonomics.

[178] Jobe F.W., Moynes D.R., Tibone J.E., Perry J. (1984). An EMG analysis of the shoulder in pitching: A second report. Am. J. Sport. Med..

[179] Pincivero D.M., Salfetnikov Y., Campy R.M., Coelho A.J. (2004). Angle-and gender-specific quadriceps femoris muscle recruitment and knee extensor torque. J. Biomech..

[180] Mathiassen S., Winkel J., Hägg G. (1995). Normalization of surface EMG amplitude from the upper trapezius muscle in ergonomic studies—A review. J. Electromyogr. Kinesiol..

[181] De Luca C.J. (1997). The use of surface electromyography in biomechanics. J. Appl. Biomech..

[182] Piitulainen H., Rantalainen T., Linnamo V., Komi P., Avela J. (2009). Innervation zone shift at different levels of isometric contraction in the biceps brachii muscle. J. Electromyogr. Kinesiol..

[183] Gander R. (1985). Power spectral density of the surface myoelectric signal of the biceps brachi as a function of static load. Electromyograph. Clin. Neurophysiol..

[184] Gerdle B., Eriksson N., Brundin L. (1990). The behaviour of the mean power frequency of the surface electromyogram in biceps brachii with increasing force and during fatigue. With special regard to the electrode distance. Electromyogr. Clin. Neurophysiol..

[185] Li W., Sakamoto K. (1996). The influence of location of electrode on muscle fiber conduction velocity and EMG power spectrum during voluntary isometric contraction measured with surface array electrodes. Appl. Hum. Sci..

[186] Rainoldi A., Galardi G., Maderna L., Comi G., Conte L.L., Merletti R. (1999). Repeatability of surface EMG variables during voluntary isometric contractions of the biceps brachii muscle. J. Electromyogr. Kinesiol..

[187] Kaplanis P., Pattichis C., Hadjileontiadis L., Roberts V. (2009). Surface EMG analysis on normal subjects based on isometric voluntary contraction. J. Electromyogr. Kinesiol..

[188] Hussain M., Reaz M.B.I., Mohd-Yasin F., Ibrahimy M.I. (2009). Electromyography signal analysis using wavelet transform and higher order statistics to determine muscle contraction. Expert Syst..

[189] Nazarpour K., Al-Timemy A.H., Bugmann G., Jackson A. (2013). A note on the probability distribution function of the surface electromyogram signal. Brain Res. Bull..

[190] Naik G.R., Kumar D.K., Arjunan S.P. Kurtosis and negentropy investigation of myo electric signals during different MVCs. Proceedings of the ISSNIP Biosignals and Biorobotics Conference 2011.

[191] Kaplanis P., Pattichis C., Hadjileontiadis L., Panas S. Bispectral analysis of surface EMG. Proceedings of the 10th IEEE Mediterranean Electrotechnical Conference. Information Technology and Electrotechnology for the Mediterranean Countries, MeleCon 2000 (Cat. No. 00CH37099).

[192] Li X., Fang P., Tian L., Li G. Increasing the robustness against force variation in EMG motion classification by common spatial patterns. Proceedings of the 2017 39th Annual International Conference of the IEEE Engineering in Medicine and Biology Society (EMBC).

[193] Ameri A., Kamavuako E.N., Scheme E.J., Englehart K.B., Parker P.A. (2014). Real-time, simultaneous myoelectric control using visual target-based training paradigm. Biomed. Signal Process. Control.

[194] Pan L., Zhang D., Liu J., Sheng X., Zhu X. (2014). Continuous estimation of finger joint angles under different static wrist motions from surface EMG signals. Biomed. Signal Process. Control.

[195] Iqbal N.V., Subramaniam K., P. S.A. (2019). Robust feature sets for contraction level invariant control of upper limb myoelectric prosthesis. Biomed. Signal Process. Control.

[196] Iqbal N.V., Subramaniam K., P. S.A. (2018). Wavelet Packet Entropy Based Control of Myoelectric Prosthesis. Biomed. Pharmacol. J..

[197] Lv B., Sheng X., Guo W., Zhu X., Ding H., Huang Y., Wu H., Liu H., Yin Z. (2017). Towards Finger Gestures and Force Recognition Based on Wrist Electromyography and Accelerometers. Intelligent Robotics and Applications.

[198] Atoufi B., Kamavuako E., Hudgins B., Englehart K. (2015). Classification of hand and wrist tasks of unknown force levels using muscle synergies. Proceedings of the 37th Annual International Conference of the IEEE Engineering in Medicine and Biology Society, EMBC.

[199] Cai D., He X., Han J. (2008). SRDA: An Efficient Algorithm for Large-Scale Discriminant Analysis. IEEE Trans. Knowl. Data Eng..

[200] Powar O.S., Chemmangat K. (2019). Dynamic time warping for reducing the effect of force variation on myoelectric control of hand prostheses. J. Electromyogr. Kinesiol..

[201] Li X., Xu R., Samuel O.W., Tian L., Zou H., Zhang X., Chen S., Fang P., Li G. A new approach to mitigate the effect of force variation on pattern recognition for myoelectric control. Proceedings of the 38th Annual International Conference of the IEEE Engineering in Medicine and Biology Society (EMBC).

[202] Hoozemans M.J., van Dieën J.H. (2005). Prediction of handgrip forces using surface EMG of forearm muscles. J. Electromyogr. Kinesiol..

[203] Atoufi B., Kamavuako E., Hudgins B., Englehart K. (2014). Toward proportional control of myoelectric prostheses with muscle synergies. J. Med. Biol. Eng..

[204] Bilodeau M., Schindler-Ivens S., Williams D., Chandran R., Sharma S. (2003). EMG frequency content changes with increasing force and during fatigue in the quadriceps femoris muscle of men and women. J. Electromyogr. Kinesiol..

[205] Karlsson S., Gerdle B. (2001). Mean frequency and signal amplitude of the surface EMG of the quadriceps muscles increase with increasing torque—A study using the continuous wavelet transform. J. Electromyogr. Kinesiol..

[206] Soma H., Horiuchi Y., Gonzalez J., Yu W., Mizrahi J. (2011). Classification of Upper Limb Motions from Around-Shoulder Muscle Activities. Advances in Applied Electromyography.

[207] Luciw M., Jarocka E., Edin B. (2014). Multi-channel EEG recordings during 3936 grasp and lift trials with varying weight and friction. Sci. Data.

[208] Amsüss S., Paredes L.P., Rudigkeit N., Graimann B., Herrmann M.J., Farina D. Long term stability of surface EMG pattern classification for prosthetic control. Proceedings of the 2013 35th Annual International Conference of the IEEE Engineering in Medicine and Biology Society (EMBC).

