# Supplementary material for: Current Trends and Confounding Factors in Myoelectric Control: Limb Position and Contraction Intensity
Source: Sensors (Basel). 2020 Mar 13;20(6):1613. doi: 10.3390/s20061613 (PMC7146367; doi:10.3390/s20061613)
Supplement: Supplementary file 1 [file sensors-20-01613-s001.zip › sensors-725895 - SI/Figures/EMGPR_pipeline.pdf]

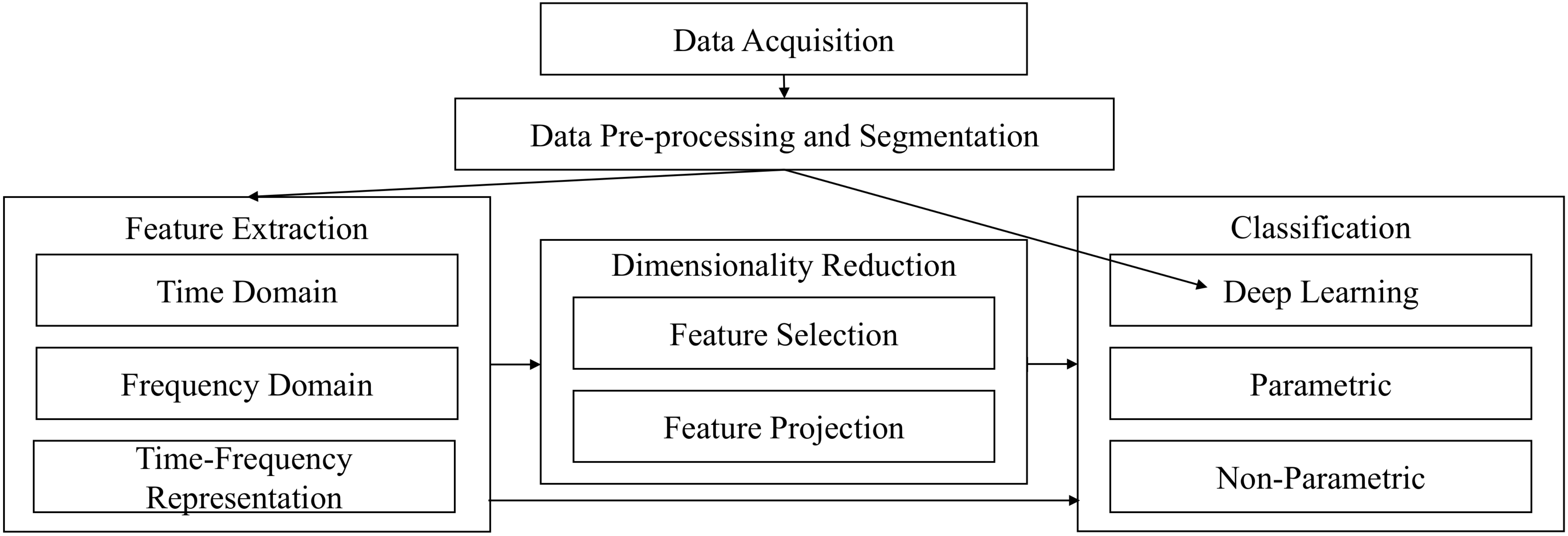
Data Acquisition
Data Pre-processing and Segmentation
Feature Extraction
Time Domain
Frequency Domain
Time-Frequency Representation
Dimensionality Reduction
Feature Selection
Feature Projection
Classification
Deep Learning
Parametric
Non-Parametric
