# Supplementary material for: Current Trends and Confounding Factors in Myoelectric Control: Limb Position and Contraction Intensity
Source: Sensors (Basel). 2020 Mar 13;20(6):1613. doi: 10.3390/s20061613 (PMC7146367; doi:10.3390/s20061613)
Supplement: Supplementary file 1 [file sensors-20-01613-s001.zip › sensors-725895 - SI/Figures/ValidationScheme.pdf]

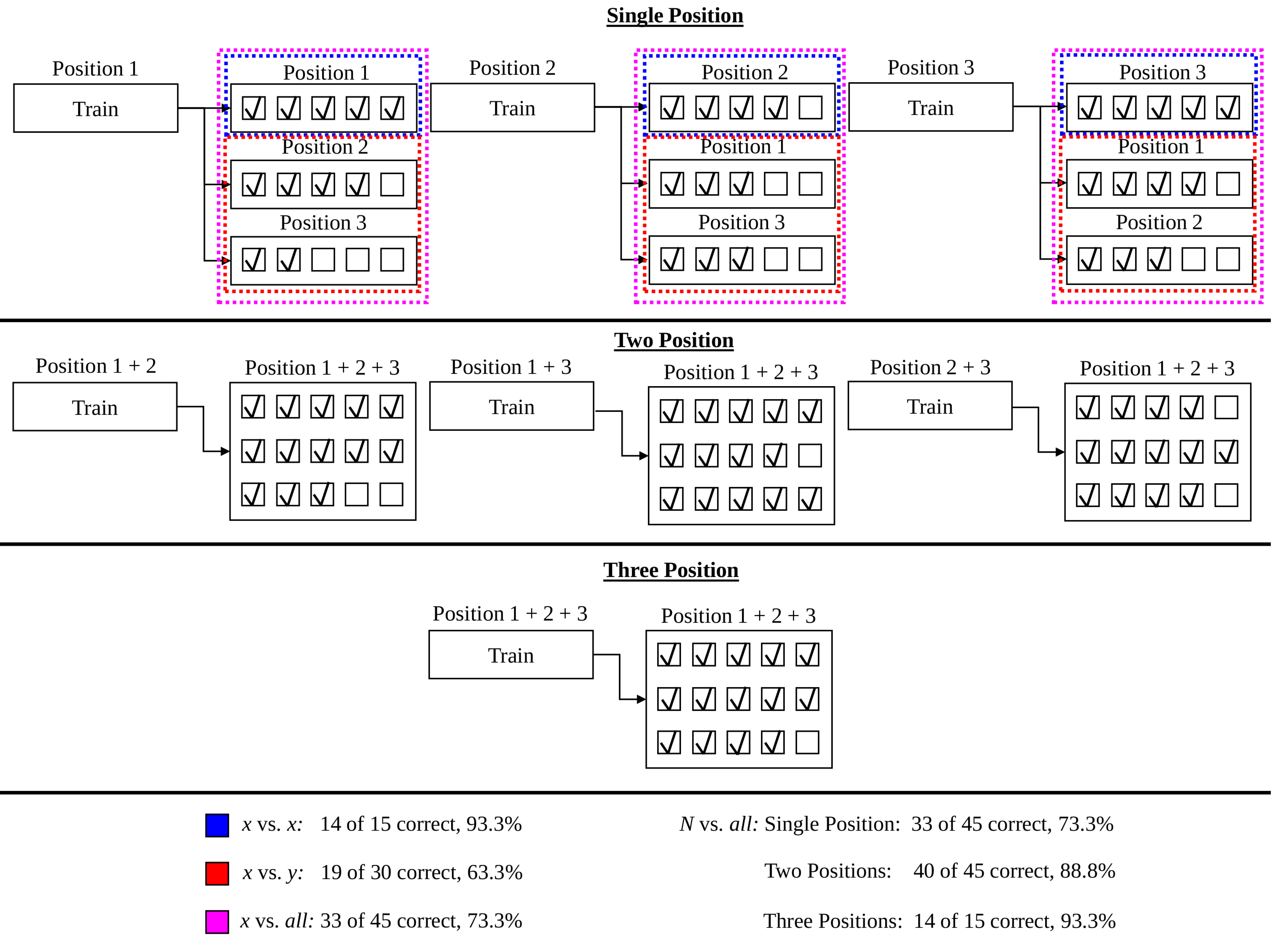
Single Position
Position 1
Train
Position 1
Position 2
Position 3
Position 2
Train
Position 2
Position 1
Position 3
Position 3
Train
Position 3
Position 1
Position 2
Two Position
Position 1 + 2
Train
Position 1 + 2 + 3
Position 1 + 3
Train
Position 1 + 2 + 3
Position 2 + 3
Train
Position 1 + 2 + 3
Three Position
Position 1 + 2 + 3
Train
Position 1 + 2 + 3
x vs. x: 14 of 15 correct, 93.3%
x vs. y: 19 of 30 correct, 63.3%
x vs. all: 33 of 45 correct, 73.3%
N vs. all: Single Position: 33 of 45 correct, 73.3%
Two Positions: 40 of 45 correct, 88.8%
Three Positions: 14 of 15 correct, 93.3%
