# Supplementary material for: Current Trends and Confounding Factors in Myoelectric Control: Limb Position and Contraction Intensity
Source: Sensors (Basel). 2020 Mar 13;20(6):1613. doi: 10.3390/s20061613 (PMC7146367; doi:10.3390/s20061613)
Supplement: Supplementary file 1 [file sensors-20-01613-s001.zip › sensors-725895 - SI/SupplementaryMaterials.pdf]

sensors

MDPI

Article

Current Trends and Confounding Factors in Myoelectric Control: Limb Position and Contraction Intensity

Evan Campbell [1,2], Angkoon Phinyomark [2] and Erik Scheme [1,2,*]

1 Department of Electrical and Computer Engineering, University of New Brunswick, FR; Evan.Campbell1@unb.ca (E.C.); aphinyom@unb.ca(A.P.)
2 Institute of Biomedical Engineering, University of New Brunswick, FR
* Correspondence: escheme@unb.ca; Tel.: +1-506-453-4966

Received: 5 February 2020; Accepted: 9 March 2020; Published: date

check for updates

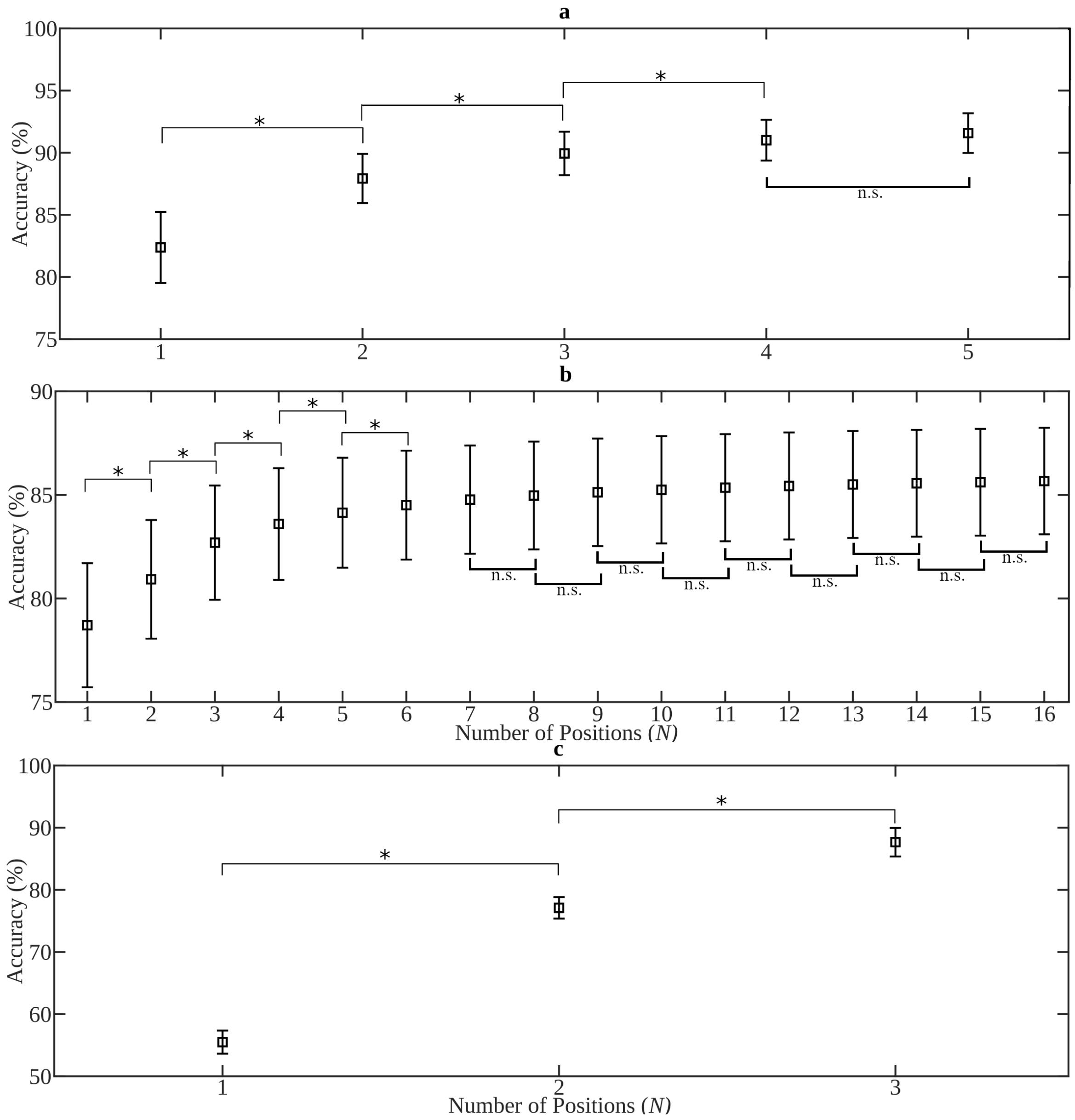

Figure S1. Results of *N* vs. all testing regime for the position factor using the TD feature set and the LDA classifier. Classification accuracy was plotted with error bars indicating standard error measurements. * indicated significant different with prior *N*, whereas n.s. indicates no significant difference. a) 5 static limb-position dataset. b) 16 static limb-position dataset. c) 3 static forearm-orientation dataset.

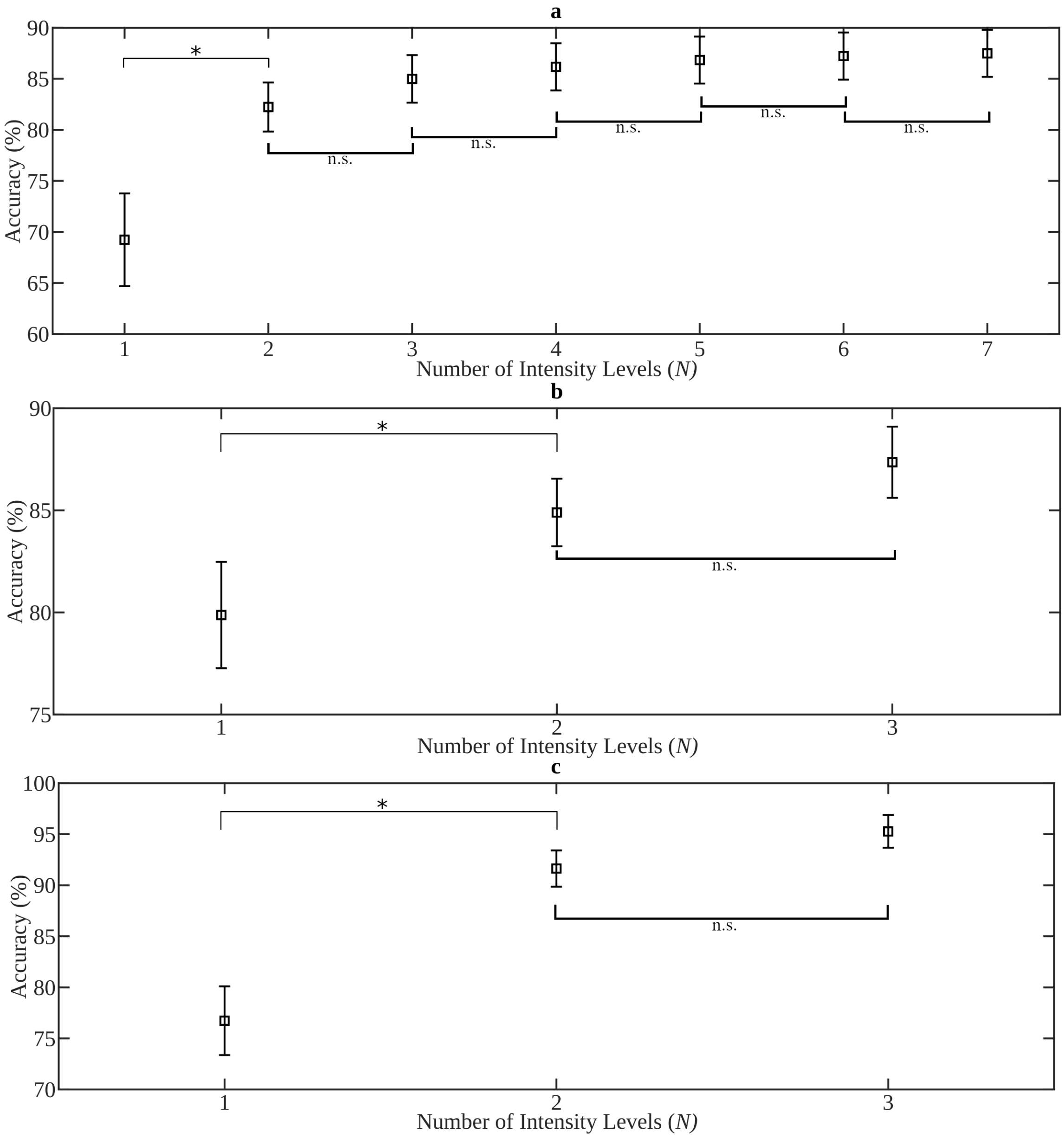

Figure S2. Results of *N* vs. all testing regime for the contraction intensity factor using the TD feature set and the LDA classifier. Classification accuracy was plotted with error bars indicating standard error measurements. * indicated significant different with prior *N*, whereas n.s. indicates no significant difference. a) 7 %MVC level dataset. b) 3 subjective levels (A) dataset. c) 3 subjective levels (B) dataset.

Feature Set	Classifier	Test Regime	5 Limb Position Mean±SD (Min, Max)	16 Limb Position Mean±SD (Min, Max)	3 Forearm Orientation Mean±SD (Min, Max)
TD	LDA	*Intra-Position*	94.5±1.2 (93.1, 96)	86.9±1.9 (83.7, 90.2)	96.1±1.4 (94.6, 97.3)
		Inter-Position	80.2±7.1 (67.7, 92)	75.4±6.6 (58.2, 89.4)	36.8±8.6 (27.8, 47.4)
		Single-Position	83.1±2.2 (80.6, 86.6)	76.1±2.9 (70.9, 80.1)	56.6±1.1 (55.5, 57.6)
	QDA	*Intra-Position*	94.3±0.7 (93.6, 95.1)	85.2±1.7 (82.4, 88.8)	94.2±1.8 (92.8, 96.2)
		Inter-Position	81.1±7 (69.7, 91.8)	71±7.9 (51.3, 86.5)	28.1±4.9 (20.8, 35)
		Single-Position	83.8±1.3 (81.8, 85.1)	71.9±1.7 (68, 74.3)	50.1±0.7 (49.5, 50.9)
	*k*NN	*Intra-Position*	94±1.1 (92.2, 94.9)	62.9±1.9 (60.4, 66.5)	86.1±3.8 (83.5, 90.4)
		Inter-Position	84.2±4.4 (76.2, 91.5)	52.7±5.4 (41.6, 64.7)	35±7.8 (27.9, 45.9)
		Single-Position	86.1±1 (85.4, 87.7)	53.4±2 (50.5, 56.2)	52±1.7 (50.6, 53.9)
	RF	*Intra-Position*	94±0.8 (92.8, 94.9)	85.8±2.2 (81.9, 89)	94±1.9 (92.3, 96)
		Inter-Position	83.3±5.2 (74.3, 90.9)	74±6.6 (53.9, 88.2)	38.5±6.7 (29.5, 45)
		Single-Position	85.4±1.2 (84, 87.2)	74.8±2.5 (71.2, 78.6)	56.6±2 (55.5, 58.9)
	SVM	*Intra-Position*	95.2±0.9 (93.7, 96.1)	85.9±1.8 (83, 88.8)	92.3±1.7 (90.7, 94.1)
		Inter-Position	86.5±4.7 (77.5, 93.8)	74.2±6.4 (57.2, 86.4)	36.1±8.8 (25.7, 47.6)
		Single-Position	88.3±0.8 (86.9, 89.1)	74.9±2.6 (70.3, 78.4)	54.8±2.2 (53.1, 57.3)
LSF4	LDA	*Intra-Position*	95.9±0.7 (95.2, 96.9)	90.5±1.7 (87.5, 93.4)	96.9±1.6 (95.1, 98.2)
		Inter-Position	84.2±6.4 (73.1, 94.6)	79.5±6.9 (58.1, 91.8)	31.9±6.7 (25.3, 41.9)
		Single-Position	86.5±1.3 (85.2, 88.5)	80.2±2.6 (75.7, 83)	53.6±1 (52.5, 54.1)
	QDA	*Intra-Position*	94.9±0.7 (94.1, 95.8)	87.8±2.2 (84.5, 91.6)	95.6±1.6 (94.1, 97.2)
		Inter-Position	84.7±5.6 (76.4, 93.6)	75.7±7.3 (55.8, 90.6)	28±5.5 (20.2, 35.1)
		Single-Position	86.8±0.9 (85.5, 88)	76.5±1.4 (74.1, 78.7)	50.5±1.2 (49.8, 51.9)
	*k*NN	*Intra-Position*	89.7±1.5 (87.8, 91.6)	82.9±2.4 (78.3, 86.8)	91.7±1.1 (90.8, 92.9)
		Inter-Position	78.6±4.8 (69.5, 85.8)	71.3±6.9 (53.3, 84.5)	46.6±7.6 (36.5, 55.8)
		Single-Position	80.8±1.1 (79, 82.1)	72±2.2 (67.9, 74.9)	61.6±1.5 (60.1, 63.1)
	RF	*Intra-Position*	93.6±0.8 (92.4, 94.7)	84.8±2.2 (81, 87.8)	94±2.5 (91.8, 96.7)
		Inter-Position	82.3±5.3 (73.2, 89.7)	73±6.7 (53.1, 86.3)	37.7±6.1 (30, 44.6)
		Single-Position	84.6±1.2 (83.2, 86.4)	73.8±2.6 (69.2, 77.4)	56.2±1.6 (55, 58.1)
	SVM	*Intra-Position*	93.3±0.8 (92.4, 94.4)	87.5±2.1 (83.4, 90.9)	91.8±1.4 (90.9, 93.3)
		Inter-Position	82.7±5.5 (73.4, 90.9)	76.1±6.6 (57.8, 89.3)	47.6±7 (37.8, 56.2)
		Single-Position	84.8±0.8 (83.7, 85.6)	76.8±2.3 (72.9, 80)	62.3±1 (61.2, 63.2)
LSF9	LDA	*Intra-Position*	96.8±0.6 (96, 97.4)	90.6±1.7 (87.7, 93.6)	97.4±1.2 (96, 98.1)
		Inter-Position	86±6 (75.9, 95.6)	78.9±7.1 (58, 92.3)	30±7.1 (23.8, 41)
		Single-Position	88.1±1.3 (86.4, 89.9)	79.7±2.4 (75.7, 82.5)	52.5±2.3 (49.9, 54.5)
	QDA	*Intra-Position*	93.8±0.7 (93.1, 94.6)	85.2±2.4 (81.3, 89.5)	92.6±2.8 (90, 95.5)
		Inter-Position	81.7±6.5 (70.7, 92.1)	71.7±8 (49.5, 87.2)	26.6±4.1 (20.4, 31.4)
		Single-Position	84.1±1 (82.9, 85.5)	72.5±1.3 (70.6, 74.7)	48.6±1.2 (47.2, 49.4)
	*k*NN	*Intra-Position*	94.4±0.6 (93.4, 94.9)	60±2.1 (57.8, 63.5)	93.5±1.4 (92.4, 95)
		Inter-Position	85.2±4.1 (77.4, 91)	51±4.6 (41.1, 60.6)	45.7±7.7 (36, 54.6)
		Single-Position	87±0.8 (86, 88.2)	51.5±1.7 (49, 53.8)	61.6±1.7 (59.8, 63)
	RF	*Intra-Position*	93.7±0.7 (92.7, 94.4)	85±2.3 (81.2, 88.9)	93.7±2.3 (91.4, 96)
		Inter-Position	82.5±5.1 (73.7, 90.2)	72.5±6.9 (50.9, 87.4)	37.5±5.1 (30, 41.8)
		Single-Position	84.6±1.3 (83.2, 86.7)	73.3±2.8 (68.9, 77.4)	55.2±1.3 (53.9, 56.5)
	SVM	*Intra-Position*	95.6±0.7 (94.5, 96.3)	87.8±1.9 (84, 90.6)	94.5±1.6 (93.2, 96.2)
		Inter-Position	87.5±4.3 (79.2, 93.9)	77±6.3 (60.2, 88.8)	45.8±7.4 (35.5, 54.6)
		Single-Position	89.1±0.8 (87.9, 89.9)	77.7±2.7 (72.9, 80.9)	62±1.2 (60.8, 63.1)
TDPSD	LDA	*Intra-Position*	94.8±0.5 (94.1, 95.5)	90.1±1.9 (87.6, 93.5)	96±1.4 (94.6, 97.3)
		Inter-Position	82.6±6.8 (68, 93.1)	78.8±7 (57.7, 91.9)	38.8±7.5 (30.7, 51.1)
		Single-Position	85.1±1.7 (82.7, 87.3)	79.5±2.8 (74.5, 82.8)	57.9±1 (56.7, 58.8)
	QDA	*Intra-Position*	93.1±0.9 (92.2, 94.4)	87.3±2 (83.6, 90.8)	93.7±1.5 (92.4, 95.3)
		Inter-Position	80.3±6.3 (69.1, 89.4)	74.7±7.1 (58, 90)	32±6.5 (22.8, 39)
		Single-Position	82.9±1.8 (80.8, 84.8)	75.5±2.7 (71.5, 79.6)	52.6±1.8 (50.6, 54.2)
	*k*NN	*Intra-Position*	84.5±1.8 (82.3, 86.7)	53.3±1.9 (50.8, 56.8)	77.6±5.8 (72.8, 84.1)
		Inter-Position	69.4±6.6 (58.9, 79.8)	47.1±3.3 (39.6, 56.1)	30.2±4.9 (24.1, 35.5)
		Single-Position	72.4±1.4 (70.9, 74.2)	47.4±0.8 (46.3, 48.8)	46±2.1 (43.7, 47.8)
	RF	*Intra-Position*	93.2±0.8 (91.9, 93.8)	84.6±2.3 (80.7, 88.1)	93.7±2.6 (91.4, 96.5)
		Inter-Position	81.2±5.6 (72, 90.1)	73±6.2 (55.3, 85.8)	38.5±5.7 (30.7, 43.3)
		Single-Position	83.6±1.4 (82.1, 85.7)	73.7±2.3 (70, 77.1)	56.7±1.7 (55.6, 58.6)
	SVM	*Intra-Position*	89.9±1.5 (87.7, 91.6)	80.6±2 (77.9, 84.5)	87.9±2.6 (85.7, 90.7)
		Inter-Position	74.4±7.4 (62.3, 85.5)	69.4±6 (54.9, 83.8)	35.8±6.6 (26.6, 42.9)
		Single-Position	77.5±1.8 (75.2, 79.5)	70.1±2 (66.2, 73)	53.1±3.1 (51, 56.7)
TSTD	LDA	*Intra-Position*	96.9±0.6 (96.1, 97.7)	91.2±1.8 (88.7, 94.6)	97±1.1 (95.9, 97.9)
		Inter-Position	86.3±5.9 (75.9, 95.6)	79.9±7 (57.1, 92.5)	38.2±7.7 (31.1, 50.4)
		Single-Position	88.4±0.9 (87.3, 89.7)	80.6±2.6 (76.1, 83.6)	57.8±1.9 (55.7, 59.4)
	QDA	*Intra-Position*	94.5±1 (93.3, 95.8)	87.3±2.3 (83.4, 91.7)	93.8±1.7 (92.1, 95.6)
		Inter-Position	84.5±5.5 (75.4, 92.5)	74.6±7.2 (54.3, 89.6)	39.2±6.6 (30.9, 46.3)
		Single-Position	86.5±0.8 (85.7, 87.5)	75.4±1.8 (71.9, 78.1)	57.4±1.9 (55.3, 59.2)
	*k*NN	*Intra-Position*	92.7±1 (91.2, 93.8)	57.2±1.9 (54.9, 60.3)	93.4±1.3 (92.4, 94.9)
		Inter-Position	82.4±4.7 (73.5, 88.9)	48.4±4.5 (38.5, 57.7)	45.6±7.7 (36, 54.5)
		Single-Position	84.5±1.1 (82.7, 85.8)	48.9±1.7 (46.5, 51.2)	61.6±1.7 (59.7, 62.9)
	RF	*Intra-Position*	94.3±0.8 (93, 95)	86.1±2.1 (82.8, 89.2)	93.2±2.4 (91.5, 95.9)
		Inter-Position	83.3±5.4 (73.9, 91.5)	74.1±6.6 (56, 87.8)	41.6±7 (33.1, 48.3)
		Single-Position	85.4±1.2 (84, 87.2)	74.8±2.4 (71.3, 78.5)	58.3±0.9 (57.2, 58.9)
	SVM	*Intra-Position*	94.9±0.6 (93.9, 95.3)	86.7±2.1 (83.5, 90.1)	94.4±1.4 (93.2, 95.9)
		Inter-Position	85.6±5.1 (76.6, 92.7)	75.1±6.5 (59.4, 88.6)	45.8±7.4 (35.6, 54.8)
		Single-Position	87.4±1 (86, 88.7)	75.9±2.6 (71.6, 79.3)	62±1.2 (60.7, 63.1)

Table S1. Mean and Minimum accuracy of Intra-Position, Inter-Position and Single-Position testing regimes demonstrating degradation in the presence of the limb-position effect.

Feature Set	Classifier	Test Regime	7 Intensity Mean±SD (Min, Max)	3 Intensity Mean±SD (Min, Max)	3 Intensity Mean±SD (Min, Max)
TD	LDA	*Intra-Level*	94±5.8 (83, 99.2)	92.7±2.9 (89.5, 95.2)	96.1±2 (93.8, 97.8)
		Inter-Level	67.9±26 (19.7, 99)	67.2±19.2 (36.7, 90.2)	71.4±11.9 (55.5, 81.5)
		Single-Level	71.7±6.6 (61.2, 79.3)	75.7±6.4 (68.4, 79.9)	79.6±5.7 (76, 86.2)
	QDA	*Intra-Level*	90.6±6.2 (80.3, 96.4)	90.1±3.1 (87.1, 93.3)	94.1±1.3 (93, 95.5)
		Inter-Level	73.2±15.8 (41.1, 95.3)	71.6±9.9 (59.6, 84)	65.9±17.7 (40.2, 88)
		Single-Level	75.7±7.7 (61.2, 83)	77.8±4.5 (72.7, 81.3)	75.3±10.1 (63.7, 81.1)
	*k*NN	*Intra-Level*	90.4±10 (71.8, 98.9)	88.4±7.8 (79.9, 95.2)	87.1±6.4 (82.4, 94.4)
		Inter-Level	65.9±24.6 (19.5, 98)	64±17.1 (36.3, 83.6)	54.1±10.7 (38, 63.8)
		Single-Level	69.4±6.2 (60.9, 76.7)	72.1±3.8 (68.7, 76.2)	65.1±3.6 (62.7, 69.2)
	RF	*Intra-Level*	93.3±6.6 (80.3, 98.9)	90.8±3.7 (88, 95)	93.8±3.3 (90.7, 97.2)
		Inter-Level	59.5±24.9 (17.5, 96.8)	54.1±16.4 (29.6, 74.9)	58±13.5 (38.5, 72.3)
		Single-Level	64.2±7.2 (51.5, 71.3)	66.7±4.6 (63.8, 71.9)	69.3±6.7 (65.1, 77)
	SVM	*Intra-Level*	94.1±6.3 (81.6, 99.2)	93.4±3.2 (90.3, 96.7)	92.8±1.8 (91.7, 94.9)
		Inter-Level	67.2±25.1 (19.3, 98.4)	65.7±17.6 (36.7, 85.8)	59.4±12 (40.5, 71.2)
		Single-Level	71±6.1 (63, 78.5)	75±4.3 (70.2, 78.7)	70.6±6 (64.1, 76)
LSF4	LDA	*Intra-Level*	94.8±5.1 (84.8, 99.2)	94.4±3.1 (91.5, 97.7)	97.3±1.1 (96.1, 98.4)
		Inter-Level	71.2±22.6 (25.1, 98.8)	69.4±14.8 (50.8, 86.5)	75.4±13.4 (54.7, 87.6)
		Single-Level	74.6±6.3 (63.7, 82)	77.7±4.3 (73.4, 82)	82.7±5.8 (77.2, 88.8)
	QDA	*Intra-Level*	93.2±6 (82.1, 98.6)	92.3±3.5 (88.9, 95.8)	94.9±0.8 (94, 95.5)
		Inter-Level	71.5±19.3 (28.3, 96.4)	68.5±12.7 (51.3, 85)	64.3±18.8 (36, 87.2)
		Single-Level	74.6±6.9 (61.6, 80.9)	76.4±3.2 (72.9, 79)	74.5±11.2 (61.6, 81.7)
	*k*NN	*Intra-Level*	89±5.2 (78.4, 92.9)	85.8±3.8 (82.6, 90.1)	90.4±2.1 (88.1, 92.3)
		Inter-Level	59.8±20.5 (26.5, 90.8)	55.8±13.7 (40.9, 72.3)	58.5±10 (44.9, 66.2)
		Single-Level	64±8.4 (47.7, 71.2)	65.8±5.8 (59.3, 70.4)	69.1±3.8 (66.9, 73.5)
	RF	*Intra-Level*	93.2±6.4 (80.8, 99)	90.3±4 (87.1, 94.8)	93.5±3.3 (90.1, 96.6)
		Inter-Level	59.8±24.6 (18.4, 96.3)	53.4±15.5 (29.4, 71.3)	58.5±14.1 (38.7, 73.6)
		Single-Level	64.4±8.2 (49.4, 72.3)	65.8±4.7 (63, 71.2)	69.7±5 (65.9, 75.4)
	SVM	*Intra-Level*	92±5.6 (81.2, 96.6)	89.6±3.9 (86, 93.8)	91.2±1.5 (89.9, 92.8)
		Inter-Level	63±21 (26.7, 94.1)	59.5±14.5 (42.8, 76.7)	60.2±10 (44.4, 70.6)
		Single-Level	67.2±8 (52, 75)	69.6±6.1 (62.9, 74.7)	70.5±3.8 (66.5, 74.2)
LSF9	LDA	*Intra-Level*	95.2±4.7 (86, 99.4)	94.4±3 (91.4, 97.4)	97.6±0.8 (96.7, 98.1)
		Inter-Level	64.4±27.8 (17.7, 98.5)	62.2±17.6 (40.5, 83.4)	70.4±19.5 (35.9, 88.2)
		Single-Level	68.8±11.8 (46.2, 80.2)	73±7.4 (64.7, 79)	79.5±9.4 (68.9, 87.1)
	QDA	*Intra-Level*	90.8±6.6 (78.9, 96.7)	89.8±4 (85.8, 93.8)	92.1±1 (91.1, 92.9)
		Inter-Level	69.5±18 (29.7, 95.1)	65.5±11.4 (49.5, 81.6)	61.7±19.4 (36, 86.3)
		Single-Level	72.6±7.4 (58.5, 79.8)	73.6±3.4 (69.8, 76.1)	71.8±11.2 (58.9, 78.8)
	*k*NN	*Intra-Level*	93.4±6.6 (80.5, 98.7)	91.6±4.9 (86.7, 96.6)	92.7±2.8 (89.7, 95.4)
		Inter-Level	66.5±23.3 (22.8, 98.1)	63.1±16.1 (41, 83.5)	60.2±10.5 (45.2, 68)
		Single-Level	70.3±6.9 (58.5, 78.1)	72.6±4.8 (68.2, 77.7)	71±3.8 (68.6, 75.4)
	RF	*Intra-Level*	92.9±6.8 (79.3, 98.7)	90.7±3.4 (88.3, 94.5)	93.5±3 (90.4, 96.3)
		Inter-Level	59.6±24.8 (17.6, 96.6)	53.5±15.5 (31.2, 72.2)	58.3±13.6 (37.8, 74.5)
		Single-Level	64.1±8.1 (49.7, 72.7)	66.2±4.2 (63.7, 71.1)	70.4±5.5 (67.1, 76.8)
	SVM	*Intra-Level*	93.9±6 (82, 98.8)	93.2±3.5 (89.7, 96.6)	94±2.2 (91.8, 96.1)
		Inter-Level	67.7±23 (22, 98.4)	66.6±15.4 (44.1, 85.8)	62.8±11.3 (45.1, 73.3)
		Single-Level	71.5±6.7 (61.1, 79.8)	75.5±3.6 (73.2, 79.6)	73.2±4.7 (68.7, 78)
TDPSD	LDA	*Intra-Level*	93.8±5.6 (82.6, 98.9)	93.4±2.9 (91.1, 96.7)	95.7±1.4 (94.2, 96.9)
		Inter-Level	73.9±19.1 (31.7, 98)	74.5±11.8 (57.6, 88.2)	69.1±13.1 (51.7, 80.8)
		Single-Level	76.7±6.4 (63.6, 82.4)	80.8±3.1 (78.2, 84.3)	78±5.3 (73.8, 83.9)
	QDA	*Intra-Level*	92.4±6.8 (79.7, 98.3)	90.4±3.9 (86.8, 94.6)	93.3±1.8 (91.5, 95)
		Inter-Level	67.2±24.4 (18, 96.9)	64.3±20.1 (31.8, 85.6)	56.6±12.9 (38.1, 68.3)
		Single-Level	70.8±6.6 (61.3, 78.6)	73±5.8 (66.4, 77.5)	68.9±5.7 (64.1, 75.2)
	*k*NN	*Intra-Level*	84.5±8.8 (69, 93.8)	82±6.9 (74.3, 87.6)	78.9±7.7 (73.2, 87.7)
		Inter-Level	65.2±17.5 (27.7, 89.8)	61.2±11.9 (44, 73.8)	42.4±10 (27.3, 51.7)
		Single-Level	68±7.1 (54.5, 74.1)	68.2±4.4 (63.6, 72.4)	54.5±3.4 (52.4, 58.5)
	RF	*Intra-Level*	93.2±6.1 (81.8, 98.8)	90.5±3.5 (87.5, 94.4)	92.7±3.4 (89.3, 96)
		Inter-Level	61±24.4 (17.6, 96.7)	54.9±16 (30.3, 74.1)	57.1±14.1 (36.1, 73.2)
		Single-Level	65.5±7.6 (51.9, 72.8)	66.8±3.9 (64.1, 71.2)	69.1±6.4 (64.9, 76.4)
	SVM	*Intra-Level*	89.5±6.8 (77, 96.1)	87.8±4.7 (82.5, 91.5)	88.3±3.3 (85.6, 91.9)
		Inter-Level	67.1±19.8 (25.1, 93.8)	63.3±13.6 (42, 77.8)	50.1±12.2 (31, 61.2)
		Single-Level	70.3±6.2 (58.7, 76.7)	71.4±3.4 (69.5, 75.4)	62.8±5.1 (58, 68.2)
TSTD	LDA	*Intra-Level*	95±5 (85.1, 99.2)	94.1±3.4 (91.1, 97.7)	96.9±1.1 (95.7, 97.7)
		Inter-Level	71.2±23.1 (25.7, 98.9)	68.4±15.8 (49.6, 87.8)	69±17.4 (40.4, 84.7)
		Single-Level	74.6±7.9 (59.3, 82.5)	77±6 (70.9, 82.9)	78.3±8.6 (69.3, 86.6)
	QDA	*Intra-Level*	93.2±6.7 (80.4, 99)	91.5±3.7 (88, 95.3)	93.1±1.7 (91.1, 94.4)
		Inter-Level	69.8±20.2 (26.9, 96.7)	65.7±13.6 (46.6, 82.6)	55.8±13.2 (38, 71.1)
		Single-Level	73.2±5.6 (62.6, 78.5)	74.3±2.4 (72.4, 77)	68.2±6.9 (61.1, 74.9)
	*k*NN	*Intra-Level*	90.1±5.3 (79.6, 94.3)	89.2±3.4 (86, 92.8)	92.6±3.1 (89.3, 95.4)
		Inter-Level	64.5±20.3 (27.7, 92.6)	60.6±14.6 (43.6, 78.3)	60±10.5 (45, 67.9)
		Single-Level	68.2±8.5 (51.6, 75.5)	70.1±6.2 (63.2, 75.1)	70.9±3.8 (68.5, 75.3)
	RF	*Intra-Level*	93.4±6.3 (81.2, 98.7)	91.5±3.7 (88.8, 95.7)	92.6±3.5 (89.6, 96.4)
		Inter-Level	60.3±24.5 (19.5, 97.1)	55.1±16.9 (31.2, 76.9)	56.3±13 (37.2, 71.8)
		Single-Level	65.1±8 (50.3, 73.4)	67.4±4 (65, 72.1)	67.6±5.8 (63.5, 74.3)
	SVM	*Intra-Level*	91.8±5.3 (81.6, 96.1)	91±2.9 (88.2, 94)	93.8±2.4 (91.3, 96.1)
		Inter-Level	66.3±20.4 (28.4, 94.1)	62.7±13.7 (46.8, 77.8)	62.7±11.1 (45.1, 72.9)
		Single-Level	69.9±8.1 (54.2, 77.8)	72.1±5.2 (66.7, 77)	73.1±4.5 (68.8, 77.8)

Table S2. Mean and Minimum accuracy of Intra-Level, Inter-Level and Single-Level testing regimes demonstrating degradation in the presence of the contraction-intensity effect.

© 2020 by the authors. Licensee MDPI, Basel, Switzerland. This article is an open access article distributed under the terms and conditions of the Creative Commons Attribution (CC BY) license (http://creativecommons.org/licenses/by/4.0/).
