# Supplementary figures and images for: Current Trends and Confounding Factors in Myoelectric Control: Limb Position and Contraction Intensity
Source: Sensors (Basel). 2020 Mar 13;20(6):1613. doi: 10.3390/s20061613 (PMC7146367; doi:10.3390/s20061613)

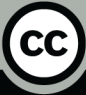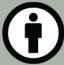

BY

Supplement: Supplementary file 1 [file sensors-20-01613-s001.zip › sensors-725895 - SI/Definitions/logo-ccby-eps-converted-to.pdf]

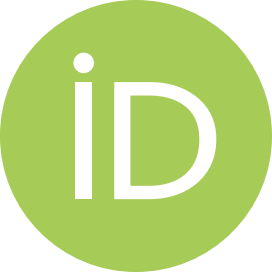

Supplement: Supplementary file 1 [file sensors-20-01613-s001.zip › sensors-725895 - SI/Definitions/logo-orcid-eps-converted-to.pdf]

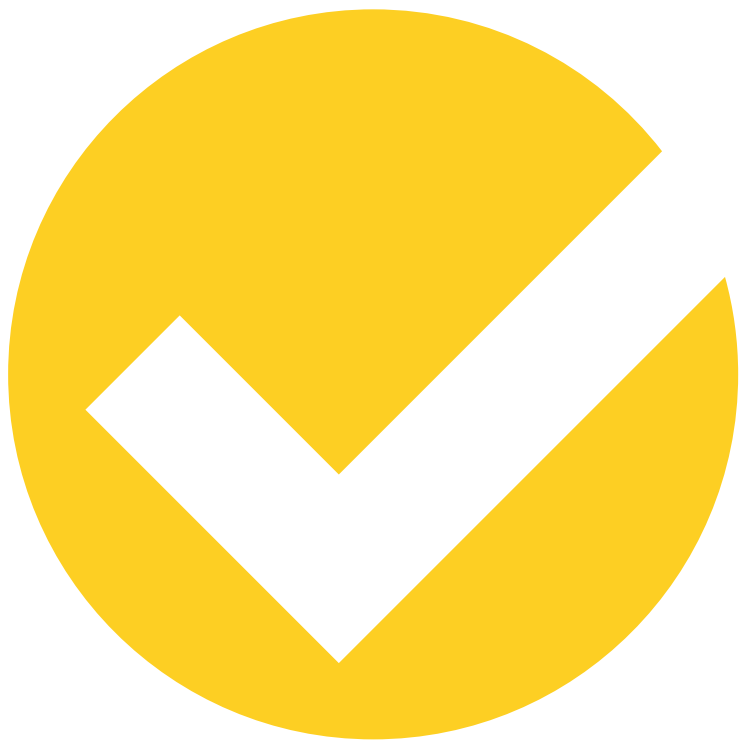

check for  
updates

Supplement: Supplementary file 1 [file sensors-20-01613-s001.zip › sensors-725895 - SI/Definitions/logo-updates.pdf]

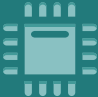

*sensors*

Supplement: Supplementary file 1 [file sensors-20-01613-s001.zip › sensors-725895 - SI/Definitions/sensors-logo-eps-converted-to.pdf]

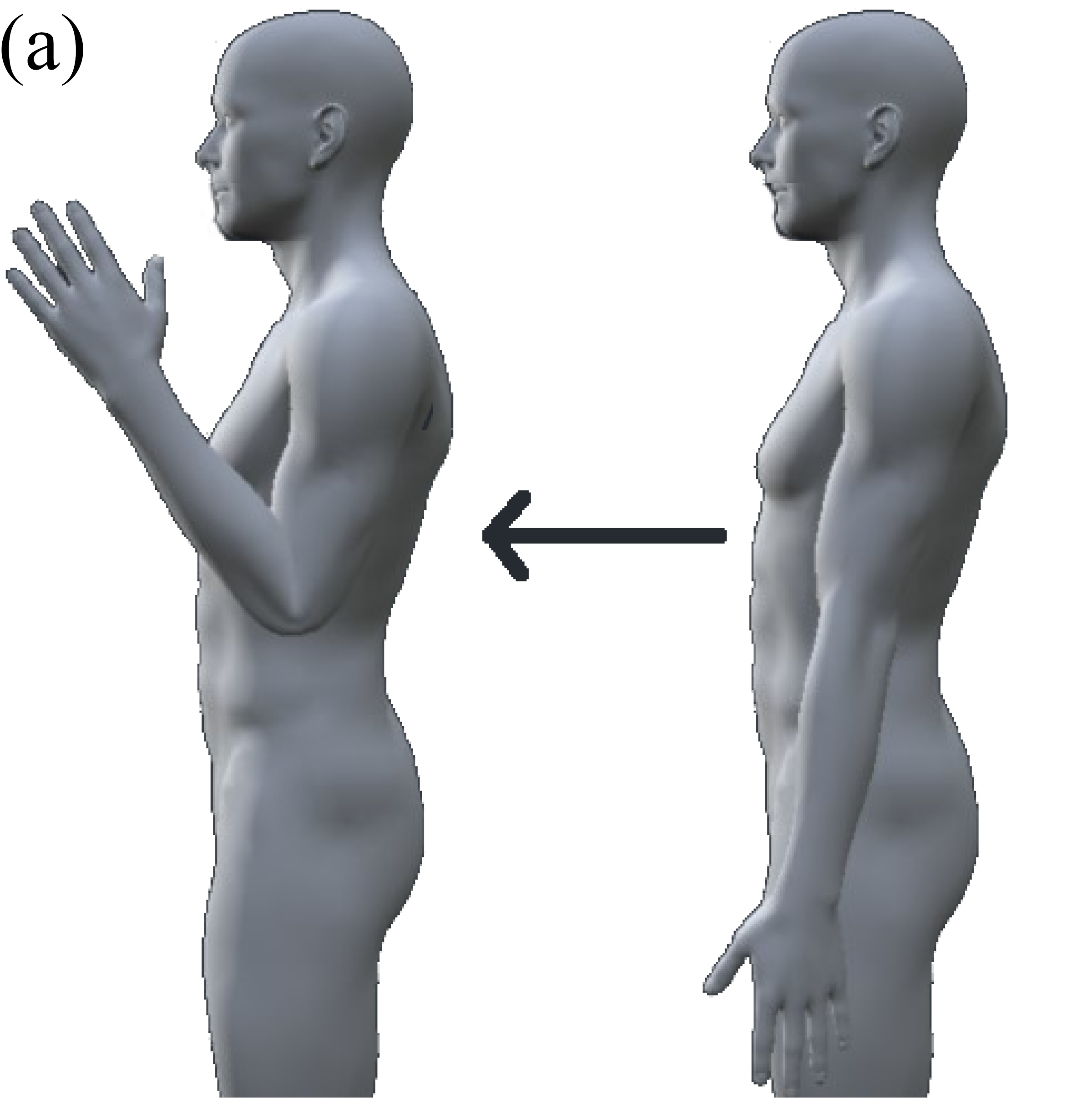

Supplement: Supplementary file 1 [file sensors-20-01613-s001.zip › sensors-725895 - SI/Figures/2D_DynamicFromStatic.png]

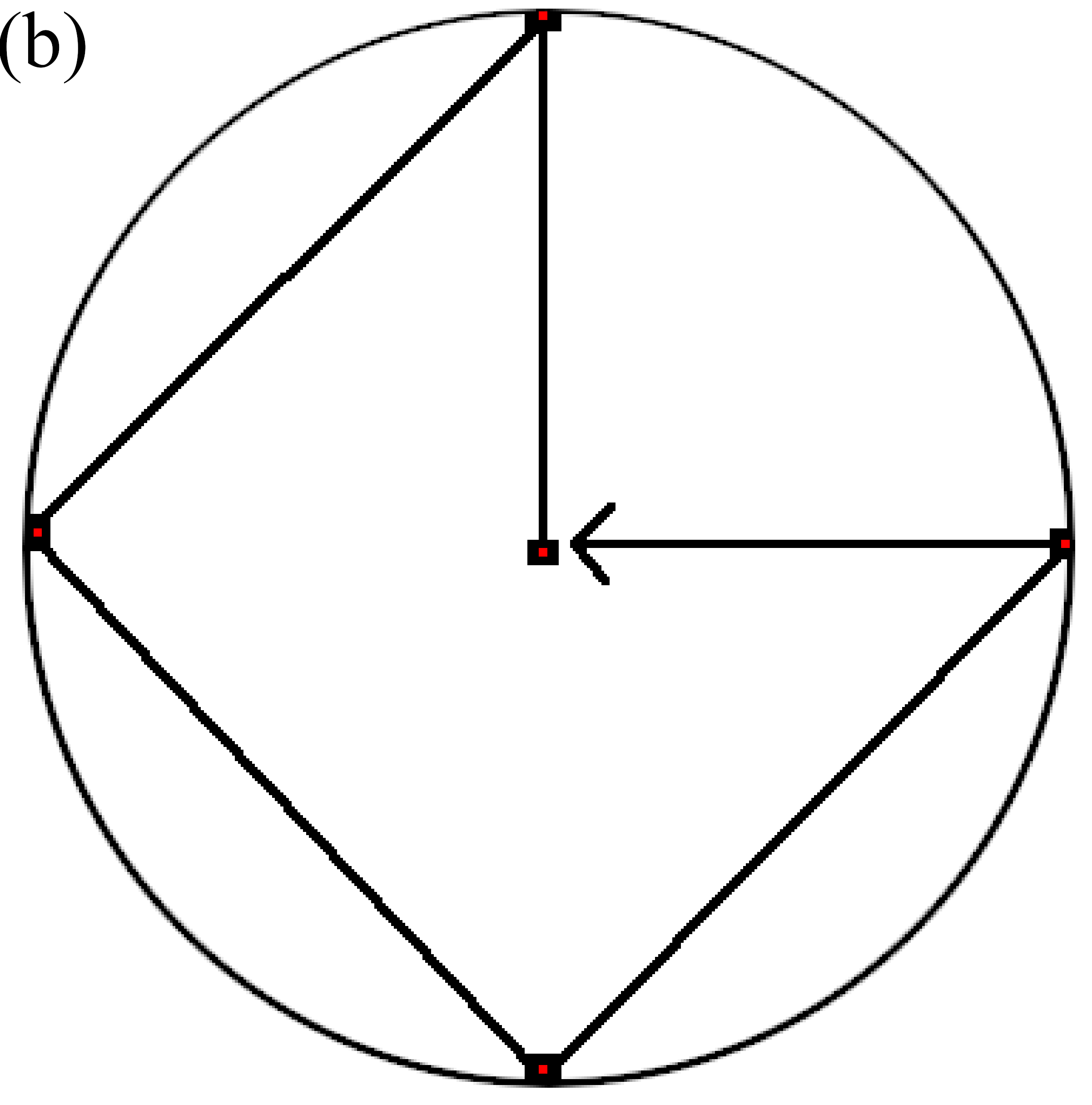

Supplement: Supplementary file 1 [file sensors-20-01613-s001.zip › sensors-725895 - SI/Figures/2D_DynamicFromTraj.png]

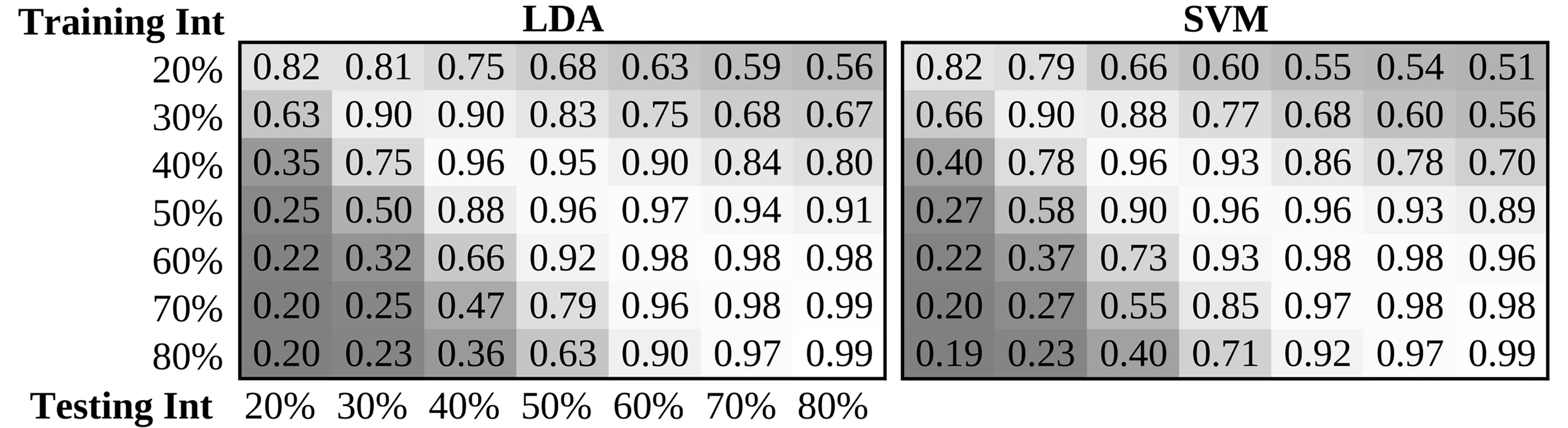

Supplement: Supplementary file 1 [file sensors-20-01613-s001.zip › sensors-725895 - SI/Figures/ContConfuse.png]

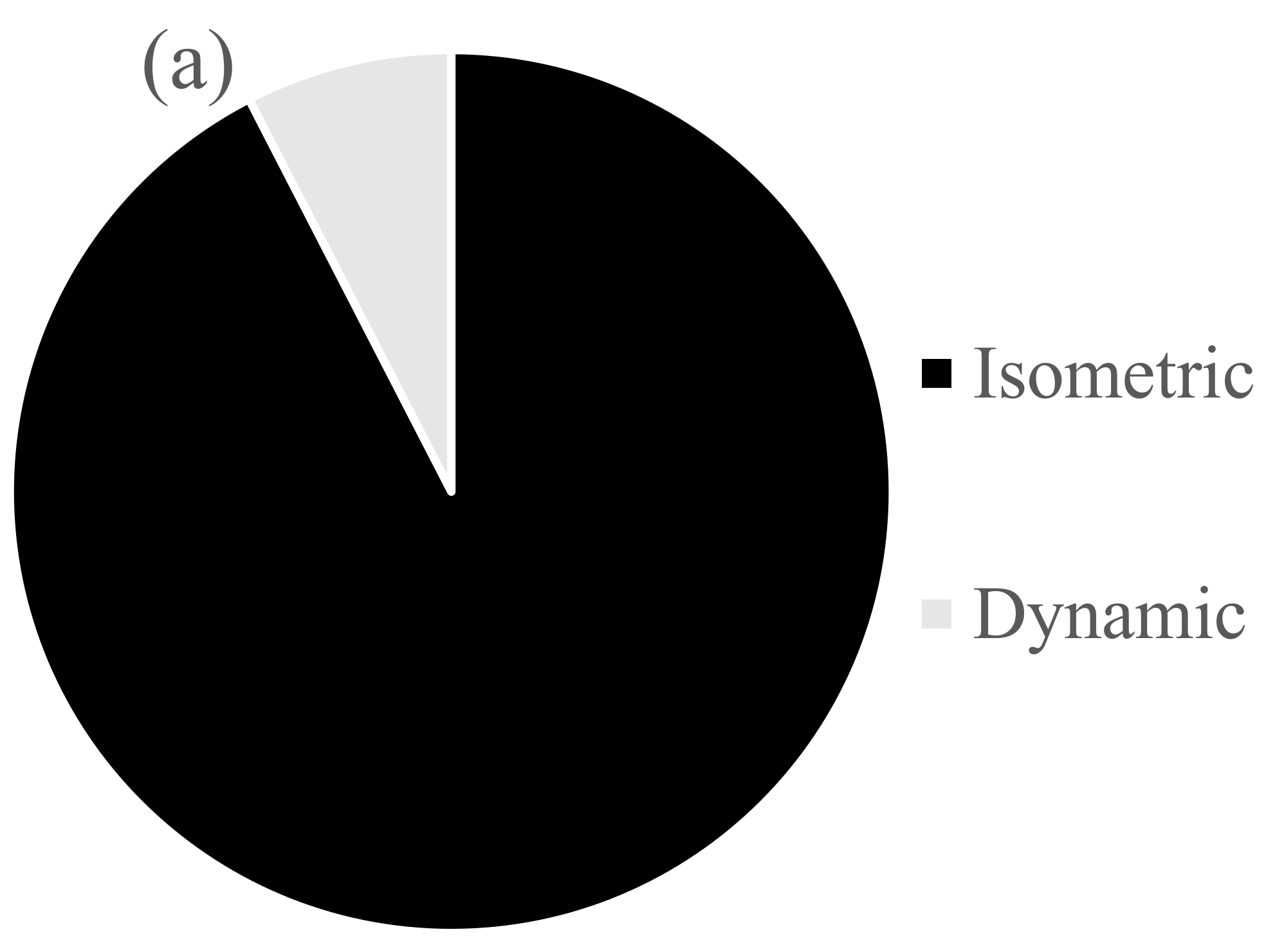

Supplement: Supplementary file 1 [file sensors-20-01613-s001.zip › sensors-725895 - SI/Figures/Cont_Lit_A.png]

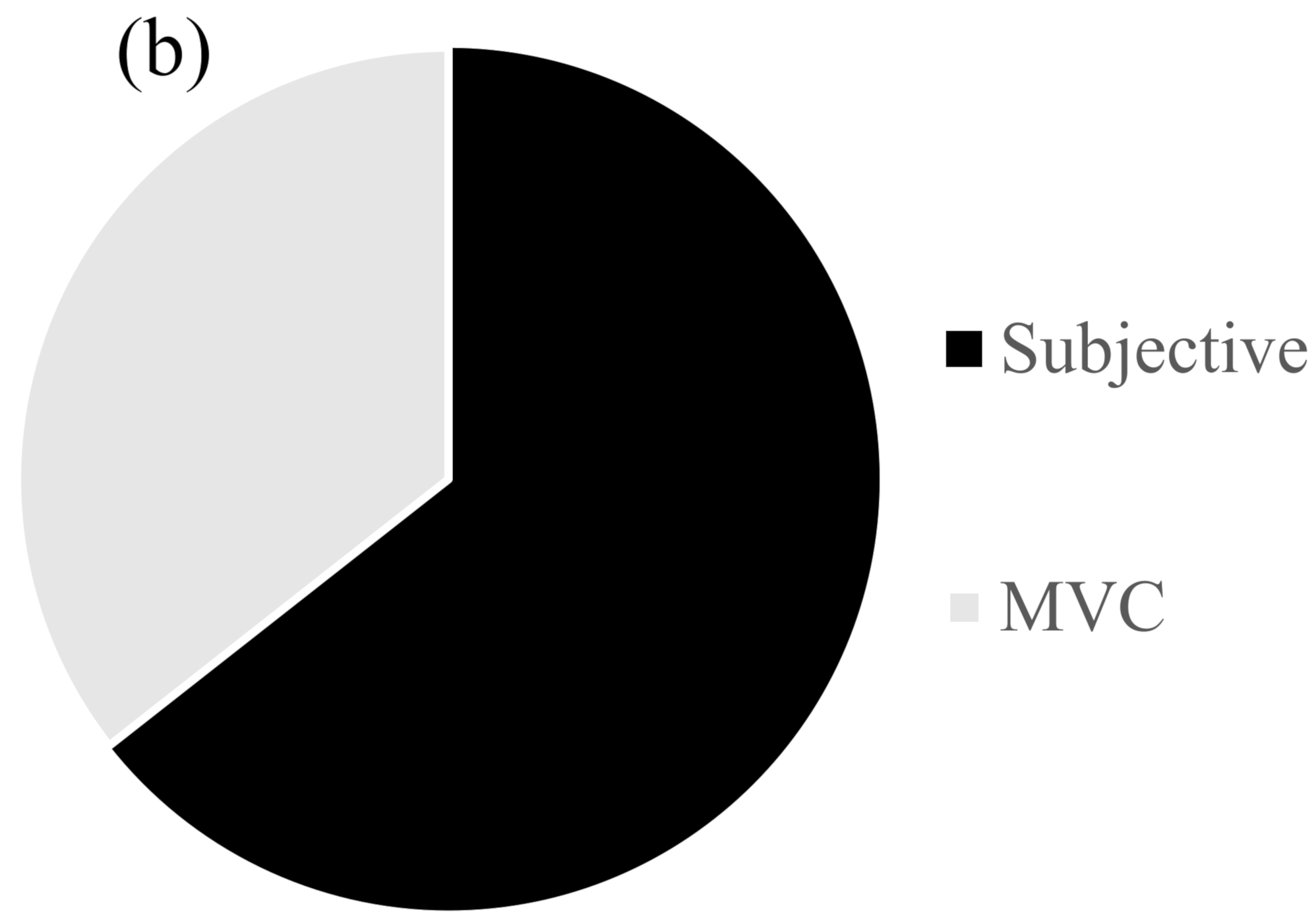

Supplement: Supplementary file 1 [file sensors-20-01613-s001.zip › sensors-725895 - SI/Figures/Cont_Lit_B.png]

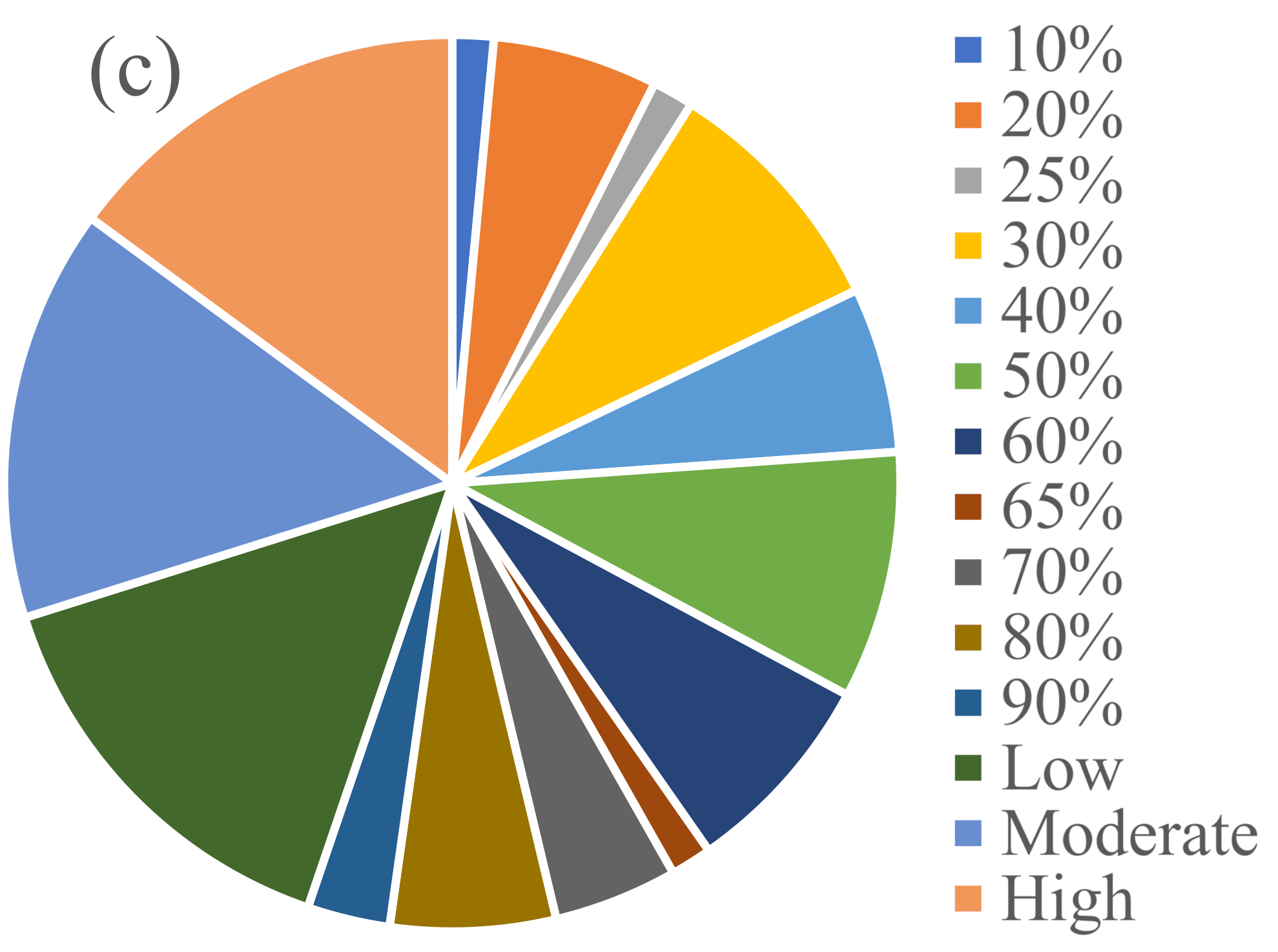

Supplement: Supplementary file 1 [file sensors-20-01613-s001.zip › sensors-725895 - SI/Figures/Cont_Lit_C.png]

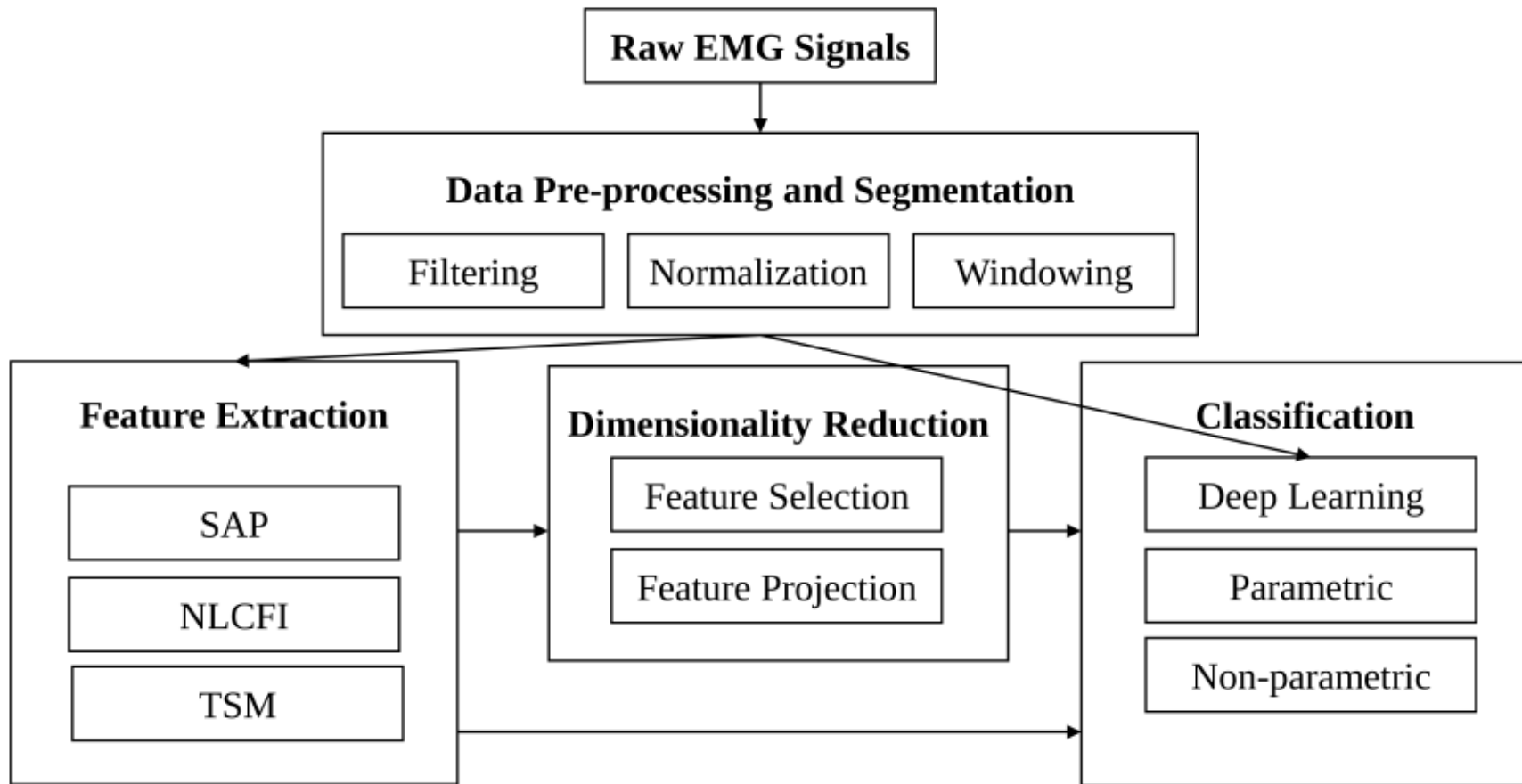

Supplement: Supplementary file 1 [file sensors-20-01613-s001.zip › sensors-725895 - SI/Figures/EMGPR_architecture.pdf]

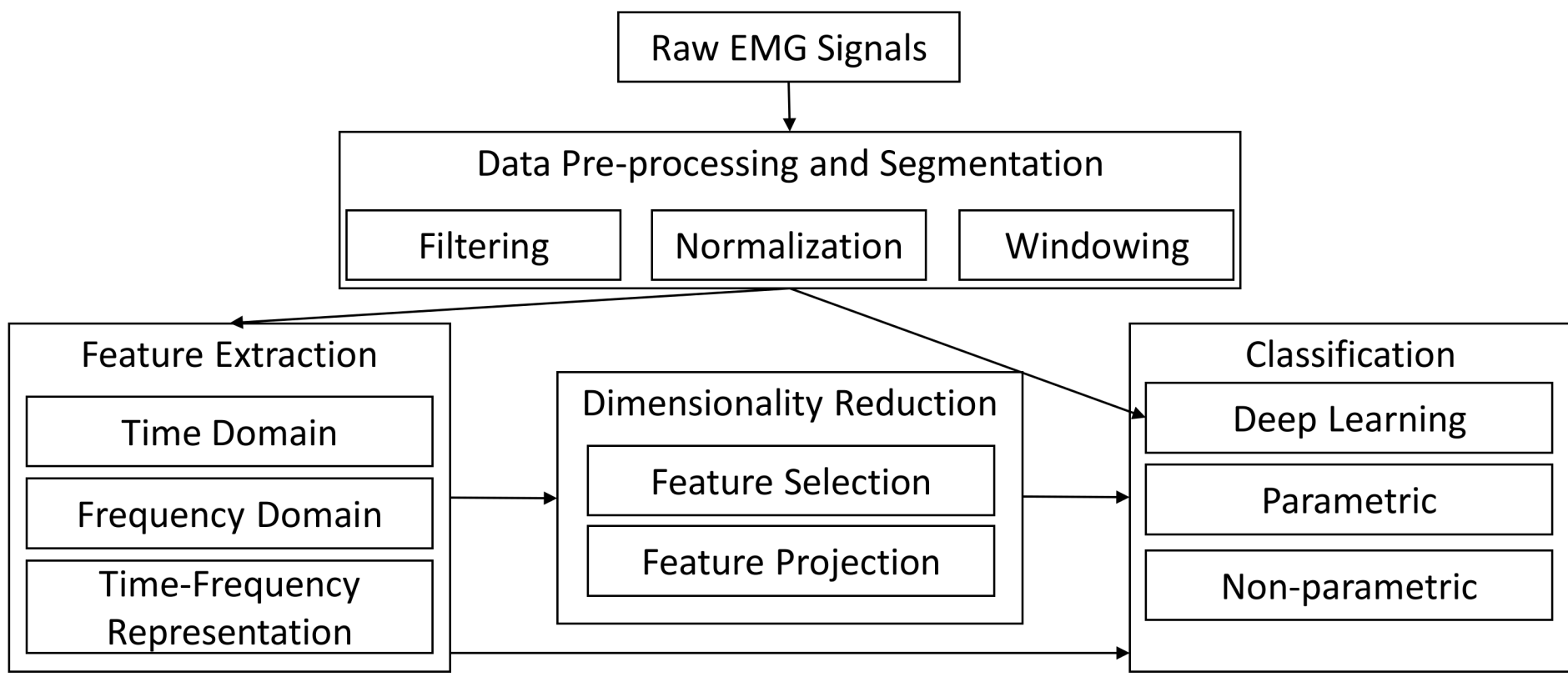

Supplement: Supplementary file 1 [file sensors-20-01613-s001.zip › sensors-725895 - SI/Figures/EMGPR_pipeline_old.pdf]

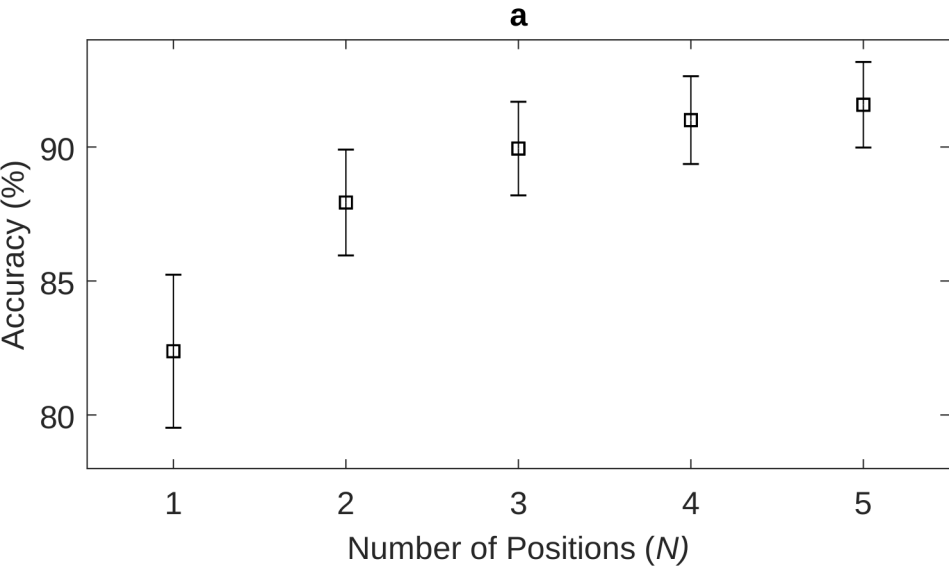

**b**

| $N$ | 1   | 2   | 3   | 4   | 5   |
|-----|-----|-----|-----|-----|-----|
| 1   |     | $x$ | $x$ | $x$ | $x$ |
| 2   | $x$ |     |     | $x$ | $x$ |
| 3   | $x$ |     |     |     |     |
| 4   | $x$ | $x$ |     |     |     |
| 5   | $x$ | $x$ |     |     |     |

Supplement: Supplementary file 1 [file sensors-20-01613-s001.zip › sensors-725895 - SI/Figures/Fougner_LP_NvsAll_old.pdf]

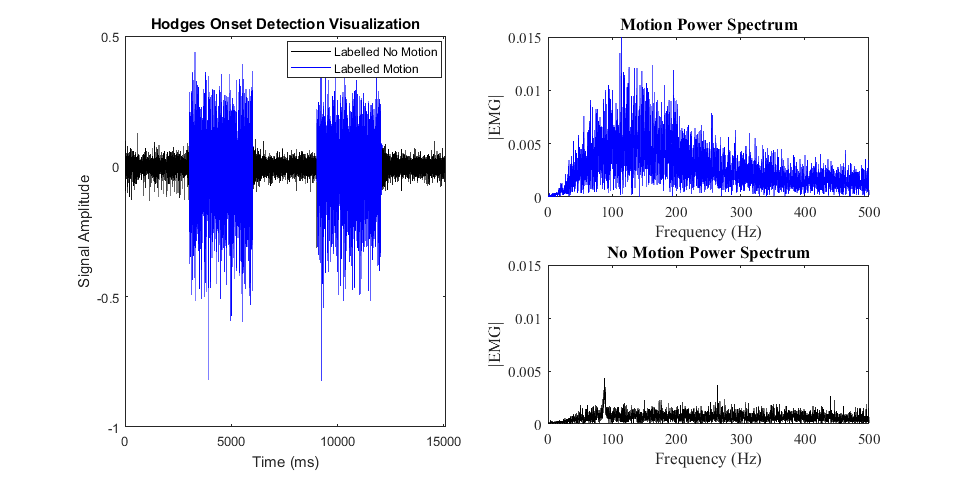

Supplement: Supplementary file 1 [file sensors-20-01613-s001.zip › sensors-725895 - SI/Figures/HodgesOnsetDetection.png]

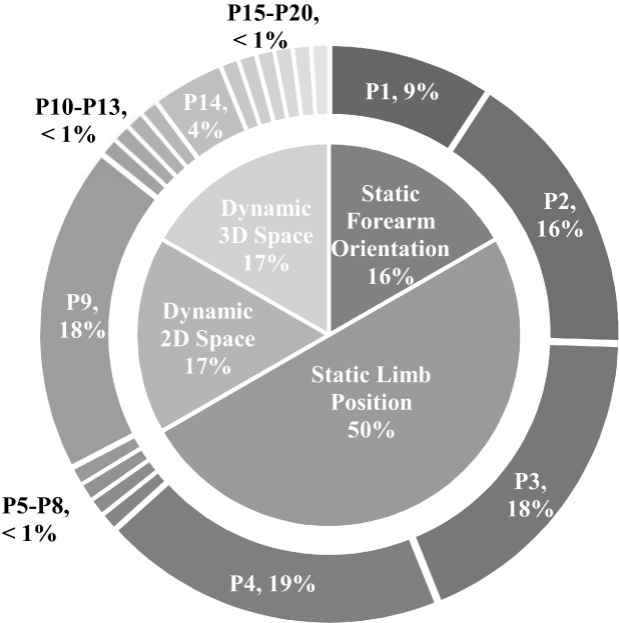

Supplement: Supplementary file 1 [file sensors-20-01613-s001.zip › sensors-725895 - SI/Figures/LitPlots.pdf]

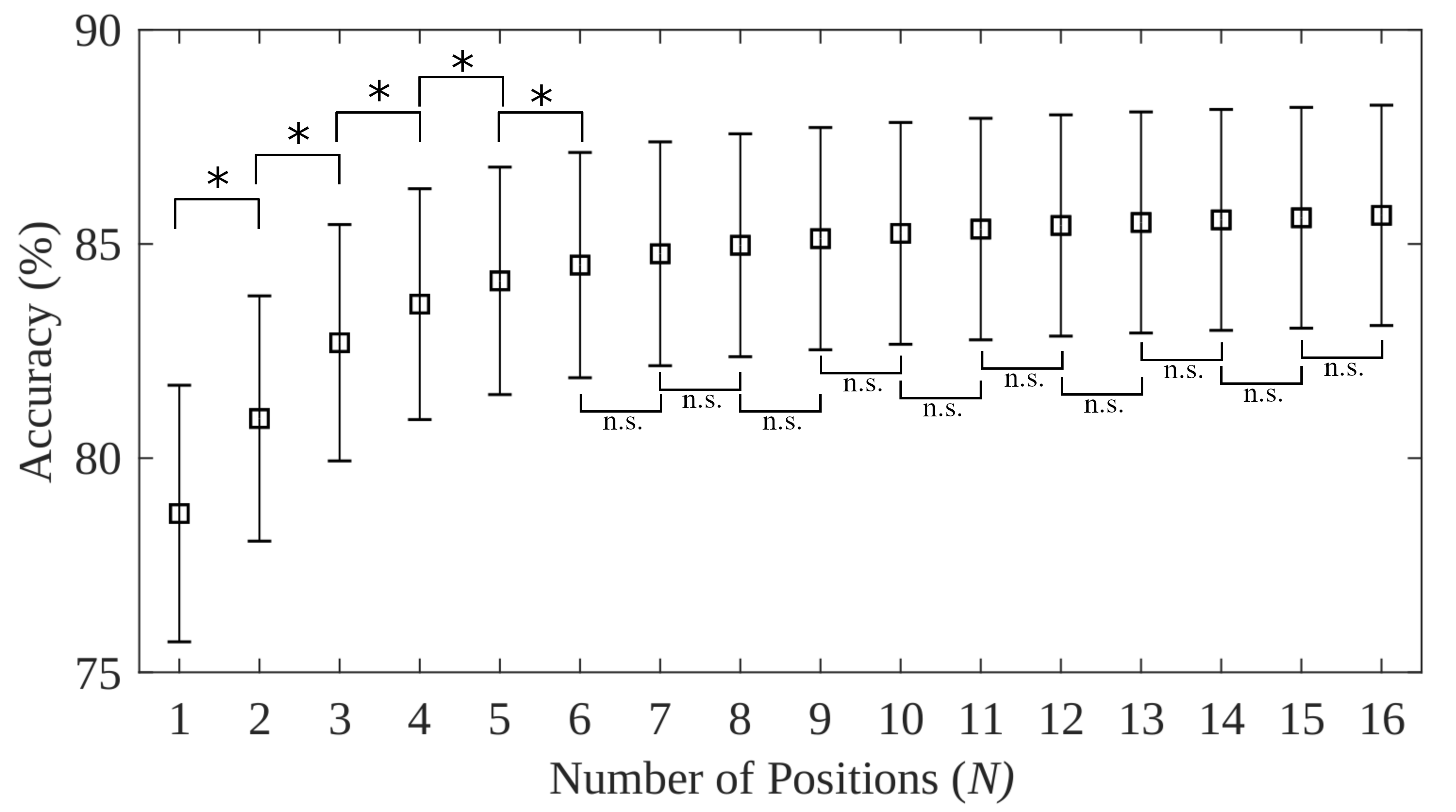

Supplement: Supplementary file 1 [file sensors-20-01613-s001.zip › sensors-725895 - SI/Figures/LP_NvsAll.png]

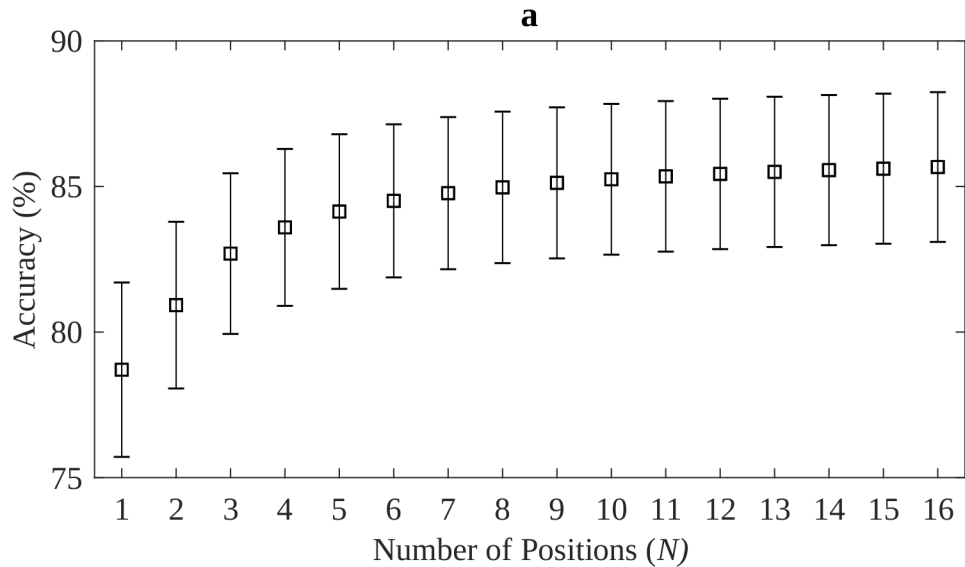

**b**

| $N$ | 1   | 2   | 3   | 4   | 5   | 6   |
|-----|-----|-----|-----|-----|-----|-----|
| 1   |     | $x$ | $x$ | $x$ | $x$ | $x$ |
| 2   | $x$ |     | $x$ | $x$ | $x$ | $x$ |
| 3   | $x$ | $x$ |     |     | $x$ | $x$ |
| 4   | $x$ | $x$ |     |     |     |     |
| 5   | $x$ | $x$ | $x$ |     |     |     |
| 6   | $x$ | $x$ | $x$ |     |     |     |

Supplement: Supplementary file 1 [file sensors-20-01613-s001.zip › sensors-725895 - SI/Figures/LP_NvsAll_old.pdf]

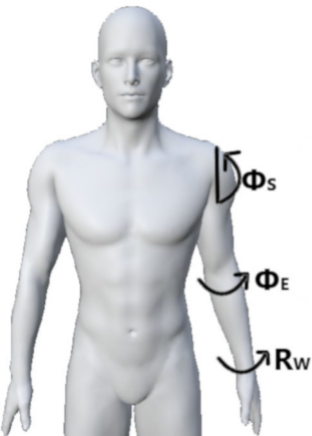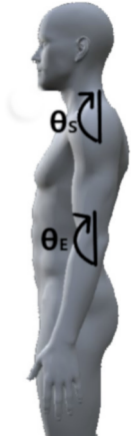

Supplement: Supplementary file 1 [file sensors-20-01613-s001.zip › sensors-725895 - SI/Figures/Positions-eps-converted-to.pdf]

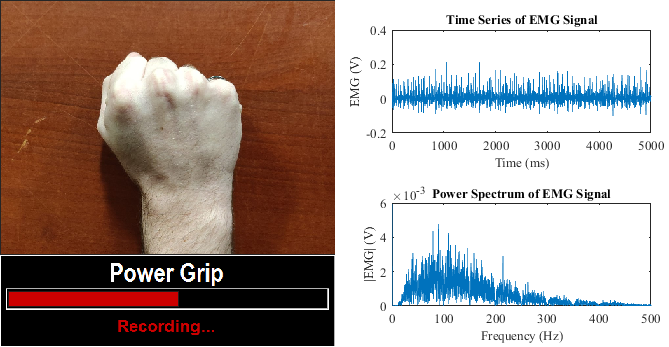

Supplement: Supplementary file 1 [file sensors-20-01613-s001.zip › sensors-725895 - SI/Figures/Prompt_Training.png]

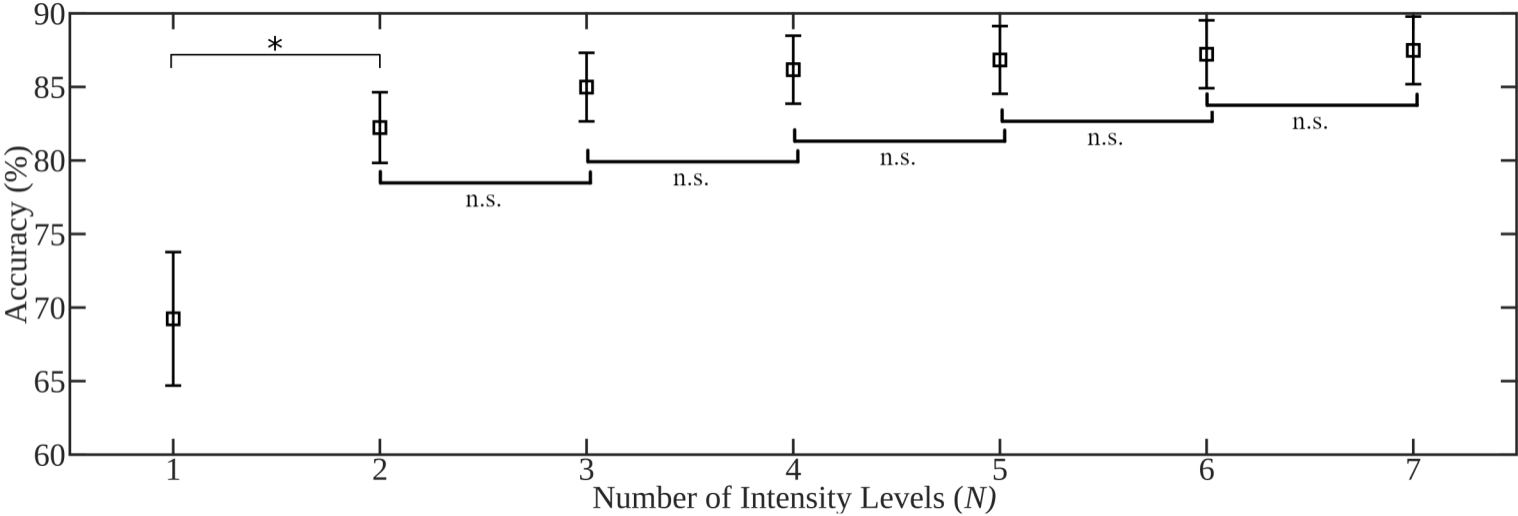

Supplement: Supplementary file 1 [file sensors-20-01613-s001.zip › sensors-725895 - SI/Figures/schemeA_Nvsall.pdf]

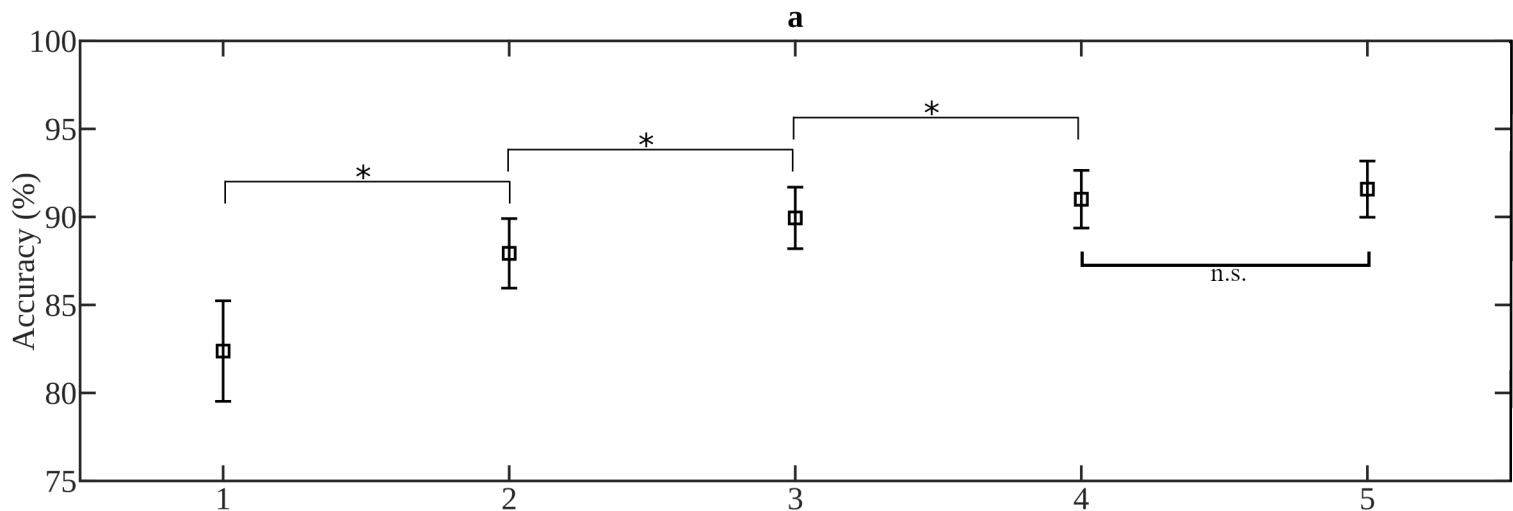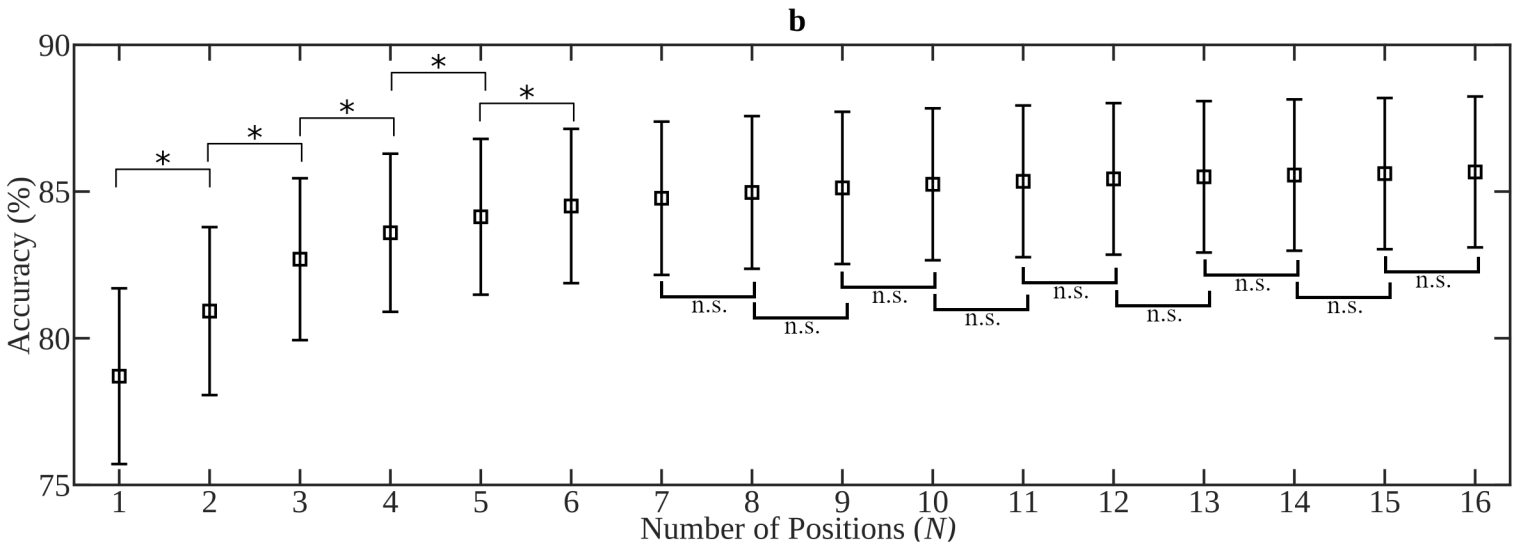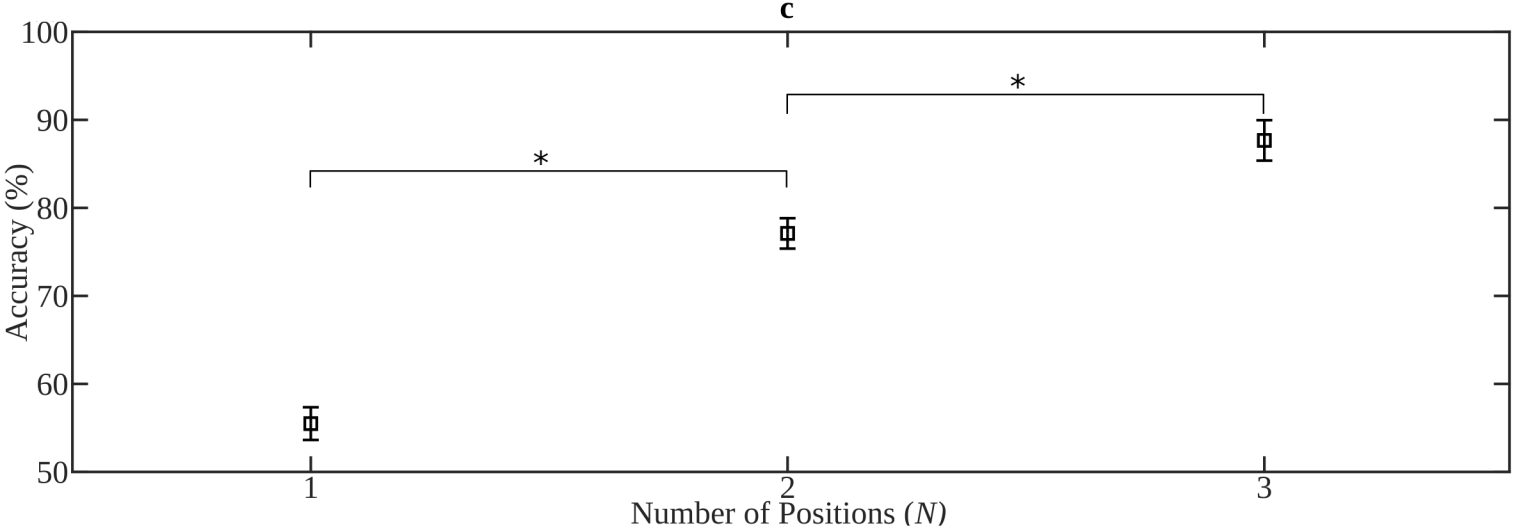

Supplement: Supplementary file 1 [file sensors-20-01613-s001.zip › sensors-725895 - SI/Figures/Suplimentary_LP_N_vs_all.pdf]

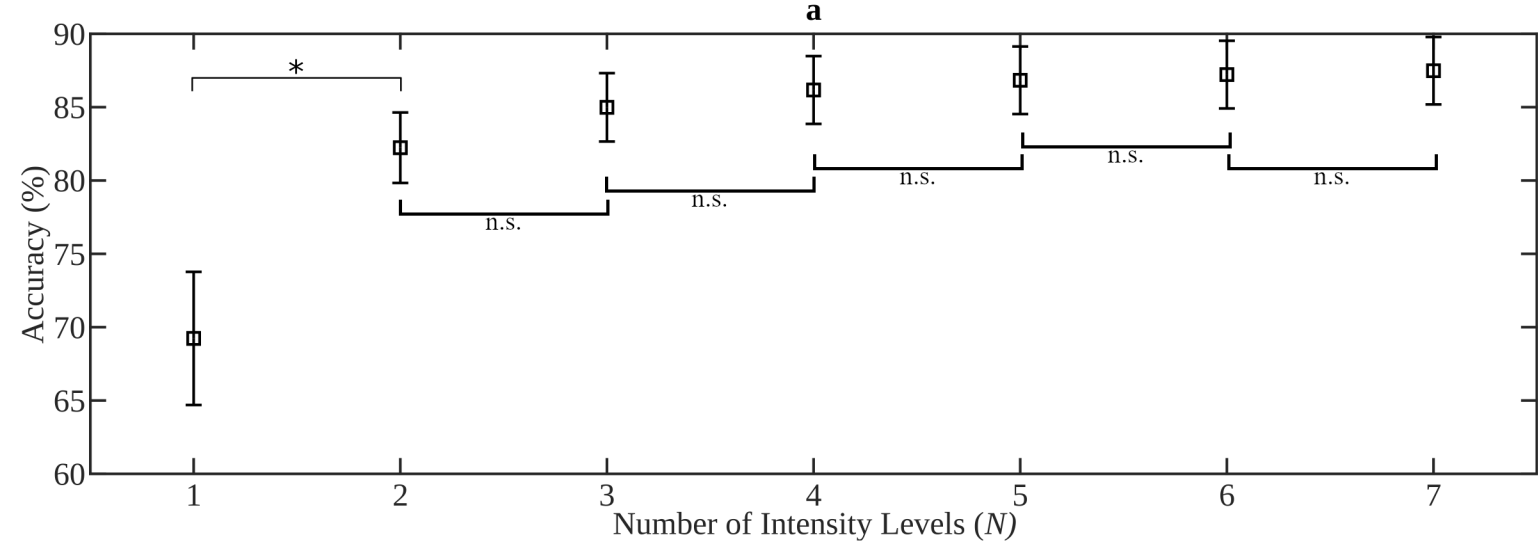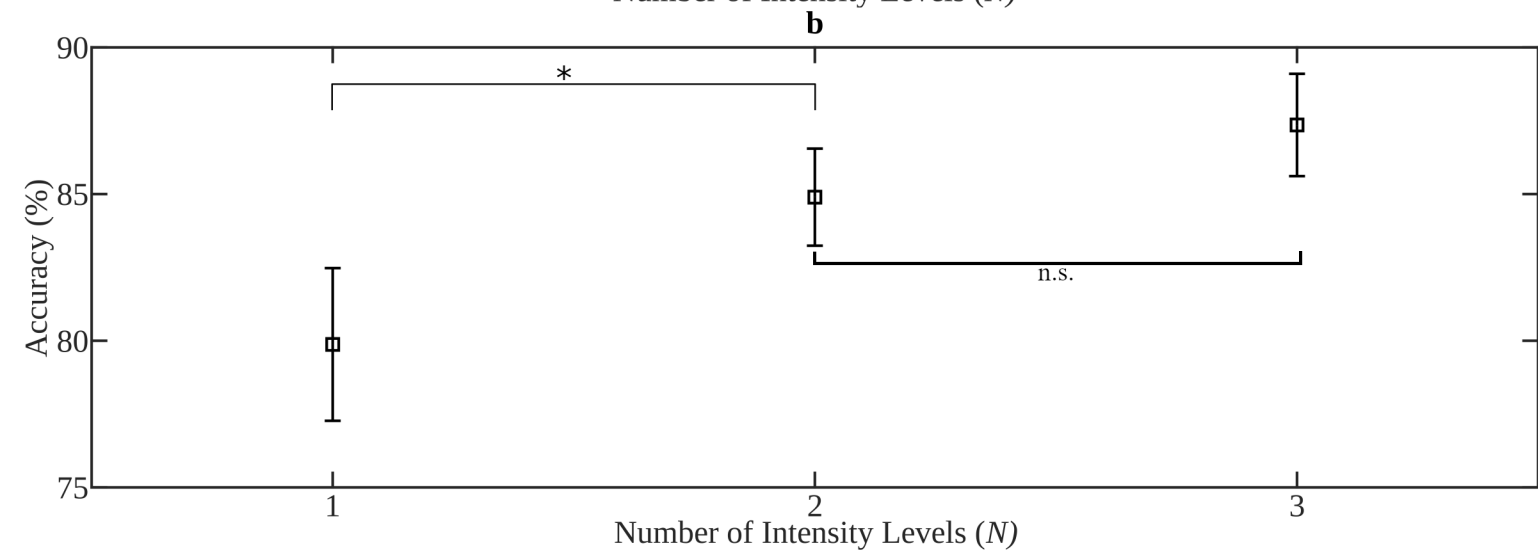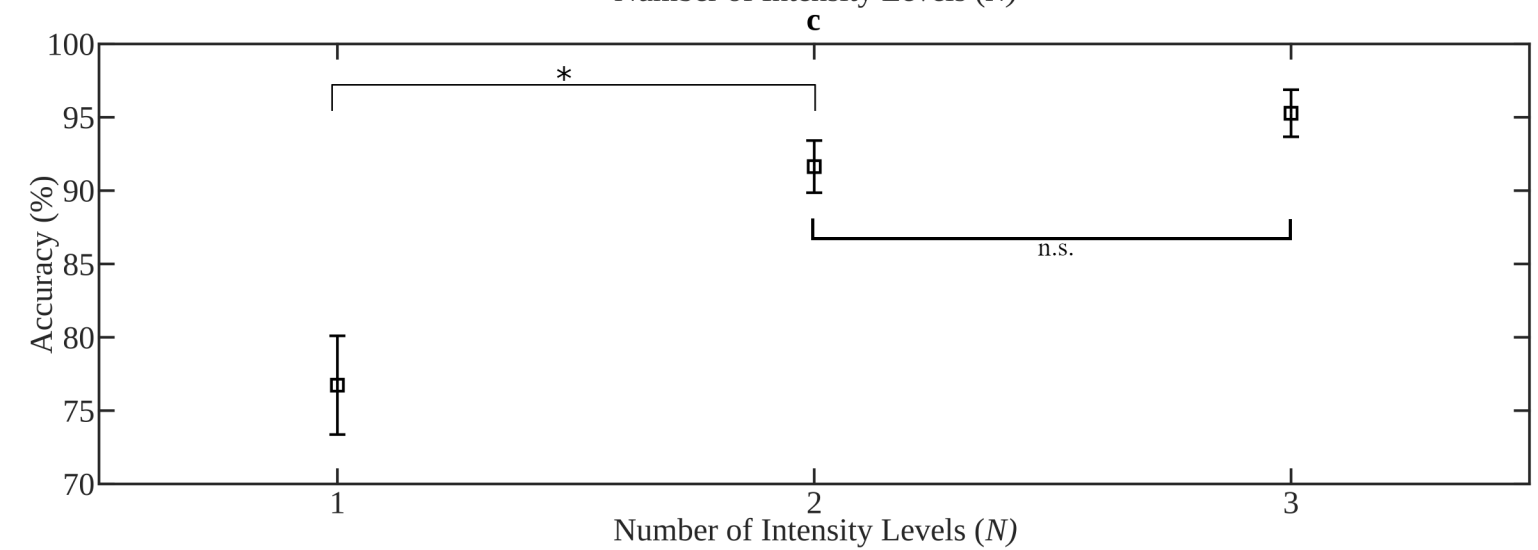

Supplement: Supplementary file 1 [file sensors-20-01613-s001.zip › sensors-725895 - SI/Figures/Supplimentary_N_vs_all_CI.pdf]

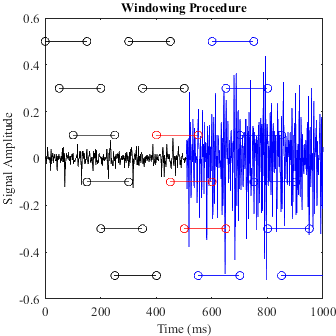

Supplement: Supplementary file 1 [file sensors-20-01613-s001.zip › sensors-725895 - SI/Figures/windowprocedure.png]
